# Extra‐pair paternity in birds

**DOI:** 10.1111/mec.15259

**Published:** 2019-10-31

**Authors:** Lyanne Brouwer, Simon C. Griffith

**Affiliations:** ^1^ Department of Animal Ecology & Physiology Institute for Water and Wetland Research Radboud University Nijmegen The Netherlands; ^2^ Department of Animal Ecology Netherlands Institute of Ecology (NIOO‐KNAW) Wageningen The Netherlands; ^3^ Division of Ecology and Evolution Research School of Biology The Australian National University Canberra ACT Australia; ^4^ Department of Biological Sciences Macquarie University North Ryde NSW Australia

**Keywords:** mating system, microsatellites, parentage, polyandry

## Abstract

Since the first molecular study providing evidence for mating outside the pair bond in birds over 30 years ago, >500 studies have reported rates of extra‐pair paternity (EPP) in >300 bird species. Here, we give a detailed overview of the current literature reporting EPP in birds and highlight the sampling biases and patterns in the data set with respect to taxonomy, avian phylogeny and global regions, knowledge of which will be crucial for correct interpretation of results in future comparative studies. Subsequently, we use this comprehensive dataset to simultaneously test the role of several ecological and life history variables. We do not find clear evidence that variation in EPP across socially monogamous species can be explained by latitude, density (coloniality), migration, generation length, genetic structuring (dispersal distance), or climatic variability, after accounting for phylogeny. These results contrast previous studies, most likely due to the large heterogeneity within species in both EPP and the predictor of interest, indicating that using species averages might be unreliable. Despite the absence of broadscale ecological drivers in explaining interspecific variation in EPP, we suggest that certain behaviours and ecological variables might facilitate or constrain EPP, as indicated by our finding that EPP was negatively associated with latitude within noncolonial species, suggesting a role of breeding synchrony. Thus, rather than focussing on general explanations for variation in EPP across all species, a future focus should be on how various aspects of ecology or life history might have driven variation in EPP among groups of species or populations of the same species. Hence, we argue that variation in EPP can be partly explained when taking the right perspective. This comprehensive overview, and particularly the dataset provided herein will create a foundation for further studies.

## 1 INTRODUCTION

**Figure 1:**
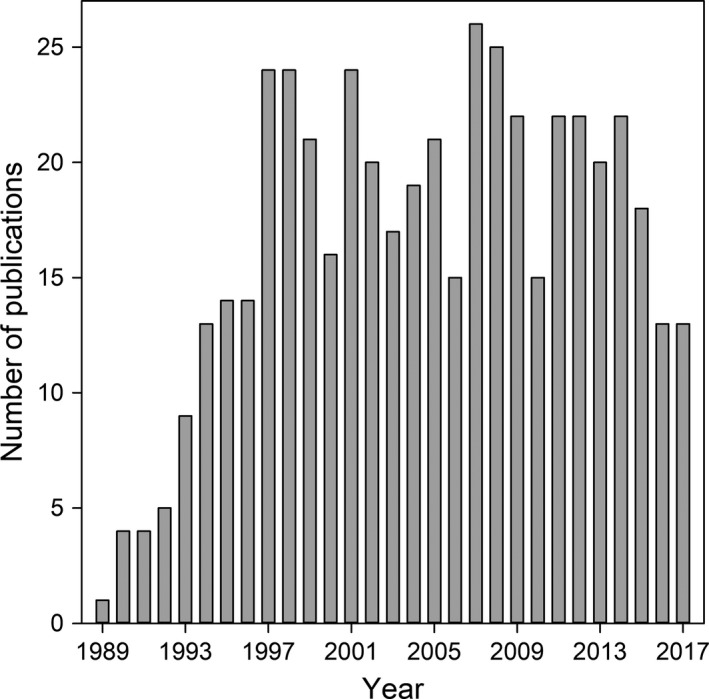
The number of publications per year reporting extra‐pair paternity rates in birds that were included in our dataset after using our rules of exclusion (*N* = 484 publications). Note that the number of publications for 2017 reflects studies published before 1 August 2017 only

The molecular study of parentage in birds is now into its fourth decade, with hundreds of studies following on from the first application of DNA fingerprinting in 1987 that provided clear evidence of mating outside the pair bond (extra‐pair paternity, EPP) in the house sparrow *Passer domesticus* (Burke & Bruford, 1987). By definition, extra‐pair paternity can only occur when there is a social bond between mates, and this is particularly prevalent in both socially monogamous species with biparental care (c. 81% of all species), and cooperatively breeding species (c. 9% of species) in which more than two adults will contribute care to a set of offspring (Cockburn, 2006). Since the mid 1990s, by which time the molecular techniques were well established, the number of new studies each year examining the occurrence and rates of EPP has remained more or less steady (Figure 1), reflecting a continued interest in this area of molecular ecology. Understanding the genetic mating system is essential for developing a proper understanding of natural, and sexual selection in this taxonomic group, in which it has long been clear that genetic polyandry was more commonplace than believed prior to the application of molecular techniques (Griffith, Owens, & Thuman, 2002). Indeed, a brief summary of the studies to date, finds that in 386 populations of 255 species of socially monogamous avian species with biparental care, genetic polyandry has been detected in 76% of species, with great variation in the level across surveyed species (Table S1). In 30% of these species EPP was rare (<5% broods contained EP offspring), while in a minority (13% of species), the majority of females engaged in this behaviour (>50% broods contained at least one EP offspring). On average, in those socially monogamous biparental species in which genetic polyandry has been found, 19% of offspring are found to have been sired by an extra‐pair male, in 33% of broods (Table S1).

These numbers are similar to those that characterised EPP in birds from a much earlier set of data (studies conducted up to 2001; reviewed by Griffith et al., 2002), suggesting that we were already able to characterise the broad patterns of genetic polyandry with approximately one third of the data that we now have. Nonetheless, it seems that after several decades of research, this is an appropriate time to take stock and ask: what has been done to this point; what do we know; and what remains to be understood?

Within any species or population, the biggest question that remains to be satisfactorily resolved is why some females have extra‐pair offspring while others do not. If anything, over the past couple of decades, we have moved further away from a consensus about the underlying motivation of females to engage in extra‐pair mating behaviour (Forstmeier, Nakagawa, Griffith, & Kempenaers, 2014). While the adaptive benefits of engaging in extra‐pair activity are obvious for males—extra‐pair paternity provides a route to increase reproductive success without the costs of care—the benefits to females remain hotly debated (Arct, Drobniak, & Cichoń, 2015; Drobniak, Arct, & Cichoń, 2015; Forstmeier et al., 2014; Nakagawa, Schroeder, & Burke, 2015). Engaging in extra‐pair mating behaviour is expected to be costly to females, due to search costs (Dunn & Whittingham, 2006), reduced investment by the social partner (Matysioková & Remeš, 2013), and the risk of sexually transmitted disease (Poiani & Wilks, 2000). The suggested counter‐balancing benefits to females include extra‐pair males providing females with extra food (Tryjanowski & Hromada, 2005), protection against predators (Gray, 1997), contributing parental care (Townsend, Clark, & McGowan, 2010) and providing insurance against infertility (Sheldon, 1994). However, the absence of obvious, and measurable direct benefits for the majority of species has seen a strong emphasis on the exploration of potential indirect genetic benefits, allowing females to enhance their offspring's genetic makeup.

Despite a sustained focus on the search for indirect genetic benefits to females (and their offspring), over time it has become apparent that good consistent evidence for this is lacking (Akçay & Roughgarden, 2007; Arnqvist & Kirkpatrick, 2005; Forstmeier et al., 2014; Hsu, Schroeder, Winney, Burke, & Nakagawa, 2015). It has also been argued that female participation in polyandry is a consequence of sexual conflict, with strong selection on polygynous behaviour in males supporting the existence of polyandry by females, either as a mechanism to reduce harassment by males (Arnqvist & Kirkpatrick, 2005) or through a tight genetic correlation between promiscuous behaviour in males and females (Forstmeier, Martin, Bolund, Schielzeth, & Kempenaers, 2011).

Despite the absence of consistent evidence for genetic benefits of EPP across species, one empirical pattern that has emerged over the past decade, is the frequent finding of a relationship between the genetic similarity of social partners and the incidence of extra‐pair offspring in broods (Arct et al., 2015, see below); or a significant difference in the level of genetic heterozygosity in within‐pair and extra‐pair offspring (reviewed in Griffith, 2010). Such patterns are consistent with ideas about genetic viscosity in populations and the effect of genetic polyandry on inbreeding and outbreeding (Hajduk et al., 2018; Lichtenauer, van de Pol, Cockburn, & Brouwer, 2019). Most of these studies are based on the application of codominant microsatellite markers, which whilst good for analysis of parentage, are a little more limited in assessing microgenetic population structuring. As such, the relationship between parentage and genetic diversity within and across populations, is one area that might be facilitated by the application of next‐generation molecular techniques, like SNPs, to study parentage. Although these will not necessarily improve our ability to detect EPP (Flanagan & Jones, 2019; Kaiser et al., 2017), such tools will provide a greater measure of genome wide genetic diversity and can provide more informative estimations of population parameters, like the degree of inbreeding/outbreeding, relatedness amongst individuals, and genetic microstructure within populations (Flanagan & Jones, 2019). In their recent review, Flanagan and Jones (2019) have demonstrated that a panel of around 100–200 SNP markers will provide the same resolving power as the typical panel of microsatellites, and can be achieved at a relatively cost‐effective price in systems for which there is no genomic data available (e.g., using RAD‐seq). As a result of these potential cost efficiencies, and the decreasing cost of next‐generation sequencing approaches, it is likely that such approaches may largely reduce the economic constraints of marker development for future studies. To date, relatively few innovative studies have taken a next‐generation sequencing approach to the analysis of parentage in birds (e.g., Kaiser et al., 2017; Thrasher, Butcher, Campagna, Webster, & Lovette, 2018; Weinman, Solomon, & Rubenstein, 2015; for overview see Flanagan & Jones, 2019), but this seems likely to change in coming years.

**Figure 2:**
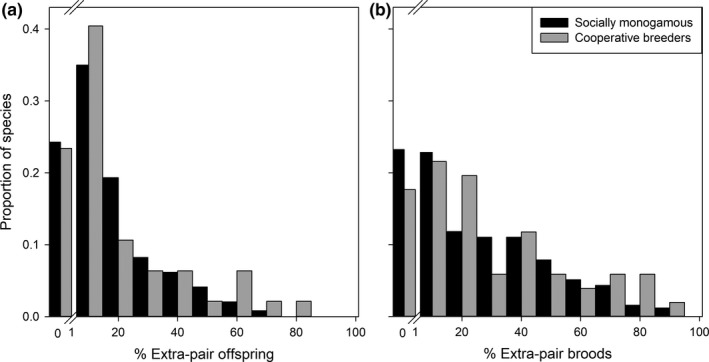
Histogram showing distributions of (a) percentage extra‐pair offspring, and (b) percentage of broods with at least one extra‐pair offspring for biparental socially monogamous and cooperatively breeding species

At a broader level, it remains unclear why EPP is absent in some species whereas it is common in others (Figure 2, Tables S1 and S2). The studies of extra‐pair paternity in birds provide the most extensive molecular evidence of mating system for any taxonomic group and this extensive literature has been the focus of many comparative studies (e.g., Biagolini, Westneat, & Francisco, 2017; Botero & Rubenstein, 2012; Westneat & Stewart, 2003). However, whilst comparative studies can provide important insight, they may be compromised by both biased sampling, and inaccurate estimates. The first aim of this review is to provide a complete overview of the peer‐reviewed studies conducted to date that report EPP rates, which will facilitate large‐scale comparative studies. Moreover, we highlight the biases in the dataset, as for correct interpretation of comparative studies it is crucial to be aware of the limitations of the existing data, and the way that data are extracted from that literature. In addition, we use this dataset to characterise the patterns of EPP across birds and test the effect of a number of proposed life history traits and broadscale ecological drivers—latitude, climatic variability and the role of habitat complexity.

## 2 A COMPLETE OVERVIEW OF 30 YEARS ON STUDIES REPORTING EPP

We collated published studies from the primary peer‐reviewed literature published before 1 August 2017, which reported rates on extra‐pair paternity in birds, therefore accounting for the first thirty years of data (the first study was published in May 1987, Burke & Bruford, 1987). We included all studies that were part of the earlier comprehensive review from Griffith et al. (2002) and also did a forward search by checking later studies that cited this, and a number of the earlier reviews (Petrie & Kempenaers, 1998; Westneat & Stewart, 2003). We also cross‐referenced with the data reported in recent meta‐analyses and comparative studies (Arct et al., 2015; Biagolini et al., 2017; Cornwallis et al., 2017) for any missing studies and did a final literature search in Google Scholar in August 2017. We set this cutoff so that the analyses presented above considered this full dataset. We excluded studies based on the following arguments: studies using an experimental approach that could have affected EPP rates and that did not provide estimates from a control group; EPP rates from captive populations or hybrids; studies based on allozymes; and nonpeer reviewed studies (Table S3). Furthermore, in the cases (of which there are many) in which multiple papers have focused on the same molecular data set, we examined the dates and study location to ascertain that the studies did indeed report estimates of the same population, and therefore only used a single study to represent this population (usually the study with the largest sample or the one that was clearest in characterising the molecular work and outcomes). After these exclusions, the remaining studies should provide the best quality estimates of the level of EPP in ecologically relevant situations. Variation between our reported rates and those in the original publication might sometimes exist. For example, we excluded cases of intraspecific brood parasitism from the sample of offspring, as such cases do not reflect infidelity of the pair female. Of the 577 publications we found, 484 remained in our dataset after applying our rules of exclusion and these reported 539 rates of genetic polyandry (Tables S1 and S2). A total of 38 studies did not estimate the proportion of extra‐pair offspring, but only reported estimates of the proportion of broods with at least one extra‐pair offspring (Tables S1 and S2).

We emphasize the need for an accurate standardized dataset that can be confidently used in subsequent comparative studies, and acknowledge that extracting data from the literature in this area can be somewhat difficult, particularly for species with complicated mating systems. For studies where we had difficulty in extracting numbers we have indicated so (see remarks in Tables S1 and S2). We cross‐referenced our dataset, as we were compiling it, with one of the larger ones used in a comparative analysis (Cornwallis et al., 2017) which was an updated version of an earlier dataset (Cornwallis, West, Davis, & Griffin, 2010). A number of discrepancies became apparent and this comparison helps to highlight the potential difficulties in extracting data from the literature. Many of the discrepancies were due to the fact that we reported the numbers for subsets of data that were nonexperimental, or from fully sampled pairs/groups, or because we excluded cases of intraspecific brood parasitism (from the sample size of offspring). However, other discrepancies were due to errors in sample sizes or simple, but critical, typographic errors in the earlier data (Cornwallis et al., 2017). For example, for a study on wood thrush *Hylocichla mustelina* (Evans, Woolfenden, Friesen, & Stutchbury, 2009) Cornwallis et al. (2017) listed the sample size of broods as 136 instead of 36, which leads to the level of EPP being 18% instead of 67%. In other species it is not clear how the error arose (e.g., for *Alauda arvensis* Hutchinson and Griffith (2007) they report a level of 51.8% extra‐pair broods when in fact it is 26.9%). As a result of the errors that we identified in the Cornwallis et al. (2010), Cornwallis et al. (2017) data set (see Table S4), and the fact that their dataset is large, reasonably high profile, and a likely source for future comparative analyses, we believe that it would be valuable to present a comprehensive and error‐free set of data on extra‐pair paternity. Consequently, we went to all of the original studies for every EPP estimate and double‐checked the numbers carefully. The dataset presented here is thus the most comprehensive and accurate available and should supersede any previous large collections of data on extra‐pair paternity. To facilitate future comparative studies and meta‐analyses, we recommend that future studies of extra‐pair paternity include all of the information summarised in Box 1.

BOX 1 **Guidelines for publishing future studies of EPP**

**Information that should always be included clearly in future studies of parentage**

- Basic metadata
  - *N* families sampled (i.e., sets of adults)
  - *N* broods sampled
  - *N* offspring sampled
  - *N* offspring found to be within‐pair and extra‐pair
  - *N* broods that contained extra‐pair offspring
- Contextual information
  - Location of study population (latitude and longitude) and indication of previous studies of that population
  - Clear indication of the social context of families. Whether they are socially monogamous, polyandrous, polygynous, or a cooperatively breeding group? If there is variation across the sample, then ideally the basic metadata should be broken down by category.
  - Indication of whether the focal families are part of experimental work, or represent a biased sample (i.e., late breeding birds). Basic metadata should be provided for control families separately.

**General approach**

Studies need to identify the social parents to be able to confidently exclude parentage. Forensic studies identifying the presence of multiple sires of a brood in the absence of social assignment are of no real value.

Observational studies should be written as such, and not written up as tests of hypotheses, particularly when only a single hypothesis is considered, whereas multiple factors could play a role in explaining variation in EPP rates.

## 3 VARIATION IN EPP: BREEDING SYSTEMS

If we consider all avian species for which genetic polyandry has been determined, we find that extra‐pair offspring has been detected in 75% of the 342 sampled species (Table S5 provides species level estimates of EPP). However, avian species have an array of different breeding systems, categorised by the social relationships between males and females within a population during reproduction, and particularly the pattern of parental care (Cockburn, 2006). The most prevalent mating system in birds is social monogamy with biparental care, and this is found in 81% of species (Cockburn, 2006). Correspondingly, a similar percentage of studies (77%, 371 publications) in our dataset focused on species with a socially monogamous breeding system (Table S1), and EPP was found to be present in 76% of these socially monogamous species (Figure 2a).

Interestingly, the very highest reported EPP rates have been found in cooperatively breeding species: 81.4% of offspring in Australian magpies (*Gymnorhina tibicen*, Hughes et al., 2003) and 71.8% in superb fairy‐wrens (*Malurus cyaneus*, Cockburn & Double, 2008; Table S2). Indeed, comparing the distributions of EPP rates shows that there are proportionally more cooperatively breeding species with EPP levels >30% (20% of cooperatively breeding species, Figure 2a) than those socially monogamous species with biparental care (14% of socially monogamous species, Figure 2a), although this difference was not statistically significant (*χ* ^2^ = 1.2, *p *= .28). This contrasts starkly to the situation presented by Cornwallis et al. (2010) in a study testing their interpretation of the monogamy hypothesis. This hypothesis, proposed by Boomsma (2007) to explain the emergence of cooperation and eusociality in invertebrates, suggests that such behaviour will be more likely to arise from lineages that breed monogamously, because that will mean that social groups are composed of close kin, thereby facilitating kin selection. However, the basic assumption of the monogamy hypothesis of obligate genetic monogamy is rarely, if ever, met in birds, because even in situations in which genetic monandry exists within a reproductive attempt, birds are largely iteroparous and females will be genetically polyandrous over a lifetime (e.g., Warrington, Rollins, Russell, & Griffith, 2015).

The relatively high incidence of extra‐pair paternity in cooperatively breeding birds (Figure 2) makes sense, because the presence of subordinate helpers might reduce the costs for females to engage in extra‐pair mating, because helpers can compensate for the reduced investment in parental care by males when they suspect they have been cuckolded (Mulder, Dunn, Cockburn, Lazenbycohen, & Howell, 1994). More importantly, cooperative breeders do not just consist of kin groups (one of the key assumptions made in the study by Cornwallis et al., 2010), but encompass huge variation in social systems (Cockburn, 1998; Díaz‐Muñoz, DuVal, Krakauer, & Lacey, 2014). However, this also complicates comparisons among species. For example, females in cooperatively breeding species might mate with multiple group members to assure their contribution to parental care. In such cooperative polyandrous systems there is often no obvious dominant pair present or this is hard to asses based on behavioural observations, and therefore reported estimates of EPP might represent multiple‐mating within a social bond, i.e., genetic polyandry but no infidelity. Furthermore, in such polyandrous pairings, multiple mating may not be represented in paternity estimates if only one of the (nonpair bonded) males sires offspring.

In a similar way, many brood parasites do not form pair bonds and additionally present the difficulty that they often lay a single egg in multiple nests, complicating the assessment of the proportion of extra‐pair young in a brood. For example, in Horsfield's bronze cuckoos (*Chalcites basalis*) half‐siblings were detected, but these were not the result of extra‐pair mating but rather from a bimodal pattern in the timing of breeding by females that allowed males to be sequentially monogamous (Langmore, Adcock, & Kilner, 2007). Thus, to avoid difficulties with the definition of what constitutes a pair bond and mating outside the social pair bond, we restricted a large part of our analyses to socially monogamous species with biparental care, and additionally provide the broad scale patterns with respect to sampling and geographic bias for the complete dataset.

## 4 VARIATION IN EPP: PHYLOGENY AND GEOGRAPHY

**Figure 3:**
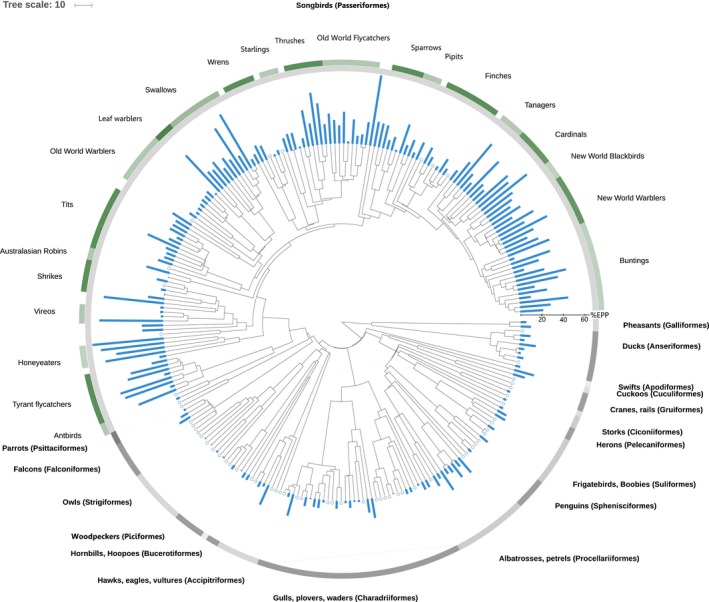
A phylogenetic tree showing the average proportion of EPP (blue bars) for all sampled socially monogamous bird species, with zero EPP indicated by an empty circle. Orders (in bold) and passerine family names are given to indicate their location in the tree. Taxonomy was based on information provided in Jetz et al. (2012), drawn using a tree based on the Hackett backbone (Hackett et al., 2008) and the Interactive Tree of Life website (Letunic & Bork, 2016)

Over the past 30 years, the main focus of studies reporting EPP rates has been to try and explain why some individuals, populations or species have higher EPP than others. Considering all socially monogamous species in our dataset shows there is strong phylogenetic bias in EPP rates, with species with particularly high or low EPP rates respectively being clustered in the phylogeny (Figure 3). Particularly obvious is the contrast between passerines (songbirds) and other orders, although there is a lot of variation within the passerines too (Figure 3). This complicates comparisons among species, because a significant component of the variation in EPP does not necessarily reflect contemporary selection pressures, but was established in the ancient evolutionary history of a clade (e.g., Griffith et al. (2002) reported that >50% of the interspecific variation of socially monogamous species occurred at the level of the family or order). In our analyses of the new and larger dataset, we find that 39% of the interspecific variation in EPP of socially monogamous species can be attributed to the level of the family or order (proportion variance explained by family and order in a Generalized Linear Mixed Model (GLMM) with the number of extra‐pair offspring (EPO) vs. no. within‐pair offspring (WPO) per species fitted as a binomial response and identity of family and order included as random intercepts, Model 1 in Appendix S1). Therefore, despite the increase in the number of species and orders sampled in the past 16 years, the reduction in strength of the phylogenetic signal is minimal and explaining interspecific variation in EPP remains difficult.

**Figure 4:**
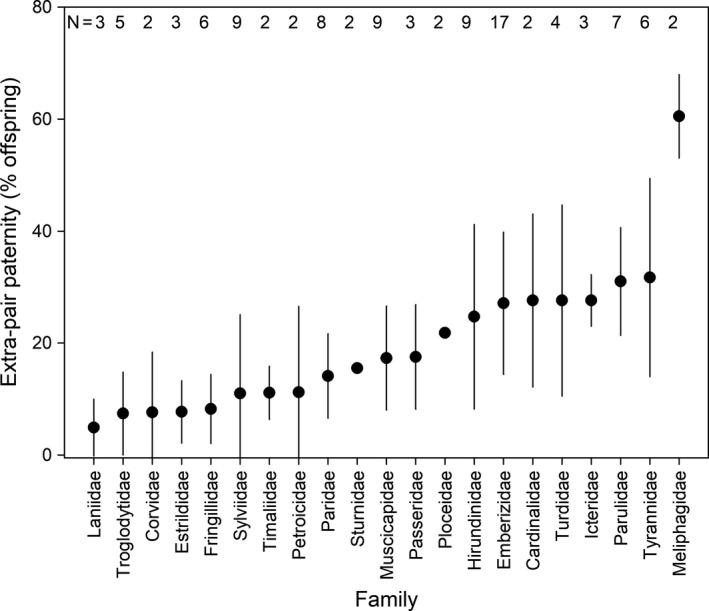
Average (±*SD*) EPP rates for biparental socially monogamous passerine families. Only studies based on ≥50 offspring and families where multiple species were sampled were included. Numbers on top indicate the sample sizes (number of species)

This broad analysis tells us that species within a family tend to have a similar level of EPP, and that there is a difference in the average level across families. Even when only considering passerine families for which multiple socially monogamous species with biparental care have been sampled, we find that family‐level variation in EPP is remarkable, although interestingly EPP is not completely absent in any of these families (Figure 4; to reduce potential bias, 31 studies with *N*_offspring_ < 50 were excluded here). Whilst there are clearly differences across families within the passerines, the Meliphagidae really stand out, with over 60% of the offspring sired by extra‐pair males. Species within this family of honeyeaters are extremely territorial and it has been suggested that while males are busy defending their territory against intruders, females are relatively unconstrained and have the opportunity of cryptic extra‐pair mating without males being aware of this and thus suffering reduced costs (Ewen, Ciborowski, Clarke, Boulton, & Clarke, 2008). EPP is also particularly common in the Hirundinidae (swallows & martins), a family of birds that has been a model system for studies on sexual selection (Lifjeld et al., 2011; Møller & Birkhead, 1994), and in which the foraging strategy (aerial hawking for flying insects), may also make it very difficult for a male to constrain mating opportunities for his partner. In contrast, there are a number of families in which EPP is rare (for example the carnivorous Laniidae [shrikes], and the granivorous Estrildids [grass finches]), with a few different ideas suggested to explain the low EPP levels in the species examined in these families. At one end of the scale the cost of physical retribution in shrikes has been suggested to maintain a faithful pair‐bond (Valera, 2003); whilst in Estrildids it has been suggested that fidelity is maintained by the selection on the development of strong and lasting social partnerships in an ecologically unpredictable situation (del Hoyo, Elliott, & Christie, 2010). Probably the only consistent pattern across the studies characterising species levels of extra‐pair paternity is that a story can usually be developed to explain a level that is deemed to be either low or high, and that without much more experimental work it is very difficult to evaluate the different ideas proposed. One of the issues apparent from our reading of the hundreds of largely observational studies of extra‐pair paternity, is that the vast majority of observational papers are written as tests of a single hypothesis (e.g., good genes, breeding synchrony etc, see Table 1 for overview), whereas multiple factors probably play a role. In future, authors should be discouraged by editors and reviewers from taking simple observational datasets and over‐interpreting them or over‐analysing them to test a single hypothesis. The level of extra‐pair paternity is a characteristic of a species' natural history and there is nothing wrong with presenting it as such in a concise and simple way.

**Table 1:** Overview of adaptive and nonadaptive hypotheses proposed to explain variation in extra‐pair paternity

Hypothesis	Description	References
*Adaptive*		
Fertility insurance	Females seek EPP in order to guard against infertility in their own social mate, but females have no way of assessing the fertility of males	Wetton and Parkin (1991)
	Females seek EPP in order to guard against infertility in their own social mate, and females are able to assess male fertility through phenotypic cues	Sheldon (1994)
Genetic diversity	Females seek EPP to maximize genetic diversity among their offspring, but females cannot assess the extent of genetic similarity between themselves and males	Westneat et al. (1990) and Williams (1975)
Genetic compatibility	Females seek EPP to maximize genetic compatibility between themselves and the father of the offspring, and females can assess the extent of genetic similarity between themselves and males through phenotypic cues	Kempenaers, Congdon, Boag, and Robertson (1999) and Tregenza and Wedell (2000)
Good genes	Females seek EPP to obtain good genes for their offspring, and females can assess the genetic quality of males through phenotypic cues	Birkhead and Møller (1992), Hamilton (1990); Møller (1988), and Westneat et al. (1990)
Direct benefit	Females seek EPP to obtain (nongenetic) resources for their offspring, and females can assess the resources held by males	Burke, Davies, Bruford, and Hatchwell (1989), Colwell and Oring (1989), and Wolf (1975)
Convenience polyandry	Females agree to mate with multiple males only to avoid the costs arising from male harassment	Thornhill and Alcock (1983)
*Nonadaptive*		
Life history	Lower survival will result in higher EPP, because the risk of retaliation by males with a short lifespan is low, as it is not adaptive for them to abandon a reproductive event	Wink and Dyrcz (1999)
Density	The encounter rates between individuals affect the rate of EPP	Westneat et al. (1990)
Breeding synchrony (male assessment)	Breeding synchronously facilitates simultaneous comparison of different males	Westneat et al. (1990)
Breeding synchrony (male trade‐off)	Synchrony results in trade‐off for males between mate guarding and EP mating	Stutchbury and Morton (1995)
Constrained female	Females are constrained in pursuing EPP, because it can result in retaliation by the male, leading to reduced paternal care when the male loses confidence in paternity	Birkhead and Møller (1996)
	Females are constrained in pursuing EPP, because they are energetically limited to seek EPP	Gowaty (1996)
Byproduct of selection	Nonadaptive female extra‐pair mating is caused by alleles under strong positive selection in males, because they enhance male extra‐pair paternity gains	Forstmeier et al. (2014) and Halliday and Arnold (1987)
Environmental constraint	Males are constrained in gaining EPP, because of low food availability	Johnsen and Lifjeld (2003) and Kaiser et al. (2015)
Paternal trade‐offs	Males trade‐off EP mating and paternal care	Kaiser et al. (2015), Trivers (1972), and Westneat et al. (1990)
Constrained male	Males trade‐off their energetic demands between mate‐guarding and pursuit of extra‐pair copulations	Kaiser et al. (2015) and Westneat et al. (1990)

**Figure 5:**
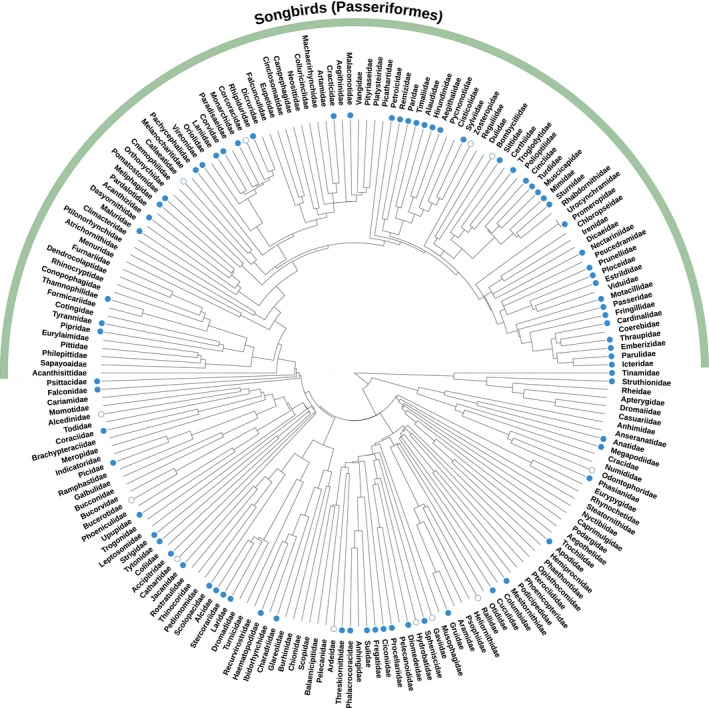
A phylogenetic tree showing the distribution of availability of EPP rates for all 194 bird families reported in Jetz et al. (2012). Blue circles indicate families in which EPP has been determined in at least one species, with filled circles indicating EPP > 0 and open circles indicating EPP = 0. Drawn using a tree based on the Hackett backbone (Hackett et al., 2008) and the Interactive Tree of Life website (Letunic & Bork, 2016)

It is apparent from our analysis of the relatively large amount of data across all avian species that our understanding of the variation in the overall rates of EPP will probably be affected by biased sampling. These biases will be driven by the uneven distribution of sampling both phylogenetically and spatially. There are significant differences across avian families (as above), and many families are yet to be investigated at all, with others over‐represented in the literature to date. Despite the large number of studies reporting EPP rates (or proportion of broods with EPO) on a seemingly large number of species (342 species in total), this represents under 4% of the avian biodiversity, and for the majority (56%) of the 194 bird families reported in Jetz, Thomas, Joy, Hartmann, and Mooers (2012), EPP rates have not yet been estimated in any species (Figure 5). The families that have been sampled are typically clustered within the avian phylogeny, and even when considering the passerines, only 47% of families have been sampled (Figure 5). It is important to be aware of this bias when performing comparative studies because many ecological, and life history variables that have been hypothesized to predict variation in EPP typically also covary with phylogeny and thus might be confounded with EPP rates.

**Figure 6:**
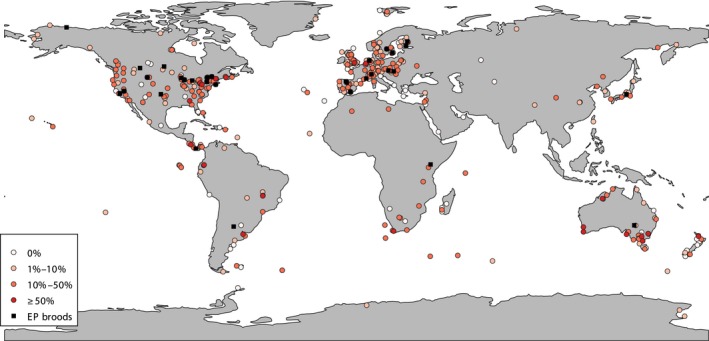
Map showing locations of studies reporting extra‐pair paternity in birds. Levels of EPP at the offspring level are indicated with coloured circles, studies only reporting EPP at the level of the brood are indicated with a square. Map drawn using qgis 3.4 (QGIS Development Team, 2018). For studies that did not report their exact location, approximate coordinates were derived from Google Maps on the basis of the description provided (see Appendix S1)

The second source of potential bias is driven by the uneven spatial distribution of estimates. This will be a problem if levels of polyandry are related to environmental parameters that vary across the world. There is a strong geographical bias towards studies conducted in northern hemisphere (temperate) regions. Indeed, considering the data collected across all breeding systems we found that 63% of studies considering EPP were conducted in Europe or North‐America and only 7% of studies were on species from either South‐America or Africa (Figure 6), which are centres of global avian biodiversity (e.g., Hawkins, Porter, & Felizola Diniz‐Filho, 2003). The data available show that in biparental socially monogamous terrestrial species, there are differences in the rate of EPP across the continents, with, on average, much lower levels of EPP in Europe (11%) compared to Africa (20%), Australasia (23%) and South‐ and North‐America (17% and 20%, respectively; mean of average EPP per species, Appendix S1). There is an expectation that the fundamentally different patterns of ecology and life history from tropical to temperate areas will drive some differences in polyandry. For example, Australian species have much more flexible patterns of breeding phenology than European birds (Englert Duursma, Gallagher, & Griffith, 2017), and the longer and less predictable breeding seasons may favour more sustained and stronger pair bonds (Botero & Rubenstein, 2012). The distribution of studies illustrated in Figure 6, makes it clear that we know a lot more relatively about EPP in the birds of Western Europe, and the eastern part of North America, than other species globally. It is hard to imagine that the avifauna of these two areas is disproportionately more interesting than that elsewhere, and this probably reflects the accessibility of these species to those working in this research area, both scientifically and geographically. Developing a broader geographical representation of species should be one of the main priorities for future work.

**Figure 7:**
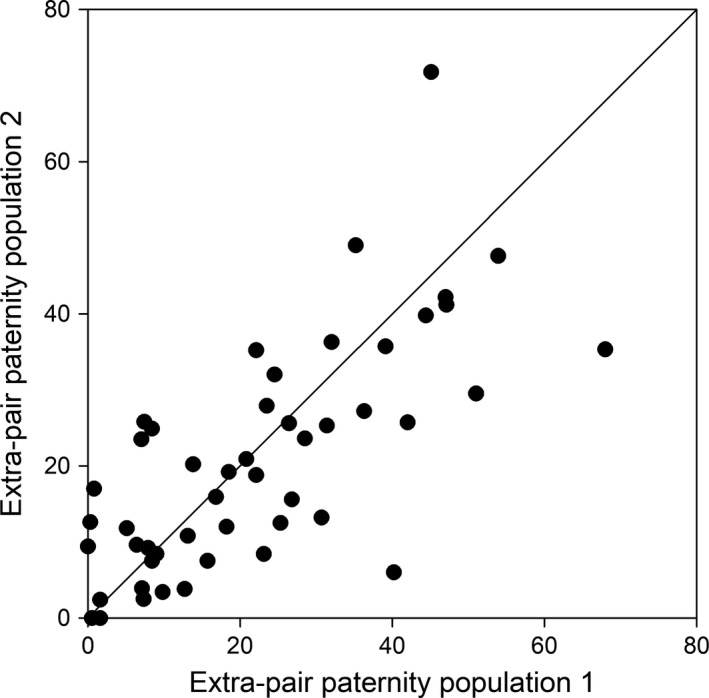
Repeatability of extra‐pair paternity rates for two randomly selected populations of each of the 49 bird species that have been sampled in more than one population

Given that for 77% of the sampled species (264 out of 342), there is only one population estimate (from a single study) for the rate of EPP at the offspring or brood level available, it would be good to understand the extent to which a single measure represents a species well. Plotting EPP rates for two randomly selected populations (with *N*_offspring_ ≥ 50) of the 49 species that have been sampled in more than one population showed that some estimates were very similar, but there is considerable variation (see Figure 7). Considering the complete dataset there was significant repeatability of EPP rates at the species level: *R* = 0.24 ± 0.03 (estimated using package rptR (Stoffel, Nakagawa, & Schielzeth, 2017) on GLMM with no. EPO vs. no. WPO fitted as binomial response with logit link and identity of species and population included as random intercepts, Model 2 in Appendix S1). However, although significant, these results show that repeatability is rather low, so although the level of EPP in a species can be estimated on the basis of a single estimate, this will not be very accurate. Indeed, 24% of the variation among the 49 species that have been sampled in multiple populations could be attributed to variation at the population level, with a large part (87%) of this variation due to within‐population variation (but note that variation within and among populations is hard to disentangle since the majority of population estimates is based on a single study; GLMM with no. EPO vs. no. WPO fitted as binomial response with logit link and identity of species, population and study included as random intercepts, Model 3 in Appendix S1).

Although the population‐level variation may partly be driven by the heterogeneity in the approaches taken by different studies, and the quality of the estimate (based on molecular methods and sampling power), part of the variation could be due to temporal variation in demography or social environment (Maldonado‐Chaparro, Montiglio, Forstmeier, Kempenaers, & Farine, 2018; Reid, Duthie, Wolak, & Arcese, 2015). Temporal variation is hard to disentangle from other variation in the dataset, since studies usually combine multiple years of data into a single estimate of EPP. Ecological differences may also explain variation at the population level. For instance, variation in EPP rates among populations might correlate with breeding density, but at the same time variation in density might be due to variation in food abundance that can also affect EPP rates directly, as low food availability might constrain females in seeking EPP (Westneat & Mays, 2005) or result in a male trade‐off (Kaiser, Sillett, Risk, & Webster, 2015). As emphasized previously, experiments should be carried out to determine the causality of such ecological factors (Griffith et al., 2002), but these remain rare (Table S3).

A fruitful approach to explain interspecific variation, is a comparative approach of multiple species from a single family. This approach was recently adopted by studies on Emberizid sparrows (Bonier, Eikenaar, Martin, & Moore, 2014) and the Maluridae (fairy‐wrens and relatives; Brouwer et al., 2017). In Emberizid sparrows an interaction between latitude and elevation with EPP was found among 24 populations and 12 species. EPP rates decreased with elevation at higher latitudes, but increased with latitude, most markedly at lower elevations (Bonier et al., 2014). These results suggest there is a trade‐off between breeding synchrony facilitating EPP and the need for male parental care, which might be particularly strong at high‐latitude, high‐elevation sites, where breeding seasons are short (Bonier et al., 2014). In the Maluridae it was shown that several different hypotheses explain rates of EPP at different levels of variation (Brouwer et al., 2017). Females had higher EPP in the presence of more helpers, more neighbours, or if paired incestuously. Furthermore, higher EPP occurred in years with many incestuous pairs, populations with many helpers and species with high male density or in which males provide less care (Brouwer et al., 2017). Excluding bias due to broader phylogenetic variation thus helped identify variables important in explaining variation in EPP among species and populations. However, such an approach is only suitable when sufficient species of the same family have been sampled and also raises a new challenge: closely related species often show limited variation in both their EPP rates and the predictor of interest, reducing the power to detect clear patterns. The availability of increasingly well resolved molecular phylogenetic trees for the majority of species (Jetzet al., 2012) might open up new possibilities for comparative studies investigating interspecific variation in EPP while accounting for phylogeny.

## 5 VARIATION IN EPP: BROADSCALE ECOLOGICAL DRIVERS

### 5.1 Habitat complexity

**Figure 8:**
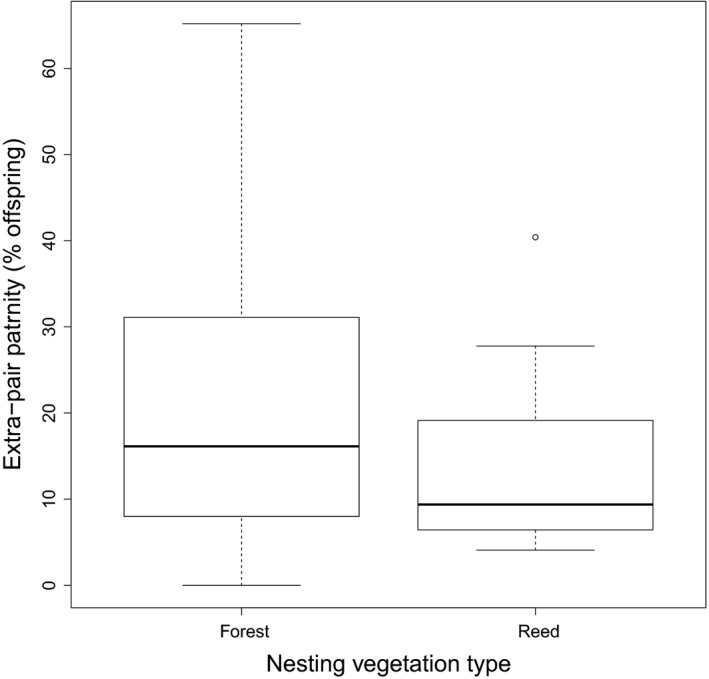
Boxplot showing the percentage of extra‐pair offspring for species nesting in ‘forest’ (*N* = 99 species) and ‘reed’ type (*N* = 10 species) vegetation (Table S5). The box plots show the median, 25th and 75th percentiles; the whiskers indicate the values within 1.5 times the interquartile range and the circle is an outlier

EPP can arise through different behavioural mechanisms, for example through females making forays outside her own territory to seek extra‐pair matings (Gray, 1997; Sheldon, 1994) or through intruding males that make forays onto the territory of a breeding pair (Sherman & Morton, 1988; Westneat, 1994). Females will probably be selected to avoid detection by their male partner to reduce costs like harassment (Low, 2005; Westneat & Stewart, 2003) or the loss of a partners' contributions to parental care (Griffin, Alonzo, & Cornwallis, 2013). Whilst it is important to recognise that there is very little data available on actual male and female extra‐pair mating behaviour (our understanding of genetic polyandry in birds comes from the molecular evidence left by those extra‐pair copulations that fertilise eggs; Griffith, 2007), we can speculate about the likely drivers of extra‐pair behaviour. The togetherness of the male and female partners, spatially and temporally during their breeding attempt, may influence EPP, and this may be affected by habitat complexity. The extent of covert extra‐pair behaviour might be facilitated in complex habitats in which partners are visually occluded (Sherman & Morton, 1988). In such habitats, females would also have greater opportunity to escape from male mate‐guarding (e.g., Mays & Ritchison, 2004), a behaviour which is positively associated with a male's share of paternity across species (Harts, Booksmythe, & Jennions, 2016). Also, in complex habitats, intruding males will be less easy to detect and extra‐pair copulations can take place in seclusion (e.g., Tryjanowski, Antczak, & Hromada, 2007). However, within‐species analyses failed to find support for the effect of habitat structure on the incidence of EPP (Ramos et al., 2014; Westneat & Mays, 2005). Although in blue‐footed booby (*Sula nebouxii*) the presence of obstacles was associated with EPP, suggesting that male access to females was constrained by obstacles to locomotion within the nesting colony (Ramos et al., 2014). A recent comparative study also failed to find support for a role of habitat complexity, tested using five different vegetation stratifications across socially monogamous species (Biagolini et al., 2017). Although this latter study accounted for phylogeny, vegetation stratifications are typically confounded with phylogeny and considering all species may have confounded the two. For example, species like seabirds which typically have low EPP (Figure 3), also typically nest in areas with little vegetation such as small oceanic islands. Therefore, we have conducted a phylogenetic analysis of a more restricted dataset comparing just species that inhabit key terrestrial habitats that vary in a way that occludes visual contact between partners. We predicted that it would especially be the reed, cat‐tail and sedge type vegetation that would facilitate EPP as these types of dense vegetation impair visual contact at very short distances. However, restricting the dataset to just socially monogamous passerines showed that species nesting in reed type vegetation did not have higher EPP than species nesting in forest type vegetation (Figure 8; Bayesian phylogenetic mixed model (BPMM), *β *= 0.18, 95% CI [−1.06 to 0.65], *P*_mcmc_ = 0.67; Model 4 in Appendix S1, see for data Table S5). Thus, despite the surreptitious nature of extra‐pair copulation behaviour and the suggestion that females try to avoid detection by their mates, there is no evidence to date that vegetation and habitat types play a role in determining the level of EPP across species. Furthermore, differences in behaviour among species might make vegetation type unimportant. For example, as discussed above, the foraging style of the Hirundinidae may hinder continuous monitoring of the fertile female. Likewise, female superb fairy‐wrens have been shown to engage in predawn forays for extra‐pair copulations (Double & Cockburn, 2000). New bird tracking technology may provide further insight into the further existence of such behaviour. For example, a recent study of the yellow‐breasted chat (*Icterina virens*) found females making extraterritorial forays at night during the fertile period (Ward, Alessi, Benson, & Chiavacci, 2014). The inherently cryptic nature of nocturnal forays (due to the darkness) may render habitat complexity irrelevant as a key determinant of EPP.

### 5.2 The role of breeding synchrony, migration, and climate on EPP: Can latitude explain it all?

Breeding synchrony was one of the first hypotheses proposed to explain variation in EPP across species (Stutchbury & Morton, 1995). Higher levels of breeding synchrony have been predicted to result in lower EPP as a result from a trade‐off between searching for extra‐pair matings and parental care (Birkhead & Biggins, 1987; Ims, 1990; Westneat, Sherman, & Morton, 1990). In contrast, it was suggested that synchronous breeding allows for the simultaneous assessment of fertile males thereby facilitating the opportunity for females to gain better genes for their offspring through extra‐pair mating (Lifjeld, Slagsvold, & Ellegren, 1997; Stutchbury, 1998; Stutchbury & Morton, 1995). The latter idea led to the suggestion that tropical species, which have longer breeding seasons (Englert Duursma et al., 2017; MacArthur, 1964) and thus breed more asynchronously than temperate species, should have lower EPP and subsequently, that latitude could explain variation in EPP. A meta‐analysis using phylogenetically independent standardized contrasts by Spottiswoode and Møller (2004) indeed showed that absolute latitude was positively associated with EPP across species (absolute latitude because it is the absolute distance from the equator that predicts synchrony, Spottiswoode & Møller, 2004). However, many other factors are known to be associated with latitude, for example environmental factors like primary productivity and, climate, and life history traits, like annual adult survival and migration (Cardillo, 2002; Gillman et al., 2015; Muñoz, Kéry, Martins, & Ferraz, 2018). Being migratory could affect behavioural parameters that might be relevant to mate choice such as highly constrained breeding phenology (and hasty establishment of social partnerships), or condition‐dependent arrival times that may constrain or enhance the ability to choose a partner (Spottiswoode & Møller, 2004). Indeed, migratory species have higher EPP than sedentary species, but also occur at relatively high latitudes, thus the observed association between latitude and EPP could be a result of differences in migration rather than breeding synchrony (Spottiswoode & Møller, 2004).

More recently, Botero and Rubenstein (2012) reported that species that breed in environments with greater within‐year variance in temperature are more likely to have extra‐pair offspring. Their interpretation was that EPP allows for greater reproductive flexibility, allowing individuals to select optimal partners in different ecological conditions (Botero & Rubenstein, 2012). However, like migration, within‐year variance in temperature also covaries with latitude, and thus the observed pattern between interspecific variation in EPP and temperature seasonality could also be explained by other factors. Here, we use our comprehensive dataset on biparental socially monogamous species to simultaneously test the role of latitude, key life history traits, and ecological variables that have all previously been suggested to explain variation in EPP (see also Table 1), while accounting for phylogeny in a BPMM (Model 5 in Appendix S1). We tested the following predictors: absolute latitude (as a proxy for synchrony) of each study location; climatic variability (seasonality in temperature and rain, derived from worldclim [Fick & Hijmans, 2017] or the nearest weather station [see Appendix S1]); maximum yearly dispersal distance, as an indication of the amount of genetic structuring, which has been shown to be positively correlated with EPP among 33 species (measured as pair genetic similarity [Arct et al., 2015]); coloniality (yes/no), to test the role of breeding density; generation length; and migration (yes/no; for details see Appendix S1). As previous studies have shown negative associations between latitude and EPP within species (Møller & Ninni, 1998), but positive associations among species (Spottiswoode & Møller, 2004), we included the mean latitude of a species as a predictor at the species level, whereas the deviation of the mean was used as a predictor for the within‐species effect of latitude (within‐subject centring; van de Pol & Wright, 2009). In addition, because facilitation of EPP by the simultaneous assessment of fertile males according to the breeding synchrony hypothesis might be particularly important in colonial species, we included an interaction between latitude (the proxy for synchrony) and coloniality.

**Figure 9:**
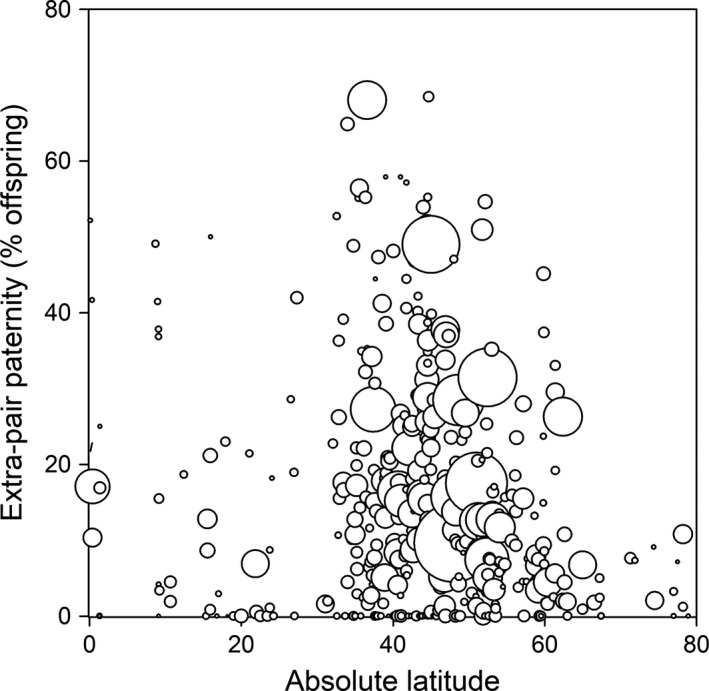
The relationship between extra‐pair paternity and absolute latitude, with each data point reflecting one of the 403 studies on biparental socially monogamous species. The size of the symbols indicates the sample size

Our results show that in contrast to a previous study (Spottiswoode & Møller, 2004), that was based on a smaller dataset (186 species compared to the 403 records from 245 species analysed here), absolute latitude does not positively correlate with EPP rates across socially monogamous species, but instead shows a slight negative association (but note the wide 95% CI, Figure 9, Table 2). However, absolute latitude was significantly negatively associated with EPP rates within species, but only for noncolonial species (Table 2). Thus, within noncolonial species, populations at higher latitudes had lower levels of EPP. Additionally, in contrast to previous results (Botero & Rubenstein, 2012; Spottiswoode & Møller, 2004) we did not find evidence for an association between EPP and migration or within‐year variation in temperature (Table 2). Furthermore, we did not find evidence for an association between EPP and generation length, coloniality, dispersal distance, or seasonality in rainfall. The contrasting results with previous work might simply be the result of the increased sampling, particularly with respect to latitude. However, there are several other explanations, worthy of further discussion.

**Table 2:** Results of a phylogenetic mixed model of several life history traits and ecological variables on the proportion of extra‐pair offspring of socially monogamous species (*N* = 403 studies, Table S1, except for 12 studies with missing data, see Appendix S1) showing the posterior means with 95% credible intervals for the standardized predictor variables (z‐scores) on the logit scale

Fixed effects	Posterior mean (95% CI)	*p*‐Value
Intercept	−3.37	—
Absolute latitude_between_	−0.16 (−0.35 to 0.04)	.11
Absolute latitude_within_	−0.09 (−0.16 to −0.03)	.006
Migratory	0.17 (−0.05 to 0.38)	.13
Colonial	0.01 (−0.24 to 0.21)	.95
Generation length	−0.24 (−0.60 to 0.13)	.19
Dispersal distance	−0.01 (−0.16 to 0.15)	.94
Temperature variability	0.07 (−0.07 to 0.21)	.29
Rain variability	−0.04 (−0.16 to 0.09)	.57
Absolute latitude_within_ × Coloniality	0.09 (0.02 to 0.16)	.005
Absolute latitude_between_ × Coloniality	0.05 (−0.13 to 0.23)	.62
Random effects		
*σ* ^2^_Phylogeny_	2.67	
*σ* ^2^_Species_	0.46	
*σ* ^2^_Population_	0.04	

First, by including all studies as data points in our analyses, we included variation that exists within populations and species. In previous analyses attempting to explain interspecific variation in EPP (e.g., Botero & Rubenstein, 2012; Spottiswoode & Møller, 2004) species averages were used for both EPP and the predictor of interest (e.g., latitude, climatic variation). However, some species occur over very large geographic areas, complicating the estimation of a single predictor per species. For example, the breeding range of the barn swallow extends over most of the Northern hemisphere and our dataset shows that temperature seasonality shows a 1.7‐fold difference over its range, indicating that calculating a mean EPP of the sampled populations and temperature variability over its entire range is unlikely to give a reliable estimate for the species as a whole. Whilst this is an extreme case, the same issue applies to many other species. It is more appropriate to take an estimate of the predictor of interest at the location for which EPP was measured, given the heterogeneity that we have demonstrated across populations of a species. We acknowledge that the same issue might apply for the estimates of generation length and dispersal distance in our analyses, which might vary among populations (although this variation is probably much smaller compared to the interspecific variation in such traits). Also, more accurate variables for density and migration might be better predictors for variation in EPP rather than the binary variables used here. For example, whilst we have found that coloniality or noncoloniality was a powerful predictor in an interaction with latitude, future work might want to better parameterise breeding density. Similarly, whilst previous work has found a difference in EPP between migratory and resident species, a rough species estimate of migration distance was also associated with EPP levels (Spottiswoode & Møller, 2004), thus future work may want to consider the actual distance that migratory populations travel.

**Figure 10:**
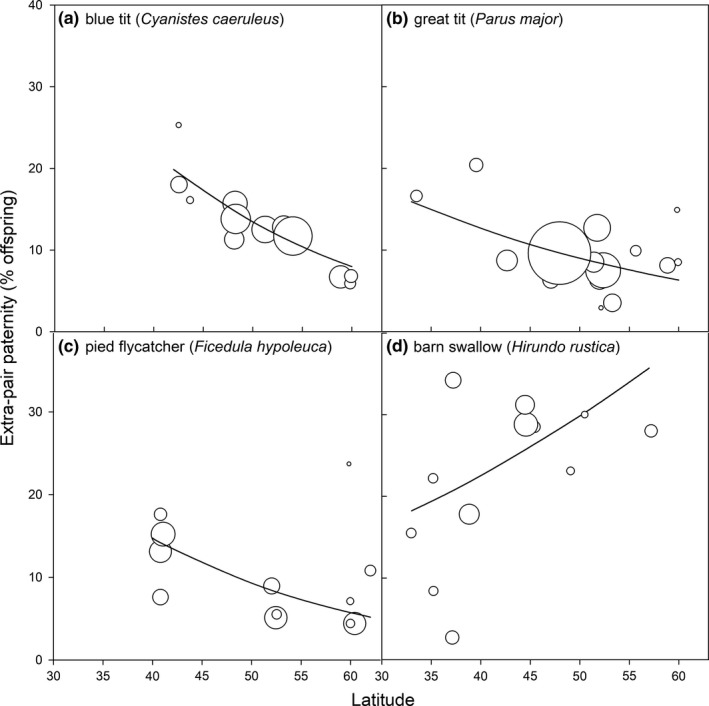
The relationship between extra‐pair paternity and latitude for four socially monogamous species that have been sampled in at least 10 different populations. Trendlines show the predictions from a GLM testing the association between latitude and the proportion of extra‐pair offspring for each species (see Model 6 in Appendix S1)

Second, patterns among species might be obscured by those that occur within species, and multiple variables might interact. Although latitude as a single predictor is negatively associated with EPP rates (posterior mean latitude = −0.22, 95% CI [−0.39 to −0.06], *P*_mcmc_ = 0.01), the between species association was not significant after separating it from the significant within species effect (Table 2). Furthermore, the pattern between latitude and EPP within species varied with coloniality. Selecting just those socially monogamous species from our dataset which have been sampled in at least 10 different locations, indicates that EPP rates of each population correlated significantly with latitude for each of these four species (Figure 10, GLM, all: Deviance <−30, *df* = 1, *p* < .001). However, three of the four associations were negative with higher latitudes being associated with lower EPP (Figure 10a–c), thereby supporting the idea of a trade‐off between searching for extra‐pair matings and parental care (assuming that latitude covaries with variation in breeding synchrony). In contrast, a positive correlation was found for the colonial breeding species in this dataset (barn swallow, Figure 10d), supporting the idea that higher synchrony facilitates comparison among simultaneously fertile males (although this finding was not supported across all colonial socially monogamous species, Table 2). Thus, to gain a better understanding of which factors might explain interspecific variation in EPP, it is important to consider multiple variables simultaneously.

Third, rather than using arcsine transformations to normalize EPP data (e.g., Botero & Rubenstein, 2012; Spottiswoode & Møller, 2004), we analysed EPP using a binomial distribution and thereby account for variation in sample size. Median sample size was 131 individuals per species in our dataset, but variation was extensive with the best‐sampled species (great tit) having over 13,000 offspring sampled. Some estimates of EPP will thus be more reliable than others and by using a logistic regression the contribution of each study is weighed according to sample size. In addition to being unable to account for variation in sample size, arcsine transformations have been discouraged, as they can easily lead to biologically unrealistic or even impossible results especially if the variance is high and the data lie close to zero (Warton & Hui, 2011; Wilson & Hardy, 2002).

Overall, our results thus show that there is not much evidence that latitude (and thus breeding synchrony), life history or ecology can be a general explanation for interspecific variation in EPP in socially monogamous species, using the most comprehensive data available. An important caveat here is that the biased geographic sampling in the overall data, does compromise our ability to examine the interspecific effect of latitude, and this will be improved as further studies are conducted in tropical and subtropical lower latitude regions. Within species, the significant association between EPP and latitude corroborate the idea that breeding synchrony plays an important role in facilitating or constraining EPP (see Table 1). However, the above‐mentioned collinearity between latitude and other factors highlights the need for broadscale experimental work to confirm this, for example through the manipulation of breeding synchrony in populations at different latitudes.

## 6 ADAPTIVE AND NONADAPTIVE HYPOTHESES TO EXPLAIN VARIATION AND OCCURRENCE OF EPP

The question of why females mate with extra‐pair mates receives ongoing attention and over the past 30 years a large number of adaptive and nonadaptive hypotheses have been proposed (for overview see Table 1). The ‘good genes’ hypothesis, predicts that females will prefer males with higher quality genes than their social partner (e.g., Westneat et al., 1990), and therefore extra‐pair males should differ from the social partners that they are cuckolding, either phenotypically, or genetically (for genetic compatibility), with corresponding differences also observed in offspring. The potential difference in extra‐pair and within‐pair from the perspective of either sires and/or offspring has remained a focus of particular interest.

In their meta‐analysis Hsu et al. (2015), found no evidence for differences between either sires or offspring with respect to body size, sexually selected ornaments, age or genetic similarity. Although based on a similar sample size, a separate meta‐analysis found support for a correlation between pair genetic similarity and EPP across species (Arct et al., 2015) and this resulted in discussion about the generality of this finding. For example, it was pointed out that the 33 species that were the focus of that study are only a small fraction of all bird species and positive publication bias is likely to have resulted in negative or null results being underrepresented in the literature (Forstmeier, 2015). In addition, similar patterns might occur as the result of genetic structuring of populations, and the distribution of paternity among local and nonlocal males (Griffith, 2015), or strong inbreeding depression early in life (Reid, 2015). Finally, as pointed out by Nakagawa et al.. (2015), the heterogeneity of the effect sizes in the study by Arct et al. (2015) was large. Thus, while inbreeding avoidance could be a possible explanation for EPP in some species, it might not be a general explanation for all species. For example, genetic benefits might be particularly important in situations with strong genetic structuring like cooperative breeders with limited dispersal (Lichtenauer et al., 2019), or in contact zones between species where females in heterospecific pairings could use EPP to produce pure offspring (Slagsvold, Hansen, Johannessen, & Lifjeld, 2002; Veen et al., 2001). Similarly, it has been shown that genetic benefits might be more important for some individuals than for others within the same species, for example only individuals paired to a close relative or a male with low genetic compatibility may benefit from EPP (Brouwer et al., 2010; Hajduk et al., 2018). In summary, although there is some evidence for genetic benefits of EPP, for some individuals within some species and populations, it is unlikely to be a general explanation for the occurrence and variation in EPP either across, or within species.

Rather than an adaptive female choice, EPP has also been suggested to be a byproduct of selection on other characteristics of the mating system (Arnqvist & Kirkpatrick, 2005; Forstmeier et al., 2014). Such a mechanism can explain apparently nonadaptive female extra‐pair mating behaviour, and can for example be the result of a genetic correlation between male and female extra‐pair copulation behaviour which was found in a captive population of zebra finches (*Taeniopygiaguttata*, Forstmeier et al., 2011). However, such correlations have thus far not been detected in wild populations (Reid & Wolak, 2018), although they have not been the focus of much research. Thus, whether this means that apparently nonadaptive extra‐pair mating behaviour by females can actually evolve remains unclear. Although recent theoretical work suggests that this might be true when the behaviour is selectively neutral or only slightly maladaptive (Lyu, Servedio, & Sun, 2018).

However, it is important to note that seemingly nonadaptive behaviour might in fact be adaptive, but that the benefits of EPP for females are just hard to detect. One long‐standing explanation for EPP from the female's perspective is the idea that EPP helps her to gain insurance against infertility of her social male (fertility insurance hypothesis; Sheldon, 1994; Wetton & Parkin, 1991). Selection for such behaviour is predicted to be strong, given that incubating infertile eggs is likely to be very costly, especially when the opportunity to rebreed is lost (i.e., in temporally constrained highly seasonal breeders). However, testing the fertility insurance hypothesis using observational data is difficult, since it is impossible to know whether an egg sired by an extra‐pair male would otherwise have been infertile due to lack of functional sperm from the social male (Griffith, 2007). Possibly, the benefits females gain from insurance against infertility are much more general than typically considered, outweighing the direct costs that are usually invoked for females engaging in extra‐pair copulations.

In conclusion, there is considerable variation in the rate of EPP among avian species, and populations, and our results further confirm the difficulty of finding a general explanation underlying this variation (and indeed there is no reason to expect a general explanation, also see: Forstmeier et al., 2014). We have confirmed that there is a relatively strong phylogenetic signal to this variation, but also within a single species variation can often still be quite extensive. Although latitude was significantly negatively associated with EPP within noncolonial species, none of the broadscale ecological drivers was a good predictor for the level of interspecific variation in EPP in socially monogamous species. This result contrasts several previous studies which did not take heterogeneity within species of both the level of EPP and the predictors of interest into account. Despite the absence of broad‐scale drivers of the patterns of interspecific variation in EPP, we believe that a large part of the variation can still be explained, if approached from the right perspective. Direct benefits for females might have been underestimated, particularly because of the difficulty in testing the fertility insurance hypothesis. Fertility benefits could be a general driver of female extra‐pair behaviour, and could be maintained at a relatively constant level across all species, with deviations resulting from variation in costs and benefits of EPP. A major issue here is that we still have very little information about the extent to which extra‐pair copulations relate to extra‐pair fertilisations. Perhaps emerging animal tracking technology will allow for a better determination of the extent to which females engage in extra‐pair forays. New work in the area of female cryptic choice may also provide important insight into the level of multiple mating vs. extra‐pair fertilisations (see Box 2). The value of extra‐pair copulations to assure the fertility of their eggs, may always be important, but their incidence may sometimes be limited because the behaviour is directly or indirectly costly (e.g., opportunity costs). These costs might be reduced in cooperative breeders, where the relatively high EPP levels could result from the presence of helpers, providing an extra workforce to offset the cost of partner retaliation. The extreme variation among well sampled families in the level of EPP, suggests that certain behaviours and ecology of the species might facilitate or constrain EPP (perhaps through these costs). For example, opportunities for engaging in extra‐pair copulations might be increased when males are unable to control their female's behaviour due to the pattern of foraging, habitat characteristics, or trade‐offs with territory defence or paternal care, and further work on female behaviour will add valuable insight here (see Box 2).

BOX 2 **Future directions**

Target species from families in which genetic polyandry has not been investigated (see Figure 5).

Target populations from regions and biomes that have been relatively under‐represented in the studies to date (see Figure 6).

Focus more attention on behavioural polyandry. How many females, and to what extent do they engage in extra‐pair copulations? What is the nature of the relationship between extra‐pair copulations and extra‐pair fertilisations? Are there contexts in which extra‐pair copulations are frequent but genetic polyandry is low or absent due to the mechanisms of female cryptic choice?

Conduct more experimental work in which predicted determinants of genetic polyandry are effectively manipulated (e.g., female quality, breeding synchrony, access to resources etc).

Explore determinants of EPP using multiple populations of a single species, or genera where the species/populations chosen vary maximally in the parameter of focus (e.g., breeding synchrony, habitat structure, genetic structure).

We have also identified clear biases in the representation of both different parts of the avian tree of diversity, and with respect to global regions. We hope that these biases are addressed as further studies are planned and conducted (see Box 2), and that new data help to provide new insight into a complex and interesting phenomena at the nexus of avian behaviour, ecology and reproductive physiology. Genetic polyandry will remain an important component in our attempt to understand sexual selection, and the basic behavioural ecology of avian species, as well as our attempts to understand population genetic dynamics, and structuring.

## AUTHOR CONTRIBUTIONS

Both authors designed the study and extracted the data. L.B. analysed the data and wrote the paper with the help of S.C.G.

## Data Availability

Data and R script supporting this review is contained in Tables [Link], [Link], [Link], [Link], [Link] and Appendices [Link], [Link], [Link].

## References

[mec15259-bib-0001] Akçay, E. , & Roughgarden, J. (2007). Extra‐pair paternity in birds: Review of the genetic benefits. Evolutionary Ecology Research, 9, 855–868.

[mec15259-bib-0002] Arct, A. , Drobniak, S. M. , & Cichoń, M. (2015). Genetic similarity between mates predicts extrapair paternity – A meta‐analysis of bird studies. Behavioral Ecology, 26(4), 959–968. 10.1093/beheco/arv004

[mec15259-bib-0003] Arnqvist, G. , & Kirkpatrick, M. (2005). The evolution of infidelity in socially monogamous passerines: The strength of direct and indirect selection on extrapair copulation behavior in females. The American Naturalist, 165(5), S26–S37. 10.1086/429350 15795859

[mec15259-bib-0004] Biagolini, C. , Westneat, D. F. , & Francisco, M. R. (2017). Does habitat structural complexity influence the frequency of extra‐pair paternity in birds? Behavioral Ecology and Sociobiology, 71(7), 101 10.1007/s00265-017-2329-x

[mec15259-bib-0005] Birkhead, T. R. , & Biggins, J. D. (1987). Reproductive synchrony and extra‐pair copulations in birds. Ethology, 74, 320–334.

[mec15259-bib-0006] Birkhead, T. R. , & Møller, A. P. (1992). Sperm competition in birds. Evolutionary causes and consequences. London, UK: Academic Press.

[mec15259-bib-0007] Birkhead, T. R. , & Møller, A. P. (1996). Monogamy and sperm competition in birds In Black J. M. (Ed.), Partnerships in birds – The study of monogamy (pp. 324–343). Oxford, UK: Oxford University Press.

[mec15259-bib-0008] Bonier, F. , Eikenaar, C. , Martin, P. R. , & Moore, I. T. (2014). Extrapair paternity rates vary with latitude and elevation in Emberizid sparrows. The American Naturalist, 183(1), 54–61. 10.1086/674130 24334735

[mec15259-bib-0009] Boomsma, J. J. (2007). Kin selection versus sexual selection: Why the ends do not meet. Current Biology, 17(16), R673–R683. 10.1016/j.cub.2007.06.033 17714661

[mec15259-bib-0010] Botero, C. A. , & Rubenstein, D. R. (2012). Fluctuating environments, sexual selection and the evolution of flexible mate choice in birds. PLoS ONE, 7(2), e32311 10.1371/journal.pone.0032311 22359681 PMC3281128

[mec15259-bib-0011] Brouwer, L. , Barr, I. , van de Pol, M. , Burke, T. , Komdeur, J. , & Richardson, D. S. (2010). MHC‐dependent survival in a wild population: Evidence for hidden genetic benefits gained through extra‐pair fertilizations. Molecular Ecology, 19(16), 3444–3455. 10.1111/j.1365-294X.2010.04750.x 20670363

[mec15259-bib-0012] Brouwer, L. , van de Pol, M. , Aranzamendi, N. H. , Bain, G. , Baldassarre, D. T. , Brooker, L. C. , … Cockburn, A. (2017). Multiple hypotheses explain variation in extra‐pair paternity at different levels in a single bird family. Molecular Ecology, 26(23), 6717–6729. 10.1111/mec.14385 29068511

[mec15259-bib-0013] Burke, T. , & Bruford, M. W. (1987). DNA fingerprinting in birds. Nature, 327(6118), 149–152. 10.1038/327149a0 3574475

[mec15259-bib-0014] Burke, T. , Davies, N. B. , Bruford, M. W. , & Hatchwell, B. J. (1989). Parental care and mating behaviour of polyandrous dunnocks Prunellamodular is related to paternity by DNA fingerprinting. Nature, 338, 249–251. 10.1038/338249a0

[mec15259-bib-0015] Cardillo, M. (2002). The life‐history basis of latitudinal diversity gradients: How do species traits vary from the poles to the equator? Journal of Animal Ecology, 71(1), 79–87. 10.1046/j.0021-8790.2001.00577.x

[mec15259-bib-0016] Cockburn, A. (1998). Evolution of helping behavior in cooperatively breeding birds. Annual Review of Ecology and Systematics, 29, 141–177. 10.1146/annurev.ecolsys.29.1.141

[mec15259-bib-0017] Cockburn, A. (2006). Prevalence of different modes of parental care in birds. Proceedings of the Royal Society B: Biological Sciences, 273(1592), 1375–1383. 10.1098/rspb.2005.3458 PMC1560291 16777726

[mec15259-bib-0018] Cockburn, A. , & Double, M. (2008). Cooperatively breeding superb fairy‐wrens show no facultative manipulation of offspring sex ratio despite plausible benefits. Behavioral Ecology and Sociobiology, 62, 681–688. 10.1007/s00265-007-0492-1

[mec15259-bib-0019] Colwell, M. A. , & Oring, L. W. (1989). Extra‐pair mating in the spotted sandpiper: A female mate acquisition tactic. Animal Behaviour, 38, 675–684. 10.1016/S0003-3472(89)80013-2

[mec15259-bib-0020] Cornwallis, C. K. , Botero, C. A. , Rubenstein, D. R. , Downing, P. A. , West, S. A. , & Griffin, A. S. (2017). Cooperation facilitates the colonization of harsh environments. Nature Ecology & Evolution, 1(3), 57 10.1038/s41559-016-0057 28812731

[mec15259-bib-0021] Cornwallis, C. K. , West, S. A. , Davis, K. E. , & Griffin, A. S. (2010). Promiscuity and the evolutionary transition to complex societies. Nature, 466, 969–974. 10.1038/nature09335 20725039

[mec15259-bib-0022] del Hoyo, J. , Elliott, A. , & Christie, D. (2010). Handbook of the birds of the world, vol. 15 Weavers to new world warblers. Barcelona, Spain: Lynx Edit.

[mec15259-bib-0023] Díaz‐Muñoz, S. L. , DuVal, E. H. , Krakauer, A. H. , & Lacey, E. A. (2014). Cooperating to compete: Altruism, sexual selection and causes of male reproductive cooperation. Animal Behaviour, 88, 67–78. 10.1016/j.anbehav.2013.11.008

[mec15259-bib-0024] Double, M. , & Cockburn, A. (2000). Pre‐dawn infidelity: Females control extra‐pair mating in superb fairy‐wrens. Proceedings of the Royal Society of London Series B‐Biological Sciences, 267(1442), 465–470. 10.1098/rspb.2000.1023 PMC1690561 10737403

[mec15259-bib-0025] Drobniak, S. M. , Arct, A. , & Cichoń, M. (2015). Extrapair paternity and genetic similarity—we are not quite there yet: a response to comments on Arct et al. Behavioral Ecology, 26(4), 973–974. 10.1093/beheco/arv098

[mec15259-bib-0026] Dunn, P. O. , & Whittingham, L. A. (2006). Search costs influence the spatial distribution, but not the level, of extra‐pair mating in tree swallows. Behavioral Ecology and Sociobiology, 61(3), 449–454. 10.1007/s00265-006-0272-3

[mec15259-bib-0027] Englert Duursma, D. , Gallagher, R. V. , & Griffith, S. C. (2017). Characterizing opportunistic breeding at a continental scale using all available sources of phenological data: An assessment of 337 species across the Australian continent. The Auk, 134(3), 509–519. 10.1642/AUK-16-243.1

[mec15259-bib-0028] Evans, M. L. , Woolfenden, B. E. , Friesen, L. , & Stutchbury, B. J. M. (2009). Variation in the extra‐pair mating systems of Acadian Flycatchers and Wood Thrushes in forest fragments in southern Ontario. Journal of Field Ornithology, 80(2), 146–153. 10.1111/j.1557-9263.2009.00216.x

[mec15259-bib-0029] Ewen, J. G. , Ciborowski, K. L. , Clarke, R. H. , Boulton, R. L. , & Clarke, M. F. (2008). Evidence of extra‐pair paternity in two socially monogamous Australian passerines: The Crescent Honeyeater and the Yellow‐faced Honeyeater. Emu – Austral Ornithology, 108(2), 133 10.1071/MU07040

[mec15259-bib-0030] Fick, S. E. , & Hijmans, R. J. (2017). worldclim 2: New 1‐km spatial resolution climate surfaces for global land areas. International Journal of Climatology, 37(12), 4302–4315. 10.1002/joc.5086

[mec15259-bib-0031] Flanagan, S. P. , & Jones, A. G. (2019). The future of parentage analysis: From microsatellites to SNPs and beyond. Molecular Ecology, 28(3), 544–567. 10.1111/mec.14988 30575167

[mec15259-bib-0032] Forstmeier, W. (2015). Caution is needed when 90% of all possible estimates remain unpublished: a comment on Arct et al. Behavioral Ecology, 26(4), 972–973. 10.1093/beheco/arv044

[mec15259-bib-0033] Forstmeier, W. , Martin, K. , Bolund, E. , Schielzeth, H. , & Kempenaers, B. (2011). Female extrapair mating behavior can evolve via indirect selection on males. Proceedings of the National Academy of Sciences of the United States of America, 108(26), 10608–10613. 10.1073/pnas.1103195108 21670288 PMC3127899

[mec15259-bib-0034] Forstmeier, W. , Nakagawa, S. , Griffith, S. C. , & Kempenaers, B. (2014). Female extra‐pair mating: Adaptation or genetic constraint? Trends in Ecology & Evolution, 29(8), 456–464. 10.1016/j.tree.2014.05.005 24909948

[mec15259-bib-0035] Gillman, L. N. , Wright, S. D. , Cusens, J. , McBride, P. D. , Malhi, Y. , & Whittaker, R. J. (2015). Latitude, productivity and species richness: Latitude and productivity. Global Ecology and Biogeography, 24(1), 107–117. 10.1111/geb.12245

[mec15259-bib-0036] Gowaty, P. A. (1996). Battles of the sexes and origins of monogamy In Black J. M. (Ed.), Partnerships in birds (pp. 21–52). Oxford, UK: Oxford University Press.

[mec15259-bib-0037] Gray, E. M. (1997). Female red‐winged blackbirds accrue material benefits from copulating with extra‐pair males. Animal Behaviour, 53, 625–639. 10.1006/anbe.1996.0336

[mec15259-bib-0038] Griffin, A. S. , Alonzo, S. H. , & Cornwallis, C. K. (2013). Why do cuckolded males provide paternal care? PLoS Biology, 11(3), e1001520 10.1371/journal.pbio.1001520 23555193 PMC3608547

[mec15259-bib-0039] Griffith, S. C. (2007). The evolution of infidelity in socially monogamous passerines: Neglected components of direct and indirect selection. The American Naturalist, 169(2), 274–281. 10.1086/510601 17211810

[mec15259-bib-0040] Griffith, S. C. (2010). The role of multiple mating and extra‐pair paternity in creating and reinforcing boundaries between species in birds. Emu – Austral Ornithology, 110(1), 1–9. 10.1071/MU09057

[mec15259-bib-0041] Griffith, S. (2015). Genetic similarity is broadly associated with genetic polyandry in birds: a comment on Arct et al. Behavioral Ecology, 26(4), 970–971. 10.1093/beheco/arv031

[mec15259-bib-0042] Griffith, S. C. , Owens, I. P. F. , & Thuman, K. A. (2002). Extra pair paternity in birds: A review of interspecific variation and adaptive function. Molecular Ecology, 11(11), 2195–2212. 10.1046/j.1365-294X.2002.01613.x 12406233

[mec15259-bib-0043] Hackett, S. J. , Kimball, R. T. , Reddy, S. , Bowie, R. C. K. , Braun, E. L. , Braun, M. J. , … Yuri, T. (2008). A phylogenomic study of birds reveals their evolutionary history. Science, 320(5884), 1763–1768. 10.1126/science.1157704 18583609

[mec15259-bib-0044] Hajduk, G. K. , Cockburn, A. , Margraf, N. , Osmond, H. L. , Walling, C. A. , & Kruuk, L. E. B. (2018). Inbreeding, inbreeding depression, and infidelity in a cooperatively breeding bird. Evolution, 72(7), 1500–1514. 10.1111/evo.13496 PMC6099473 29761484

[mec15259-bib-0045] Halliday, T. , & Arnold, S. J. (1987). Multiple mating by females: A perspective from quantitative genetics. Animal Behaviour, 35(3), 939–941. 10.1016/S0003-3472(87)80138-0

[mec15259-bib-0046] Hamilton, W. D. (1990). Mate choice near or far. The American Zoologist, 30(2), 341–352. 10.1093/icb/30.2.341

[mec15259-bib-0047] Harts, A. M. F. , Booksmythe, I. , & Jennions, M. D. (2016). Mate guarding and frequent copulation in birds: A meta‐analysis of their relationship to paternity and male phenotype. Evolution, 70(12), 2789–2808. 10.1111/evo.13081 27714783

[mec15259-bib-0048] Hawkins, B. A. , Porter, E. E. , & FelizolaDiniz‐Filho, J. A. (2003). Productivity and history as predictors of the latitudinal diversity gradient of terrestrial birds. Ecology, 84(6), 1608–1623. 10.1890/0012-9658(2003)084[1608:PAHAPO]2.0.CO;2

[mec15259-bib-0049] Hsu, Y.‐H. , Schroeder, J. , Winney, I. , Burke, T. , & Nakagawa, S. (2015). Are extra‐pair males different from cuckolded males? A case study and a meta‐analytic examination. Molecular Ecology, 24, 1558–1571. 10.1111/mec.13124 25706253

[mec15259-bib-0050] Hughes, J. M. , Mather, P. B. , Toon, A. , Ma, J. , Rowley, I. , & Russell, E. (2003). High levels of extra‐group paternity in a population of Australian magpies *Gymnorhina tibicen*: Evidence from microsatellite analysis. Molecular Ecology, 12(12), 3441–3450. 10.1046/j.1365-294X.2003.01997.x 14629358

[mec15259-bib-0051] Hutchinson, J. M. C. , & Griffith, S. C. (2007). Extra‐pair paternity in the Skylark Alaudaarvensis: Extra‐pair paternity in the Skylark. Ibis, 150(1), 90–97. 10.1111/j.1474-919X.2007.00744.x

[mec15259-bib-0052] Ims, R. A. (1990). The ecology and evolution of reproductive synchrony. Trends in Ecology & Evolution, 5(5), 135–140. 10.1016/0169-5347(90)90218-3 21232341

[mec15259-bib-0053] Jetz, W. , Thomas, G. H. , Joy, J. B. , Hartmann, K. , & Mooers, A. O. (2012). The global diversity of birds in space and time. Nature, 491(7424), 444–448. 10.1038/nature11631 23123857

[mec15259-bib-0054] Johnsen, A. , & Lifjeld, J. T. (2003). Ecological constraints on extra‐pair paternity in the bluethroat. Oecologia, 136, 476–483. 10.1007/s00442-003-1286-4 12783296

[mec15259-bib-0055] Kaiser, S. A. , Sillett, T. S. , Risk, B. B. , & Webster, M. S. (2015). Experimental food supplementation reveals habitat‐dependent male reproductive investment in a migratory bird. Proceedings of the Royal Society B: Biological Sciences, 282(1803), 20142523 10.1098/rspb.2014.2523 PMC4345442 25673677

[mec15259-bib-0056] Kaiser, S. A. , Taylor, S. A. , Chen, N. , Sillett, T. S. , Bondra, E. R. , & Webster, M. S. (2017). A comparative assessment of SNP and microsatellite markers for assigning parentage in a socially monogamous bird. Molecular Ecology Resources, 17(2), 183–193. 10.1111/1755-0998.12589 27488248

[mec15259-bib-0057] Kempenaers, B. , Congdon, B. , Boag, P. , & Robertson, R. J. (1999). Extrapair paternity and egg hatchability in tree swallows: Evidence for the genetic compatibility hypothesis? Behavioral Ecology, 10(3), 304–311. 10.1093/beheco/10.3.304

[mec15259-bib-0058] Langmore, N. E. , Adcock, G. J. , & Kilner, R. M. (2007). The spatial organization and mating system of Horsfield's bronze‐cuckoos. Chalcitesbasalis. Animal Behaviour, 74(3), 403–412. 10.1016/j.anbehav.2006.09.019

[mec15259-bib-0059] Letunic, I. , & Bork, P. (2016). Interactive tree of life (iTOL) v3: An online tool for the display and annotation of phylogenetic and other trees. Nucleic Acids Research, 44(W1), W242–W245. 10.1093/nar/gkw290 27095192 PMC4987883

[mec15259-bib-0060] Lichtenauer, W. , van de Pol, M. , Cockburn, A. , & Brouwer, L. (2019). Indirect fitness benefits through extra‐pair mating are large for an inbred minority, but cannot explain widespread infidelity among red‐winged fairy wrens. Evolution, 73(3), 467–480. 10.1111/evo.13684 30666623 PMC7172280

[mec15259-bib-0061] Lifjeld, J. T. , Kleven, O. , Jacobsen, F. , McGraw, K. J. , Safran, R. J. , & Robertson, R. J. (2011). Age before beauty? Relationships between fertilization success and age‐dependent ornaments in barn swallows. Behavioral Ecology and Sociobiology, 65(9), 1687–1697. 10.1007/s00265-011-1176-4 21949464 PMC3156913

[mec15259-bib-0062] Lifjeld, J. T. , Slagsvold, T. , & Ellegren, H. (1997). Experimental mate switching in pied flycatchers: Male copulatory access and fertilization success. Animal Behaviour, 53, 1225–1232. 10.1006/anbe.1996.0430 9236018

[mec15259-bib-0063] Low, M. (2005). Female resistance and male force: context and patterns of copulation in the New Zealand stitchbird Notiomystis cincta. Journal of Avian Biology, 36(5), 436–448. 10.1111/j.0908-8857.2005.03460.x

[mec15259-bib-0064] Lyu, N. , Servedio, M. R. , & Sun, Y.‐H. (2018). Nonadaptive female pursuit of extrapair copulations can evolve through hitchhiking. Ecology and Evolution, 8(7), 3685–3692. 10.1002/ece3.3915 29686849 PMC5901172

[mec15259-bib-0065] MacArthur, R. H. (1964). Environmental factors affecting bird species diversity. The American Naturalist, 98, 387–397. 10.1086/282334

[mec15259-bib-0066] Maldonado‐Chaparro, A. A. , Montiglio, P.‐O. , Forstmeier, W. , Kempenaers, B. , & Farine, D. R. (2018). Linking the fine‐scale social environment to mating decisions: A future direction for the study of extra‐pair paternity: Linking mating decisions to the social environment. Biological Reviews, 93(3), 1558–1577. 10.1111/brv.12408 29533010

[mec15259-bib-0067] Matysioková, B. , & Remeš, V. (2013). Faithful females receive more help: The extent of male parental care during incubation in relation to extra‐pair paternity in songbirds. Journal of Evolutionary Biology, 26(1), 155–162. 10.1111/jeb.12039 23176707

[mec15259-bib-0068] Mays, H. L. , & Ritchison, G. (2004). The effect of vegetation density on male mate guarding and extra‐territorial forays in the yellow‐breasted chat (*Icteria virens*). Naturwissenschaften, 91(4), 195–198. 10.1007/s00114-004-0510-3 15085279

[mec15259-bib-0069] Møller, A. P. (1988). Female choice selects for male sexual tail ornaments in the monogamous swallow. Nature, 332, 640–642. 10.1038/332640a0

[mec15259-bib-0070] Møller, A. P. , & Birkhead, T. R. (1994). The evolution of plumage brightness in birds is related to extrapair paternity. Evolution, 48(4), 1089–1100. 10.1111/j.1558-5646.1994.tb05296.x 28564455

[mec15259-bib-0071] Møller, A. P. , & Ninni, P. (1998). Sperm competition and sexual selection: A meta‐analysis of paternity studies of birds. Behavioral Ecology and Sociobiology, 43(6), 345–358. 10.1007/s002650050501

[mec15259-bib-0072] Mulder, R. A. , Dunn, P. O. , Cockburn, A. , Lazenbycohen, K. A. , & Howell, M. J. (1994). Helpers liberate female fairy‐wrens from constraints on extra‐pair mate choice. Proceedings of the Royal Society of London Series B: Biological Sciences, 255(1344), 223–229.

[mec15259-bib-0073] Muñoz, A. P. , Kéry, M. , Martins, P. V. , & Ferraz, G. (2018). Age effects on survival of Amazon forest birds and the latitudinal gradient in bird survival. The Auk, 135(2), 299–313. 10.1642/AUK-17-91.1

[mec15259-bib-0074] Nakagawa, S. , Schroeder, J. , & Burke, T. (2015). Sugar‐free extrapair mating: A comment on Arct et al. Behavioral Ecology, 26(4), 971–972. 10.1093/beheco/arv041

[mec15259-bib-0075] Petrie, M. , & Kempenaers, B. (1998). Extra‐pair paternity in birds: Explaining variation between species and populations. Trends in Ecology & Evolution, 13(2), 52–58. 10.1016/S0169-5347(97)01232-9 21238200

[mec15259-bib-0076] Poiani, A. , & Wilks, C. (2000). Sexually transmitted diseases: A possible cost of promiscuity in birds? The Auk, 117(4), 1061–1065. 10.2307/4089652

[mec15259-bib-0077] QGIS Development Team (2018). QGIS geographic information system. Open source geospatial foundation project. Retrieved from https://qgis.org.

[mec15259-bib-0078] Ramos, A. G. , Nunziata, S. O. , Lance, S. L. , Rodríguez, C. , Faircloth, B. C. , Gowaty, P. A. , & Drummond, H. (2014). Habitat structure and colony structure constrain extrapair paternity in a colonial bird. Animal Behaviour, 95, 121–127. 10.1016/j.anbehav.2014.07.003

[mec15259-bib-0079] Reid, J. (2015). What can we really say about relatedness and extrapair paternity: a comment on Arct et al. Behavioral Ecology, 26(4), 969–970. 10.1093/beheco/arv027

[mec15259-bib-0080] Reid, J. M. , Duthie, A. B. , Wolak, M. E. , & Arcese, P. (2015). Demographic mechanisms of inbreeding adjustment through extra‐pair reproduction. Journal of Animal Ecology, 84(4), 1029–1040. 10.1111/1365-2656.12340 25645743 PMC4670719

[mec15259-bib-0081] Reid, J. M. , & Wolak, M. E. (2018). Is there indirect selection on female extra‐pair reproduction through cross‐sex genetic correlations with male reproductive fitness? Evolution Letters, 2(3), 159–168. 10.1002/evl3.56 30283673 PMC6121835

[mec15259-bib-0082] Sheldon, B. C. (1994). Male phenotype, fertility, and the pursuit of extra‐pair copulations by female birds. Proceedings of the Royal Society of London Series B: Biological Sciences, 257(1348), 25–30.

[mec15259-bib-0083] Sherman, P. W. , & Morton, M. L. (1988). Extra‐pair fertilizations in mountain white‐crowned sparrows. Behavioral Ecology and Sociobiology, 22(6), 413–420. 10.1007/BF00294979

[mec15259-bib-0084] Slagsvold, T. , Hansen, B. T. , Johannessen, L. E. , & Lifjeld, J. T. (2002). Mate choice and imprinting in birds studied by cross‐fostering in the wild. Proceedings of the Royal Society of London Series B: Biological Sciences, 269(1499), 1449–1455. 10.1098/rspb.2002.2045 12137574 PMC1691058

[mec15259-bib-0085] Spottiswoode, C. , & Møller, A. P. (2004). Extrapair paternity, migration, and breeding synchrony in birds. Behavioral Ecology, 15(1), 41–57. 10.1093/beheco/arg100

[mec15259-bib-0086] Stoffel, M. A. , Nakagawa, S. , & Schielzeth, H. (2017). rptr: Repeatability estimation and variance decomposition by generalized linear mixed‐effects models. Methods in Ecology and Evolution, 8(11), 1639–1644. 10.1111/2041-210X.12797

[mec15259-bib-0087] Stutchbury, B. J. M. (1998). Breeding synchrony best explains variation in extra‐pair mating system among avian species. Behavioral Ecology and Sociobiology, 43(3), 221–222. 10.1007/s002650050485

[mec15259-bib-0088] Stutchbury, B. J. M. , & Morton, E. S. (1995). The effect of breeding synchrony on extra‐pair mating systems in songbirds. Behaviour, 132, 675–690. 10.1163/156853995X00081

[mec15259-bib-0089] Thornhill, R. , & Alcock, J. (1983). The evolution of insect mating systems. Cambridge, MA: Harvard University Press.

[mec15259-bib-0090] Thrasher, D. J. , Butcher, B. G. , Campagna, L. , Webster, M. S. , & Lovette, I. J. (2018). Double‐digest RAD sequencing outperforms microsatellite loci at assigning paternity and estimating relatedness: A proof of concept in a highly promiscuous bird. Molecular Ecology Resources, 18(5), 953–965. 10.1111/1755-0998.12771 29455472

[mec15259-bib-0091] Townsend, A. K. , Clark, A. B. , & McGowan, K. J. (2010). Direct benefits and genetic costs of extrapair paternity for female American crows (*Corvus brachyrhynchos*). The American Naturalist, 175(1), E1–E9. 10.1086/648553 19929635

[mec15259-bib-0092] Tregenza, T. , & Wedell, N. (2000). Genetic compatibility, mate choice and patterns of parentage: Invited review. Molecular Ecology, 9(8), 1013–1027. 10.1046/j.1365-294x.2000.00964.x 10964221

[mec15259-bib-0093] Trivers, R. L. (1972). Parental investment and sexual selection In Campbell B. (Ed.), Sexual selection and the descent of man (pp. 136–179). London, UK: Heinemann.

[mec15259-bib-0094] Tryjanowski, P. , Antczak, M. , & Hromada, M. (2007). More secluded places for extra‐pair copulations in the great grey shrike *Lanius excubitor* . Behaviour, 144(1), 23–31. 10.1163/156853907779947436

[mec15259-bib-0095] Tryjanowski, P. , & Hromada, M. (2005). Do males of the great grey shrike, *Lanius excubitor*, trade food for extrapair copulations? Animal Behaviour, 69(3), 529–533. 10.1016/j.anbehav.2004.06.009

[mec15259-bib-0096] Valera, F. (2003). Male shrikes punish unfaithful females. Behavioral Ecology, 14(3), 403–408. 10.1093/beheco/14.3.403

[mec15259-bib-0097] van de Pol, M. , & Wright, J. (2009). A simple method for distinguishing within‐ versus between‐subject effects using mixed models. Animal Behaviour, 77(3), 753–758. 10.1016/j.anbehav.2008.11.006

[mec15259-bib-0098] Veen, T. , Borge, T. , Griffith, S. C. , Saetre, G. P. , Bures, S. , Gustafsson, L. , & Sheldon, B. C. (2001). Hybridization and adaptive mate choice in flycatchers. Nature, 411, 45–49. 10.1038/35075000 11333971

[mec15259-bib-0099] Ward, M. P. , Alessi, M. , Benson, T. J. , & Chiavacci, S. J. (2014). The active nightlife of diurnal birds: Extraterritorial forays and nocturnal activity patterns. Animal Behaviour, 88, 175–184. 10.1016/j.anbehav.2013.11.024

[mec15259-bib-0100] Warrington, M. H. , Rollins, L. A. , Russell, A. F. , & Griffith, S. C. (2015). Sequential polyandry through divorce and re‐pairing in a cooperatively breeding bird reduces helper‐offspring relatedness. Behavioral Ecology and Sociobiology, 69(8), 1311–1321. 10.1007/s00265-015-1944-7

[mec15259-bib-0101] Warton, D. I. , & Hui, F. K. C. (2011). The arcsine is asinine: The analysis of proportions in ecology. Ecology, 92(1), 3–10. 10.1890/10-0340.1 21560670

[mec15259-bib-0102] Weinman, L. R. , Solomon, J. W. , & Rubenstein, D. R. (2015). A comparison of single nucleotide polymorphism and microsatellite markers for analysis of parentage and kinship in a cooperatively breeding bird. Molecular Ecology Resources, 15(3), 502–511. 10.1111/1755-0998.12330 25224810

[mec15259-bib-0103] Westneat, D. F. (1994). To guard mates or go forage – Conflicting demands affect the paternity of male red‐winged blackbirds. The American Naturalist, 144(2), 343–354. 10.1086/285679

[mec15259-bib-0104] Westneat, D. F. , & Mays, H. L. (2005). Tests of spatial and temporal factors influencing extra‐pair paternity in red‐winged blackbirds: Factors affecting EPP. Molecular Ecology, 14(7), 2155–2167. 10.1111/j.1365-294X.2005.02562.x 15910334

[mec15259-bib-0105] Westneat, D. F. , Sherman, P. W. , & Morton, M. L. (1990). The ecology and evolution of extra‐pair copulations in birds In Current ornithology (vol. 7, pp. 331–369). New York, NY: Plenum Press.

[mec15259-bib-0106] Westneat, D. F. , & Stewart, I. R. K. (2003). Extra‐pair paternity in birds: Causes, correlates, and conflict. Annual Review of Ecology Evolution and Systematics, 34, 365–396. 10.1146/annurev.ecolsys.34.011802.132439

[mec15259-bib-0107] Wetton, J. H. , & Parkin, D. T. (1991). An association between fertility and cuckoldry in the House Sparrow, *Passer domesticus* . Proceedings of the Royal Society of London. Series B: Biological Sciences, 245, 227–233.

[mec15259-bib-0108] Williams, G. C. (1975). Sex and evolution. Princeton, NJ: Princeton University Press.

[mec15259-bib-0109] Wilson, K. , & Hardy, I. C. W. (2002). Statistical analysis of sex ratios: An introduction In Hardy I. C. W. (Ed.), Sex ratios (pp. 48–92). Cambridge, UK: Cambridge University Press.

[mec15259-bib-0110] Wink, M. , & Dyrcz, A. J. (1999). Mating systems in birds: A review of molecular studies. Acta Ornithologica, 34, 91–109.

[mec15259-bib-0111] Wolf, L. L. (1975). “Prostitution” behavior in a tropical hummingbird. Condor, 77, 140–144. 10.2307/1365783

