# Supplementary material for: Extra‐pair paternity in birds
Source: Mol Ecol. 2019 Oct 31;28(22):4864–82. doi: 10.1111/mec.15259 (PMC6899757; doi:10.1111/mec.15259)
Supplement: Supplementary file 1 [file MEC-28-4864-s001.docx]

**Supplemental Information for:**

**Extra-pair paternity in birds**

Lyanne Brouwer^1,2,3*^ & Simon C. Griffith^4^

^1^Department of Animal Ecology & Physiology, Institute for Water and Wetland Research, Radboud University, Nijmegen, The Netherlands

^2^Department of Animal Ecology, Netherlands Institute of Ecology (NIOO-KNAW), Wageningen, The Netherlands

^3^Division of Ecology and Evolution, Research School of Biology, The Australian National University, Canberra ACT 2601, Australia

^4^Department of Biological Sciences, Macquarie University, North Ryde, NSW 2109, Australia

**Table S1**. Overview of the studies on socially monogamous bird species with biparental care reporting 434 rates of the percentage of extra-pair paternity (EPP) for offspring and/or for the percentage of broods (EPbr) with at least one extra-pair offspring. N= number of offspring sampled, Nbr= number of broods sampled, Lat=latitude, Long=longitude. Habitat for passerines was extracted from the handbook of the Birds of the World (1) and was classified as: 1=woodland, 2=savanna, 3=scattered trees, 4=grassland, 5=cliffs, 6=scrub, 7=extensive undergrowth, 8=forest, 9= hedgerows, agricultural, gardens, vineyards etc., 10= heaths and moorland, 11=reed bed, 12=marshes, saltmarsh, 13=rainforest, 14=mountain steppes, 15=desert. The remarks column indicates studies which were conducted over a large geographic range, intra- or conspecific brood parasitism (IBP/CBP) was present, we report a subset of the data, we had difficulty in extracting numbers (and thus should be treated with care).

| Scientific name | Common name | N | EPP | Nbr | EPbr | Lat | Long | Habitat | Remarks | Ref |
| --- | --- | --- | --- | --- | --- | --- | --- | --- | --- | --- |
| *Acanthiza pusilla* | brown thornbill | 178 | 6.2 | 67 | 11.9 | -35.27 | 149.10 | 1,8,6,7,9 |  | (*2*) |
| *Accipiter cooperii* | Cooper’s hawk | 140 | 19.3 | 44 | 34.1 | 44.97 | -90.05 | - |  | (*3*) |
| *Acrocephalus arundinaceus* | great reed warbler | 194 | 9.8 | 48 | 10.4 | 49.67 | 10.85 | 11 |  | (*4*) |
| *Acrocephalus arundinaceus* | great reed warbler | 678 | 3.4 | - | - | 59.17 | 15.42 | 11 |  | (*5*) |
| *Acrocephalus arundinaceus* | great reed warbler | - | - | 272 | 6.6 | 59.17 | 15.42 | 11 |  | (*6*) |
| *Acrocephalus bistrigiceps* | black-browed reed-warbler | 140 | 6.4 | 37 | 13.5 | 36.87 | 139.05 | 11,12 |  | (*7*) |
| *Acrocephalus palustris* | marsh warbler | 131 | 3.1 | 33 | 9.1 | 50.37 | 10.75 | 6,7,11,12,9 |  | (*8*) |
| *Acrocephalus schoenobaenus* | sedge warbler | 201 | 7.5 | 44 | 22.7 | 59.17 | 15.42 | 12 |  | (*9*) |
| *Acrocephalus schoenobaenus* | sedge warbler | 403 | 8.4 | 85 | 18.8 | 51.45 | -0.55 | 12 |  | (*10*) |
| *Acrocephalus scirpaceus* | Eurasian reed-warbler | 186 | 6.5 | 52 | 15.4 | 52.30 | 0.30 | 11,12 |  | (*11*) |
| *Actitis hypoleucos* | common sandpiper | 83 | 15.7 | 27 | 18.5 | 54.75 | -3.02 | - |  | (*12*) |
| *Actitis hypoleucos* | common sandpiper | 53 | 7.5 | 15 | 20.0 | 57.78 | 12.32 | - |  | (*13*) |
| *Aegolius funereus* | boreal owl | 109 | 0.0 | 32 | 0.0 | 37.8─  64.9 | -107.8─  -147.7 | - | Sampled over large geographic range | (*14*) |
| *Agelaius phoeniceus* | red-winged blackbird | 125 | 25.6 | 40 | 50.0 | 42.40 | -85.40 | 12,9 |  | (*15*) |
| *Agelaius phoeniceus* | red-winged blackbird | 87 | 26.4 | 27 | 48.1 | 41.55 | -80.18 | 12,9 |  | (*15*) |
| *Agelaius phoeniceus* | red-winged blackbird | 56 | 28.6 | 20 | 50.0 | 26.53 | -78.53 | 12,9 |  | (*15*) |
| *Agelaius phoeniceus* | red-winged blackbird | 235 | 24.7 | 68 | 41.2 | 42.50 | -76.47 | 12,9 |  | (*16*) |
| *Agelaius phoeniceus* | red-winged blackbird | 617 | 25.6 | 203 | - | 44.52 | -76.37 | 12,9 |  | (*17*) |
| *Agelaius phoeniceus* | red-winged blackbird | 403 | 33.7 | 134 | 53.7 | 46.88 | -119.25 | 12,9 |  | (*18*) |
| *Agelaius xanthomus* | yellow-shouldered blackbird | 87 | 23.0 | 30 | 36.7 | 17.93 | -67.10 | 1,6,8 |  | (*19*) |
| *Alauda arvensis* | skylark | 171 | 20.5 | 52 | 26.9 | 51.17 | -4.63 | 10,4,12,9 |  | (*20*) |
| *Alle alle* | little auk | 26 | 0.0 | 26 | 0.0 | 77.00 | 15.37 | - |  | (*21*) |
| *Alle alle* | little auk | 62 | 3.2 | 64 | 3.1 | 77.00 | 15.55 | - |  | (*22*) |
| *Ammodramus maritimus* | seaside sparrow | 47 | 10.6 | 18 | 16.7 | 32.77 | -79.98 | 12 |  | (*23*) |
| *Anas platyrhynchos* | mallard | 204 | 9.3 | 25 | 48.0 | 49.15 | 14.72 | - | Also 10% offspring were CBP | (*24*) |
| *Anas strepera* | gadwalls | 261 | 4.2 | 29 | 27.6 | 48.62 | -99.08 | - |  | (*25*) |
| *Anthus spinoletta* | water pipit | 1052 | 5.2 | 258 | 12.4 | 46.78 | 9.85 | 4,14 |  | (*26*) |
| *Apus apus* | common swift | 88 | 4.5 | 42 | 9.5 | 51.75 | -1.25 | - |  | (*27*) |
| *Aquila heliaca* | Eastern imperial eagle | 166 | 0.0 | 86 | 0.0 | 53.48 | 63.62 | - |  | (*28*) |
| *Ardea alba* | great egret | 50 | 0.0 | 25 | 0.0 | -16.43─  -30.27 | -51.38─  -56.58 | - | Sibship analyses, sampled over large geographic range | (*29*) |
| *Asio otus* | long-eared owl | 59 | 0.0 | 12 | 0.0 | 46.88 | -114.12 | - |  | (*30*) |
| *Athene cunicularia* | burrowing owl | 121 | 1.7 | 68 | 1.5 | -38.72 | -62.27 | - | Also IBP | (*31*) |
| *Athene noctua* | little owl | 53 | 0.0 | 16 | 0.0 | 51.27 | 6.37 | - |  | (*32*) |
| *Baeolophus bicolor* | tufted titmouse | 34 | 8.8 | 9 | 22.2 | 40.00 | -82.88 | 1,9 |  | (*33*) |
| *Bartramia longicauda* | upland sandpiper | 177 | 18.1 | 56 | 30.0 | 39.08 | -96.58 | - |  | (*34*) |
| *Branta bernicla* | black brant | 374 | 5.6 | - | - | 61.25 | -165.58 | - |  | (*35*) |
| *Branta canadensis* | Canada geese | - | - | 42 | 14.3 | 43.00 | -84.00 | - |  | (*36*) |
| *Branta leucopsis* | barnacle goose | 16 | 0.0 | 9 | 0.0 | 78.92 | 11.90 | - |  | (*37*) |
| *Branta leucopsis* | barnacle goose | 137 | 0.0 | 63 | 0.0 | 57.28 | 18.75 | - |  | (*38*) |
| *Buteo ridgwayi* | Ridgway’s hawk | 79 | 0.0 | 41 | 0.0 | 19.00 | -70.00 | - |  | (*39*) |
| *Buteo swainsoni* | Swainson’s hawk | 56 | 5.4 | 27 | 7.4 | 41.82 | -122.00 | - |  | (*40*) |
| *Calamospiza melanocorys* | lark bunting | 455 | 25.1 | 153 | 52.3 | 41.35 | -104.08 | 4,6,9 |  | (*41*, *42*) |
| *Calidris alba* | sanderling | 342 | 2.0 | 48 | 6.2 | 74.50 | -21.00 | - |  | (*43*) |
| *Calidris maritima* | purple sandpiper | 82 | 1.2 | 27 | 3.7 | 78.22 | 15.58 | - |  | (*44*) |
| *Calidris mauri* | western sandpiper | 61 | 6.6 | 25 | 8.0 | 64.33 | -164.93 | - |  | (*13*) |
| *Callipepla squamata* | scaled quail | - | - | 22 | 9.1 | 36.00 | -100.00 | - |  | (*45*) |
| *Calonectris diomedea* | Cory’s shearwater | 69 | 11.6 | 44 | 18.2 | 36.92 | -25.17 | - |  | (*46*) |
| *Calonectris diomedea* | Cory’s shearwater | 34 | 0.0 | 22 | 0.0 | 42.00 | 9.00 | - |  | (*47*) |
| *Calonectris diomedea* | Cory’s shearwater | 46 | 0.0 | 29 | 0.0 | 35.00 | 24.00 | - |  | (*48*) |
| *Cardinalis cardinalis* | Northern cardinal | 37 | 13.5 | 19 | 15.8 | 37.65 | -84.12 | 1,8,6 |  | (*49*) |
| *Carduelis cannabina* | Eurasian linnet | 106 | 3.8 | 22 | 9.1 | 56.50 | 10.83 | 3,9,10 |  | (*50*) |
| *Carduelis tristis* | American goldfinch | 70 | 14.3 | 15 | 26.7 | 43.53 | -80.23 | 1,9 |  | (*51*) |
| *Carpodacus erythrinus* | scarlet rosefinch | 496 | 16.1 | 116 | 26.7 | 48.82 | 13.93 | 1,8,6,11,9 |  | (*52*) |
| *Carpodacus mexicanus* | house finch | 212 | 9.0 | 59 | 15.3 | 46.85 | -114.02 | 1,6,9 |  | (*53*) |
| *Carpodacus mexicanus* | house finch | 119 | 8.4 | 35 | 14.3 | 42.27 | -83.73 | 1,6,9 |  | (*54*) |
| *Catharacta lonnbergi* | brown skua | 45 | 0.0 | 16 | 0.0 | -43.80 | -176.50 | - |  | (*55*) |
| *Catharacta maccormicki* | south polar skua | 14 | 7.1 | 13 | 7.7 | -77.52 | 167.77 | - |  | (*56*) |
| *Centropus phasianinus* | pheasant coucal | 59 | 18.6 | 21 | 47.6 | -12.43 | 131.00 | - | Cuckoo, but duetting and exclusive territoriality so they are monogamous | (*57*) |
| *Cepphus grylle* | black guillemot | 46 | 0.0 | 32 | 0.0 | 67.43 | 11.88 | - |  | (*58*) |
| *Cercomacra tyrannina* | dusky antbird | 15 | 0.0 | 9 | 0.0 | 9.12 | -79.70 | 7,8 |  | (*59*) |
| *Charadrius alexandrinus* | Kentish plover | 46 | 0.0 | 19 | 0.0 | 38.40 | -26.96 | - |  | (*60*) |
| *Charadrius alexandrinus* | Kentish plover | 120 | 0.8 | 63 | 1.6 | 15.90 | -23.98 | - |  | (*60*) |
| *Charadrius alexandrinus* | Kentish plover | 184 | 1.6 | 73 | 4.1 | 36.72 | 35.05 | - |  | (*60*) |
| *Charadrius alexandrinus* | Kentish plover | 19 | 0.0 | 12 | 0.0 | 16.80 | 41.88 | - |  | (*60*) |
| *Charadrius alexandrinus* | Kentish plover | 57 | 0.0 | 28 | 0.0 | 24.27 | 54.60 | - |  | (*60*) |
| *Charadrius falklandicus* | two-banded plover | 15 | 0.0 | 6 | 0.0 | -52.43 | -59.08 | - |  | (*60*) |
| *Charadrius hiaticula* | ringed plover | 50 | 0.0 | 18 | 0.0 | 57.13 | 12.21 | - |  | (*61*) |
| *Charadrius marginatus* | white-fronted plover | 17 | 0.0 | 10 | 0.0 | -24.05 | 43.73 | - |  | (*60*) |
| *Charadrius modestus* | rufous-chested dotterel | 14 | 0.0 | 8 | 0.0 | -52.43 | -59.08 | - |  | (*60*) |
| *Charadrius nivosus* | snowy plover | 201 | 0.0 | 93 | 0.0 | 20.00 | -100.00 | - |  | (*60*) |
| *Charadrius pecuarius* | Kittlitz’s plover | 18 | 0.0 | 15 | 0.0 | -22.03 | 43.65 | - |  | (*60*) |
| *Charadrius ruficapillus* | red-capped plover | 7 | 0.0 | 4 | 0.0 | -37.88 | 144.78 | - |  | (*60*) |
| *Charadrius semipalmatus* | semipalmated plover | 85 | 4.7 | 24 | 4.2 | 58.75 | -95.07 | - |  | (*62*) |
| *Charadrius thoracicus* | Madagascar plover | 20 | 0.0 | 12 | 0.0 | -20.00 | 46.00 | - |  | (*60*) |
| *Chen caerulescens* | lesser snow goose | 80 | 5.0 | 23 | 13.0 | 67.23 | -100.27 | - |  | (*63*) |
| *Chen rossii* | Ross’s goose | 83 | 2.4 | 24 | 8.3 | 67.23 | -100.27 | - |  | (*63*) |
| *Chlidonias hybrida* | whiskered tern | 37 | 8.1 | 17 | 11.8 | 51.73 | 18.63 | - | Difficult to extract sample sizes | (*64*) |
| *Chlidonias niger* | North American black tern | 28 | 0.0 | 11 | 0.0 | 43.45 | -88.92 | - |  | (*65*) |
| *Ciconia ciconia* | white stork | - | - | 145 | 26.9 | 44.00 | 5.00 | - | Sibship analyses | (*66*) |
| *Cinclus cinclus* | white-throated dipper | 185 | 1.6 | 40 | 5.0 | 60.38 | 10.52 | 1,8,10 |  | (*67*) |
| *Circus pygargus* | Montagu’s harrier | 32 | 3.1 | 10 | 10.0 | 52.00 | 22.00 | - | Sibship analyses | (*68*) |
| *Colaptes auratus* | northern flicker | 326 | 0.0 | 46 | 0.0 | 51.87 | -122.03 | - |  | (*69*) |
| *Colinus virginianus* | Northern bobwhite | - | - | 34 | 85.3 | 36.00 | -100.00 | - |  | (*45*) |
| *Coracias garrulus* | European roller | 169 | 5.3 | 49 | 4.1 | 37.30 | -3.18 | - | Difficcult to extract sample sizes | (*70*) |
| *Coragyps atratus* | black vulture | 36 | 0.0 | 16 | 0.0 | 35.75 | -78.92 | - |  | (*71*) |
| *Corvus monedula* | Eurasian jackdaw | 39 | 2.6 | 15 | 6.7 | 50.92 | 11.57 | 1,3,10,9 |  | (*72*) |
| *Corvus monedula* | Eurasian jackdaw | 74 | 0.0 | 20 | 0.0 | 52.63 | -1.13 | 1,3,10,9 |  | (*73*) |
| *Cyanocitta stelleri* | Steller’s jay | 79 | 15.2 | 41 | 14.6 | 40.98 | -124.00 | 1,8,9 |  | (*74*) |
| *Cyanoliseus patagonus* | burrowing parakeet | 166 | 0.0 | 49 | 0.0 | -41.05 | -62.80 | - |  | (*75*) |
| *Cygnus atratus* | black swan | 332 | 15.1 | 85 | 37.6 | -37.55 | 14.82 | - |  | (*76*) |
| *Delichon urbicum* | house martin | 135 | 18.5 | 39 | 33.3 | 56.10 | -3.93 | 3,9,16 |  | (*77*) |
| *Delichon urbicum* | house martin | 73 | 19.2 | 20 | 35.0 | 61.42 | 8.87 | 3,9,16 |  | (*78*) |
| *Dendrocopos major* | great spotted woodpecker | 161 | 0.0 | 36 | 0.0 | 48.22 | 16.27 | - |  | (*79*) |
| *Dendrocopos medius* | middle spotted woodpecker | 61 | 0.0 | 13 | 0.0 | 48.22 | 16.27 | - |  | (*79*) |
| *Dendroica caerulescens* | black-throated blue warbler | 285 | 20.7 | 80 | 31.2 | 43.93 | -71.75 | 8 |  | (*80*) |
| *Dendroica caerulescens* | black-throated blue warbler | 125 | 27.2 | 38 | 44.7 | 43.93 | -71.75 | 8 |  | (*81*) |
| *Dendroica pensylvanica* | chestnut-sided warbler | 95 | 47.4 | 33 | 60.6 | 42.67 | -73.05 | 1,6,8 |  | (*82*) |
| *Dendroica petechia* | yellow warbler | 150 | 30.7 | 57 | 49.1 | 37.60 | -118.82 | 1,6,9 |  | (*83*) |
| *Dendroica petechia* | yellow warbler | 53 | 13.2 | 12 | 25.0 | 58.67 | -94.42 | 1,6,9 |  | (*84*) |
| *Dendroica petechia* | yellow warbler | 484 | 33.1 | 130 | 53.8 | 44.57 | -76.33 | 1,6,9 |  | (*85*) |
| *Diomedea exulans* | wandering albatross | 104 | 13.5 | 147 | 10.9 | -54.00 | -38.00 | - |  | (*86*) |
| *Diomedea exulans* | wandering albatross | 247 | 18.2 | 194 | 20.1 | -46.87 | 37.68 | - |  | (*87*) |
| *Diomedea exulans* | wandering albatross | 50 | 12.0 | 50 | 12.0 | -46.42 | 51.75 | - |  | (*88*) |
| *Diomedea exulans* | wandering albatross | 24 | 8.3 | 24 | 8.3 | -49.25 | 69.53 | - |  | (*88*) |
| *Dumetella carolinensis* | grey catbird | 455 | 13.0 | 165 | 24.8 | 39.00 | -76.00 | 7,9 |  | (*89*) |
| *Elaenia chiriquensis* | lesser elaenia | 38 | 36.8 | 15 | 66.7 | 9.12 | -79.68 | 1,3,9 |  | (*90*) |
| *Elaenia flavogaster* | yellow-bellied elaenia | 24 | 4.2 | 13 | 7.7 | 9.12 | -79.68 | 1,2,3,6 |  | (*90*) |
| *Emberiza citrinella* | yellowhammer | 123 | 37.4 | 32 | 68.8 | 59.90 | 17.53 | 1,6,10,9 |  | (*91*) |
| *Emberiza schoeniclus* | reed bunting | 216 | 54.6 | 58 | 86.2 | 52.13 | -1.13 | 12 |  | (*92*) |
| *Emberiza schoeniclus* | reed bunting | 473 | 51.0 | 121 | 73.6 | 51.75 | 4.75 | 12 |  | (*93*) |
| *Emberiza schoeniclus* | reed bunting | 835 | 37.7 | 213 | 62.9 | 46.90 | 6.93 | 12 |  | (*94*) |
| *Emberiza schoeniclus* | reed bunting | 669 | 37.1 | 181 | 56.4 | 47.00 | 8.00 | 12 |  | (*95*) |
| *Emberiza schoeniclus* | reed bunting | 332 | 29.5 | 72 | 54.2 | 61.42 | 8.87 | 12 |  | (*96*) |
| *Empidonax minimus* | least flycatcher | 86 | 34.9 | 23 | 56.5 | 44.57 | -76.32 | 6,8 |  | (*97*) |
| *Empidonax traillii* | willow flycatcher | 140 | 14.3 | 56 | 21.4 | 35.65 | -118.03 | 3,6 |  | (*98*) |
| *Empidonax virescens* | acadian flycatcher | 28 | 57.1 | 14 | 64.3 | 41.77 | -79.93 | 8 |  | (*99*) |
| *Empidonax virescens* | acadian flycatcher | 29 | 13.8 | 12 | 25.0 | 42.72 | -81.10 | 8 |  | (*100*) |
| *Empidonax virescens* | acadian flycatcher | 133 | 40.6 | 53 | 58.5 | 41.77 | -79.93 | 8 |  | (*101*) |
| *Erythrura gouldiae* | gouldian finch | 232 | 8.6 | 57 | 22.8 | -15.57 | 128.00 | 1 |  | (*102*) |
| *Eudromias morinellus* | Eurasian dotterel | 44 | 4.5 | 22 | 9.1 | 57.05 | -3.57 | - |  | (*103*) |
| *Eudyptes pachyrhynchus* | Fiordland penguin | 33 | 0.0 | 24 | 0.0 | -43.83 | 168.88 | - |  | (*104*) |
| *Eudyptes schlegeli* | royal penguin | 26 | 3.8 | 13 | 7.7 | -54.50 | 158.95 | - |  | (*105*) |
| *Euplectes orix* | red bishop | 115 | 39.1 | 47 | 63.8 | -33.43 | 25.75 | 4 |  | (*106*) |
| *Euplectes orix* | red bishop | 432 | 17.6 | 187 | 30.5 | -33.43 | 25.75 | 4 |  | (*107*) |
| *Falco columbarius* | merlin | 47 | 0.0 | 18 | 0.0 | 52.12 | -106.63 | - |  | (*108*) |
| *Falco eleonorae* | Eleonoras falcon | 60 | 0.0 | 17 | 0.0 | 38.00 | 24.98 | - |  | (*109*) |
| *Falco naumanni* | lesser kestrel | 24 | 4.2 | 8 | 12.5 | 37.17 | -6.35 | - |  | (*110*) |
| *Falco naumanni* | lesser kestrel | 72 | 8.3 | 23 | 8.7 | 41.35 | -0.18 | - |  | (*110*) |
| *Falco peregrinus* | peregrine falcon | 64 | 0.0 | 18 | 0.0 | - | - | - |  | (*111*) |
| *Falco sparverius* | American kestrel | 89 | 11.2 | 21 | 9.5 | 42.42 | -75.08 | - |  | (*112*) |
| *Falco tinnunculus* | Eurasian kestrel | 319 | 1.9 | 75 | 2.7 | 62.98 | 22.83 | - |  | (*113*) |
| *Ficedula albicollis* | collared flycatcher | 800 | 26.8 | 135 | 57.0 | 49.53 | 17.07 | 1,3,8,9 |  | (*114*) |
| *Ficedula albicollis* | collared flycatcher | 459 | 15.5 | 79 | 32.9 | 57.17 | 18.33 | 1,3,8,9 |  | (*115*) |
| *Ficedula albicollis* | collared flycatcher | 165 | 24.2 | 27 | 51.9 | 49.53 | 17.07 | 1,3,8,9 |  | (*116*) |
| *Ficedula albicollis* | collared flycatcher | - | - | 45 | 55.6 | 47.70 | 19.02 | 1,3,8,9 |  | (*117*) |
| *Ficedula albicollis* | collared flycatcher | - | - | 44 | 13.6 | 49.83─  57.17 | 17.25̶─  18.20 | 1,3,8,9 | Combined estimate for two populations | (*118*) |
| *Ficedula albicollis* | collared flycatcher | 482 | 15.6 | 78 | 34.6 | 50.10 | 20.40 | 1,3,8,9 |  | (*119*) |
| *Ficedula hypoleuca* | pied flycatcher | 268 | 13.1 | 59 | 28.8 | 40.80 | -4.02 | 1,8,9 |  | (*120*) |
| *Ficedula hypoleuca* | pied flycatcher | 884 | 5.1 | 164 | 12.8 | 52.45 | 7.25 | 1,8,9 |  | (*121*) |
| *Ficedula hypoleuca* | pied flycatcher | 135 | 4.4 | 27 | 14.8 | 59.98 | 10.63 | 1,8,9 |  | (*122*) |
| *Ficedula hypoleuca* | pied flycatcher | 165 | 5.5 | 31 | 12.9 | 52.53 | 7.35 | 1,8,9 |  | (*123*) |
| *Ficedula hypoleuca* | pied flycatcher | 1126 | 15.2 | 233 | 33.0 | 41.07 | -3.45 | 1,8,9 |  | (*124*) |
| *Ficedula hypoleuca* | pied flycatcher | 857 | 4.4 | 191 | 13.1 | 60.42 | 22.17 | 1,8,9 |  | (*125*) |
| *Ficedula hypoleuca* | pied flycatcher | 53 | 1.9 | 9 | 11.1 | 59.97 | 10.78 | 1,8,9 | Control dataset | (*126*) |
| *Ficedula hypoleuca* | pied flycatcher | 38 | 23.7 | 7 | 42.9 | 59.83 | 17.67 | 1,8,9 |  | (*127*) |
| *Ficedula hypoleuca* | pied flycatcher | 348 | 7.5 | 58 | 22.4 | 40.80 | -4.02 | 1,8,9 |  | (*128*) |
| *Ficedula hypoleuca* | pied flycatcher | 313 | 17.6 | 60 | 38.3 | 40.80 | -4.02 | 1,8,9 |  | (*129*) |
| *Ficedula hypoleuca* | pied flycatcher | 223 | 10.8 | 36 | 22.2 | 62.62 | 26.33 | 1,8,9 |  | (*130*) |
| *Ficedula hypoleuca* | pied flycatcher | - | - | 20 | 5.0 | 62.62 | 26.33 | 1,8,9 | Control dataset | (*131*) |
| *Ficedula hypoleuca* | pied flycatcher | 481 | 8.9 | 93 | 19.4 | 52.03 | 5.85 | 1,8,9 |  | (*132*) |
| *Ficedula parva* | red-breasted flycatcher | 159 | 7.5 | 33 | 24.2 | 52.68 | 23.87 | 1,8,9 |  | (*133*) |
| *Ficedula zanthopygia* | yellow rumped flycatcher | 325 | 22.2 | 64 | 54.7 | 45.00 | 126.00 | 8 |  | (*134*) |
| *Fratercula arctica* | Atlantic puffin | 38 | 0.0 | 38 | 0.0 | 67.43 | 11.88 | - |  | (*135*) |
| *Fregata minor* | great frigatebird | 92 | 1.1 | 92 | 1.1 | 23.75 | -166.02 | - |  | (*136*) |
| *Fregata minor* | great frigatebird | 46 | 8.7 | 46 | 8.7 | 23.75 | -166.02 | - |  | (*137*) |
| *Fringilla coelebs* | chaffinch | 47 | 17.0 | 13 | 23.1 | 53.33 | -1.53 | 1,8,9 |  | (*138*) |
| *Fulmarus glacialis* | Northern fulmar | 28 | 0.0 | 28 | 0.0 | 59.53 | -1.62 | - |  | (*139*) |
| *Gallinula chloropus* | moorhen | 68 | 0.0 | 13 | 0.0 | 52.63 | -0.27 | - |  | (*140*) |
| *Gavia immer* | common loon | 58 | 0.0 | 47 | 0.0 | 45.70 | -89.62 | - |  | (*141*) |
| *Geospiza fortis* | medium ground-finch | 1248 | 17.1 | - | - | -0.42 | -90.37 | 6,8 |  | (*142*) |
| *Geospiza scandens* | cactus finch | 368 | 10.3 | - | - | -0.42 | -90.37 | 15 |  | (*142*) |
| *Geothlypis trichas* | yellowthroat | 486 | 19.1 | 138 | 43.5 | 43.38 | -88.02 | 7,12 |  | (*143*) |
| *Grallina cyanoleuca* | Australian magpie-lark | 103 | 2.9 | 47 | 6.4 | -35.27 | 149.12 | 3,9 |  | (*144*) |
| *Grus canadensis* | sandhill crane | 45 | 11.1 | 18 | 22.2 | 43.60 | -89.60 | - |  | (*145*) |
| *Gyps fulvus* | griffon vulture | 30 | 0.0 | - | - | 44.27 | 5.27 | - |  | (*146*) |
| *Gyps fulvus* | griffon vulture | 10 | 0.0 | - | - | 43.77 | 6.37 | - |  | (*146*) |
| *Habia fuscicauda* | red-throated ant-tanager | 41 | 41.5 | 19 | 52.6 | 9.08 | -79.65 | 6,8 |  | (*147*) |
| *Haematopus ostralegus* | Eurasian oystercatcher | 65 | 1.5 | 26 | 3.8 | 53.48 | 6.22 | - |  | (*148*) |
| *Hirundo ariel* | fairy martin | 203 | 13.8 | 70 | 20.0 | -37.80 | 144.95 | 1,3,4 |  | (*149*) |
| *Hirundo rustica* | barn swallow | 214 | 28.5 | 52 | 51.9 | 45.43 | 8.62 | 3,4,9 |  | (*150*) |
| *Hirundo rustica* | barn swallow | 603 | 31.2 | 139 | - | 44.47 | -76.47 | 3,4,9 |  | (*151*) |
| *Hirundo rustica* | barn swallow | 917 | 28.8 | 210 | 48.1 | 44.57 | -76.32 | 3,4,9 |  | (*152*) |
| *Hirundo rustica* | barn swallow | 261 | 28.0 | 63 | 33.3 | 57.20 | 10.00 | 3,4,9 |  | (*153*) |
| *Hirundo rustica* | barn swallow | 108 | 23.1 | 25 | 60.0 | 49.07 | 14.73 | 3,4,9 |  | (*154*) |
| *Hirundo rustica* | barn swallow | 80 | 30.0 | 18 | 50.0 | 50.50 | 30.82 | 3,4,9 |  | (*154*, *155*) |
| *Hirundo rustica* | barn swallow | 424 | 34.2 | 94 | 55.3 | 37.22 | -80.42 | 3,4,9 |  | (*156*) |
| *Hirundo rustica* | barn swallow | 296 | 2.7 | 65 | 7.7 | 37.12 | 138.02 | 3,4,9 |  | (*157*) |
| *Hirundo rustica* | barn swallow | 143 | 8.4 | 33 | 15.2 | 35.22 | 139.88 | 3,4,9 |  | (*158*) |
| *Hirundo rustica* | barn swallow | 158 | 22.2 | 38 | 36.8 | 35.18 | 139.92 | 3,4,9 |  | (*158*) |
| *Hirundo rustica* | barn swallow | 674 | 17.8 | 170 | 32.4 | 38.83 | -6.98 | 3,4,9 |  | (*159*) |
| *Hirundo rustica* | barn swallow | - | - | 53 | 49.1 | 42.35 | -76.50 | 3,4,9 |  | (*160*) |
| *Hirundo rustica* | barn swallow | - | - | 11 | 45.5 | 44.55 | -76.32 | 3,4,9 |  | (*161*) |
| *Hirundo rustica* | barn swallow | 161 | 15.5 | 41 | 43.9 | 32.97 | 35.55 | 3,4,9 |  | (*162*) |
| *Hylocichla mustelina* | wood thrush | 151 | 6.0 | 51 | 13.7 | 41.77 | -79.93 | 1,8 |  | (*163*) |
| *Hylocichla mustelina* | wood thrush | 112 | 40.2 | 36 | 66.7 | 43.25 | -80.88 | 1,8 |  | (*100*) |
| *Hymenolaimus malacorhynchos* | blue duck | 14 | 0.0 | 10 | 0.0 | -39.38 | 175.13 | - |  | (*164*) |
| *Icteria virens* | yellow-breasted chat | 13 | 30.8 | 55 | 50.9 | 49.13─ 49.49 | -117.70─  -119.60 | 1,6 | Sibship analyses, sampled over large geographic range | (*165*) |
| *Icterus galbula* | Bullock´s oriole | 202 | 32.2 | 48 | 45.8 | 36.37 | -121.55 | 1,9 |  | (*166*) |
| *Jabiru mycteria* | jabiru | 34 | 2.9 | 13 | 7.7 | -17.00 | -56.00 | - |  | (*167*) |
| *Junco hyemalis* | dark-eyed junco | 229 | 26.2 | 89 | - | 32.87 | -117.02 | 1,8,9 | Population resulted from recent colonization | (*168*) |
| *Junco hyemalis* | dark-eyed junco | 124 | 36.3 | 40 | - | 32.87 | -116.03 | 1,8,9 |  | (*168*) |
| *Junco hyemalis* | dark-eyed junco | 2148 | 27.2 | - | - | 37.37 | -80.53 | 1,8,9 |  | (*169*) |
| *Junco hyemalis* | dark-eyed junco | - | - | 41 | 43.9 | 37.37 | -80.53 | 1,8,9 | Control dataset | (*170*) |
| *Jynx torquilla* | Eurasian wryneck | 292 | 0.7 | 50 | 2.0 | 51.90 | 11.00 | - |  | (*171*) |
| *Lagopus lagopus* | willow ptarmigan | 256 | 9.4 | 38 | 13.2 | 59.83 | -136.33 | - |  | (*172*) |
| *Lagopus leucura* | white-tailed ptarmigan | 58 | 5.2 | 18 | 16.7 | 48.68 | -113.72 | - |  | (*173*) |
| *Laniarius atrococcineus* | crimson-breasted shrike | 74 | 18.9 | 41 | 29.3 | -26.97 | 21.82 | 1,2,3 |  | (*174*) |
| *Lanius bucephalus* | bull-headed shrike | 99 | 10.1 | 24 | 16.7 | 34.57 | 135.53 | 8,6,9 |  | (*175*) |
| *Lanius ludovicianus* | loggerhead shrike | 179 | 4.5 | 36 | 13.9 | 34.00 | -98.00 | 9 |  | (*176*) |
| *Lanius minor* | lesser gray shrike | 136 | 0.0 | 36 | 0.0 | 48.58 | 19.30 | 1,3,9 |  | (*177*) |
| *Larus canus* | common gull | 55 | 3.6 | 24 | 8.3 | 51.67 | 21.58 | - |  | (*178*) |
| *Larus occidentalis* | western gull | 33 | 0.0 | 22 | 0.0 | 37.70 | -123.00 | - |  | (*179*) |
| *Larus ridibundus* | Black-headed gull | 79 | 20.3 | 30 | 33.3 | 48.92 | 17.08 | - |  | (*180*) |
| *Lichenostomus chrysops* | yellow-faced honeyeater | 18 | 44.4 | - | - | -37.68 | 145.52 | 1,3,8 |  | (*181*) |
| *Locustella luscinioides* | Savi’s warbler | 392 | 4.1 | 102 | 5.9 | 40.63 | -8.68 | 11,12 |  | (*182*) |
| *Loxia curvirostra* | common crossbill | 96 | 0.0 | 34 | 0.0 | 59.50 | 11.33 | 1,8 |  | (*183*) |
| *Loxioides bailleui* | palila | 20 | 0.0 | 12 | 0.0 | 19.82 | -155.47 | 8,3 |  | (*184*) |
| *Luscinia megarhynchos* | common nightingale | 121 | 21.5 | 28 | 46.4 | 52.40 | 12.97 | 1,3,9 |  | (*185*) |
| *Luscinia svecica* | bluethroat | 1568 | 26.3 | 245 | 47.8 | 62.42 | 8.87 | 6,7,3,11,12 | Control dataset | (*186*) |
| *Luscinia svecica* | bluethroat | 183 | 25.7 | 33 | 54.5 | 62.42 | 8.87 | 6,7,3,11,12 |  | (*187*) |
| *Luscinia svecica* | bluethroat | 162 | 42.0 | 36 | 63.9 | 27.33 | -2.42 | 6,7,3,11,12 |  | (*188*) |
| *Megascops asio* | eastern screech-owl | 80 | 0.0 | 23 | 0.0 | 37.62 | -84.12 | - |  | (*189*) |
| *Melospiza georgiana* | swamp sparrow | 149 | 20.8 | 59 | 44.1 | 39.60 | -79.30 | 1,12 |  | (*190*) |
| *Melospiza georgiana* | swamp sparrow | 201 | 20.9 | 54 | 38.9 | 39.30 | -75.50 | 1,12 |  | (*190*) |
| *Melospiza melodia* | song sparrow | 191 | 23.6 | 72 | 36.1 | 47.65 | -122.03 | 1,3 |  | (*191*) |
| *Melospiza melodia* | song sparrow | 38 | 10.5 | 10 | 30.0 | 44.75 | -63.68 | 1,3 |  | (*192*) |
| *Melospiza melodia* | song sparrow | 2667 | 28.2 | - | - | 48.63 | -123.02 | 1,3 |  | (*193*) |
| *Miliaria calandra* | corn bunting | 44 | 4.5 | 15 | 6.7 | 57.62 | -7.50 | 4,14,9,10 |  | (*194*) |
| *Mycteria americana* | wood stork | 58 | 0.0 | 29 | 0.0 | -16.70─  -1.93 | -50.23─  -57.50 | - | Sibship analyses, sampled over large geographic range | (*29*) |
| *Myiopsitta monachus* | monk parakeet | 58 | 0.0 | 52 | 0.0 | 27.00 | -81.00 | - |  | (*195*) |
| *Myiopsitta monachus* | monk parakeet | 27 | 0.0 | 14 | 0.0 | 41.00 | -72.00 | - |  | (*195*) |
| *Nectarinia osea* | orange-tufted sunbird | 88 | 22.7 | 47 | 36.2 | 32.08 | 34.78 | 1,2,4,8 |  | (*196*) |
| *Notiomystis cincta* | New Zealand hihi | 1538 | 68.0 | 485 | 88.9 | -36.60 | 174.88 | 8 | Difficult to extract sample sizes | (*197*) |
| *Notiomystis cincta* | New Zealand hihi | 188 | 47.3 | 61 | 82.0 | -38.08 | 176.28 | 8 |  | (*198*) |
| *Notiomystis cincta* | New Zealand hihi | 34 | 35.3 | 10 | 80.0 | -36.60 | 174.88 | 8 |  | (*199*) |
| *Oceanites oceanicus* | Wilson’s storm-petrel | 63 | 0.0 | 63 | 0.0 | -51.72 | -61.29 | - |  | (*200*) |
| *Oceanodroma leucorhoa* | Leach’s storm-petrel | 42 | 0.0 | 42 | 0.0 | 44.58 | -66.75 | - |  | (*201*) |
| *Oenanthe oenanthe* | northern wheatear | 73 | 11 | 17 | 29.4 | 52.77 | -4.78 | 3,10,14 |  | (*202*) |
| *Oenanthe oenanthe* | northern wheatear | 62 | 3.2 | 10 | 10.0 | 52.77 | -4.78 | 3,10,14 | Control dataset | (*203*) |
| *Oenanthe oenanthe* | northern wheatear | 132 | 25.8 | 28 | 46.4 | 49.50 | 8.17 | 3,10,14 |  | (*204*) |
| *Otus elegans* | elegant scops-owl | 200 | 0.5 | 108 | 0.9 | 22.00 | 121.08 | - |  | (*205*) |
| *Otus flammeolus* | flammulated owl | 37 | 0.0 | 17 | 0.0 | 34.50 | -108.00 | - |  | (*206*) |
| *Pachycephala pectoralis* | golden whistlers | 130 | 19.2 | 65 | 23.1 | -37.52 | 145.53 | 1,8,13 |  | (*207*) |
| *Pachyptila belcheri* | thin-billed prion | 34 | 20.6 | 34 | 20.6 | -51.72 | -61.28 | - |  | (*208*) |
| *Panurus biarmicus* | bearded tit | 187 | 14.4 | 44 | 29.5 | 47.93 | 16.75 | 11,12 |  | (*209*) |
| *Paradoxornis webbianus* | vinous-throated parrotbills | 246 | 7.7 | 50 | 26.0 | 37.53 | 127.03 | 6,11,12,9 |  | (*210*) |
| *Parus ater* | coal tit | 3559 | 31.4 | 483 | 70.8 | 52.45 | 7.25 | 1,8,9 |  | (*211*) |
| *Parus ater* | coal tit | 158 | 25.3 | 20 | 75.0 | 52.33 | 11.00 | 1,8,9 |  | (*212*) |
| *Parus atricapillus* | black-capped chickadee | 351 | 14.8 | 57 | 33.3 | 44.57 | -76.32 | 1,8 |  | (*213*) |
| *Parus atricapillus* | black-capped chickadee | 359 | 8.9 | 58 | 29.3 | 44.57 | -76.32 | 1,8 |  | (*214*) |
| *Parus caeruleus* | blue tit | 288 | 25.3 | 50 | 68.0 | 42.52 | -8.77 | 1,6,9 |  | (*215*) |
| *Parus caeruleus* | blue tit | 839 | 16.1 | 97 | 69.1 | 43.67 | 3.67 | 1,6,9 |  | (*216*) |
| *Parus caeruleus* | blue tit | 205 | 18.0 | 30 | 50.0 | 42.55 | 8.92 | 1,6,9 |  | (*216*) |
| *Parus caeruleus* | blue tit | - | - | 84 | 27.4 | 53.13 | 6.58 | 1,6,9 | Control dataset | (*217*) |
| *Parus caeruleus* | blue tit | 850 | 13.8 | 84 | 48.8 | 48.28 | 4.28 | 1,6,9 |  | (*218*) |
| *Parus caeruleus* | blue tit | 2452 | 15.7 | 248 | - | 48.22 | 16.33 | 1,6,9 |  | (*219*) |
| *Parus caeruleus* | blue tit | 51 | 5.9 | 7 | 28.6 | 59.83 | 17.63 | 1,6,9 |  | (*220*) |
| *Parus caeruleus* | blue tit | 88 | 6.8 | 14 | 28.6 | 59.93 | 10.55 | 1,6,9 | Control dataset | (*221*) |
| *Parus caeruleus* | blue tit | 1443 | 12.5 | 165 | 41.8 | 51.25 | 4.47 | 1,6,9 |  | (*222*) |
| *Parus caeruleus* | blue tit | 466 | 6.7 | 47 | 36.2 | 58.87 | 9.60 | 1,6,9 |  | (*223*) |
| *Parus caeruleus* | blue tit | 986 | 11.7 | 103 | 39.8 | 54.05 | -2.80 | 1,6,9 |  | (*224*) |
| *Parus caeruleus* | blue tit | 1176 | 12.8 | 122 | 46.7 | 53.13 | 6.58 | 1,6,9 |  | (*225*) |
| *Parus caeruleus* | blue tit | - | - | 396 | 46.0 | 48.13 | 10.88 | 1,6,9 |  | (*226*) |
| *Parus caeruleus* | blue tit | 635 | 11.3 | - | - | 48.13 | 10.88 | 1,6,9 |  | (*227*) |
| *Parus caeruleus* | blue tit | - | - | 561 | 57.0 | 48.22 | 16.33 | 1,6,9 |  | (*226*) |
| *Parus caeruleus* | blue tit | - | - | 36 | 55.6 | 53.13 | 6.58 | 1,6,9 | Control dataset | (*228*) |
| *Parus cristatus* | crested tit | 136 | 11.0 | 20 | 30.0 | 51.48 | 4.93 | 1,3 |  | (*229*) |
| *Parus major* | great tit | 6441 | 9.6 | - | - | 47.97 | 11.23 | 1,8,9 |  | (*230*) |
| *Parus major* | great tit | 445 | 6.3 | 47 | 31.9 | 47.08 | 17.83 | 1,8,9 |  | (*231*) |
| *Parus major* | great tit | 304 | 20.4 | 47 | 55.3 | 39.55 | -4.33 | 1,8,9 |  | (*232*) |
| *Parus major* | great tit | 47 | 14.9 | 10 | 50.0 | 59.83 | 17.67 | 1,8,9 |  | (*220*) |
| *Parus major* | great tit | 82 | 8.5 | 13 | 30.8 | 59.93 | 10.55 | 1,8,9 | Control dataset | (*221*) |
| *Parus major* | great tit | 229 | 16.6 | 32 | 53.1 | 33.52 | 130.03 | 1,8,9 |  | (*233*) |
| *Parus major* | great tit | 408 | 8.1 | 55 | 27.3 | 58.87 | 9.60 | 1,8,9 |  | (*223*) |
| *Parus major* | great tit | 2013 | 7.5 | 265 | 34.0 | 52.37 | 11.02 | 1,8,9 |  | (*234*) |
| *Parus major* | great tit | 192 | 9.9 | 23 | 39.1 | 55.67 | 12.57 | 1,8,9 |  | (*235*) |
| *Parus major* | great tit | 1185 | 12.7 | 164 | 48.8 | 51.77 | -1.13 | 1,8,9 |  | (*236*) |
| *Parus major* | great tit | 681 | 8.5 | 78 | 39.7 | 51.42 | 7.65 | 1,8,9 |  | (*237*) |
| *Parus major* | great tit | 667 | 6.4 | 99 | 25.3 | 52.00 | 5.83 | 1,8,9 |  | (*238*) |
| *Parus major* | great tit | 516 | 3.5 | 82 | 8.5 | 53.30 | 5.07 | 1,8,9 |  | (*239*) |
| *Parus major* | great tit | 34 | 2.9 | 4 | 25.0 | 52.15 | 10.32 | 1,8,9 |  | (*240*) |
| *Parus major* | great tit | 710 | 8.7 | 92 | 30.4 | 42.67 | 141.60 | 1,8,9 |  | (*241*) |
| *Parus montanus* | willow tit | 787 | 6.7 | 117 | 21.4 | 65.00 | 25.50 | 1,8 |  | (*242*) |
| *Parus montanus* | willow tit | 112 | 0.9 | 24 | 4.2 | 65.00 | 25.50 | 1,8 |  | (*243*) |
| *Parus teneriffae* | African blue tit | 137 | 15.3 | 31 | 38.7 | 28.42 | 16.38 | 1,6,9 |  | (*244*) |
| *Parus varius* | varied tit | 251 | 14.7 | 40 | 40.0 | 40.02 | 122.93 | 7,8 |  | (*245*) |
| *Passer domesticus* | house sparrow | 176 | 36.9 | 69 | 63.8 | 47.33 | -2.87 | 3,9 |  | (*246*) |
| *Passer domesticus* | house sparrow | 53 | 3.8 | 17 | 11.8 | 53.07 | -0.95 | 3,9 |  | (*247*) |
| *Passer domesticus* | house sparrow | 109 | 10.1 | 25 | 28.0 | 41.38 | 2.15 | 3,9 |  | (*247*) |
| *Passer domesticus* | house sparrow | 517 | 17.2 | 146 | - | 35.20 | -97.43 | 3,9 |  | (*248*) |
| *Passer domesticus* | house sparrow | 305 | 1.3 | 112 | 3.6 | 51.00 | -4.00 | 3,9 |  | (*249*) |
| *Passer domesticus* | house sparrow | 3939 | 17.5 | 1115 | 37.9 | 51.00 | -4.00 | 3,9 |  | (*250*) |
| *Passer domesticus* | house sparrow | 419 | 10.7 | 126 | 26.2 | 35.00 | -83.00 | 3,9 |  | (*251*) |
| *Passer domesticus* | house sparrow | 267 | 12.7 | 75 | 26.7 | 35.00 | -83.00 | 3,9 |  | (*252*) |
| *Passer domesticus* | house sparrow | 129 | 10.1 | 35 | 28.6 | 48.17 | 16.30 | 3,9 |  | (*253*) |
| *Passer domesticus* | house sparrow | - | - | 9 | 66.7 | 48.17 | 16.30 | 3,9 | Control dataset | (*254*) |
| *Passer domesticus* | house sparrow | 171 | 7.0 | 54 | 9.3 | 40.63 | -4.00 | 3,9 |  | (*255*) |
| *Passer domesticus* | house sparrow | 536 | 13.6 | 183 | 26.8 | 53.07 | -0.95 | 3,9 |  | (*256*) |
| *Passer domesticus* | house sparrow | 136 | 19.9 | 41 | 36.6 | 35.20 | -97.43 | 3,9 |  | (*257*) |
| *Passer montanus* | tree sparrow | 114 | 10.5 | 35 | 22.9 | 46.52 | 6.57 | 1,9 |  | (*258*) |
| *Passer montanus* | tree sparrow | 151 | 7.9 | 40 | 25.0 | 41.58 | -2.43 | 1,9 |  | (*258*) |
| *Passer montanus* | tree sparrow | 76 | 9.2 | 19 | 21.1 | 46.25 | 20.13 | 1,9 |  | (*259*) |
| *Passerculus sandwichensis* | savannah sparrow | 160 | 23.1 | 42 | 42.9 | 44.58 | -66.77 | 4,14,9 |  | (*260*) |
| *Passerculus sandwichensis* | savannah sparrow | 145 | 47.6 | 43 | 65.1 | 44.58 | -66.77 | 4,14,9 |  | (*261*) |
| *Passerculus sandwichensis* | savannah sparrow | 411 | 47.2 | 116 | 68.1 | 44.58 | -66.77 | 4,14,9 |  | (*262*) |
| *Passerculus sandwichensis* | savannah sparrow | 191 | 53.9 | 77 | 71.4 | 44.00 | -72.00 | 4,14,9 | Control dataset, difficult to extract sample sizes | (*263*) |
| *Passerina caerulea* | blue grosbeak | 55 | 52.7 | 20 | 70.0 | 32.60 | -85.30 | 8,9,6 |  | (*264*) |
| *Passerina cyanea* | indigo bunting | 63 | 34.9 | 25 | 48.0 | 35.88 | -79.02 | 1,9 |  | (*265*) |
| *Petroica australis* | New Zealand robin | 62 | 0.0 | 37 | 0.0 | -41.08 | 174.27 | 6,8,9 |  | (*266*) |
| *Petroica australis* | New Zealand robin | 198 | 0.5 | 54 | 1.9 | -45.00 | 168.00 | 6,8,9 |  | (*267*) |
| *Petroica goodenovii* | red-capped robin | 240 | 22.1 | - | 37.0 | -36.17 | 144.22 | 1,6 |  | (*268*) |
| *Petronia petronia* | rock sparrow | 277 | 24.5 | 56 | 44.6 | 44.97 | 6.63 | 14,15,9 |  | (*269*) |
| *Petronia petronia* | rock sparrow | 181 | 32.0 | 42 | 57.1 | - | - | 14,15,9 |  | (*270*) |
| *Phainopepla nitens* | phainopepla | 48 | 0.0 | 25 | 0.0 | 33.68 | -117.00 | 1,3,15 |  | (*271*) |
| *Phalacrocorax aristotelis* | shag | 161 | 9.3 | 87 | 12.6 | 56.18 | -2.57 | - |  | (*272*) |
| *Phalacrocorax atriceps* | imperial shag | 110 | 0.0 | 37 | 0.0 | -43.08 | -64.50 | - |  | (*273*) |
| *Phalacrocorax carbo* | great cormorant | 124 | 10.5 | 30 | 30.0 | 51.73 | 18.63 | - | Sibship analyses | (*274*) |
| *Phalaropus lobatus* | red-necked phalarope | 226 | 1.8 | 63 | 6.3 | 66.55 | -163.61 | - |  | (*275*) |
| *Philesturnus carunculatus* | Saddleback | 202 | 0.0 | 39 | 0.0 | -46.90 | 168.10 | 8 |  | (*267*) |
| *Phoebastria irrorata* | waved albatross | 16 | 25.0 | 16 | 25.0 | -1.38 | -89.62 | - |  | (*276*) |
| *Phoebastria irrorata* | waved albatross | 154 | 16.9 | 154 | 16.9 | -1.38 | -89.62 | - |  | (*277*) |
| *Phoenicurus ochruros* | black redstart | 222 | 28.8 | 53 | 30.2 | 47.97 | 11.23 | 3,9 |  | (*278*) |
| *Phoenicurus phoenicurus* | common redstart | 253 | 2.0 | 38 | 10.5 | 62.47 | 11.80 | 1,3,8,9 |  | (*279*) |
| *Phylidonyris pyrrhopterus* | crescent honeyeater | 19 | 57.9 | - | - | -39.03 | 146.33 | 1,3,8,7 |  | (*181*) |
| *Phylloscopus fuscatus* | dusky warbler | 195 | 45.1 | 46 | 58.7 | 59.85 | 154.02 | 8,3,6,11,12 |  | (*280*) |
| *Phylloscopus sibilatrix* | wood warbler | 6 | 16.7 | 3 | 33.3 | 47.42 | 7.55 | 1,3 | Control dataset | (*281*) |
| *Phylloscopus sibilatrix* | wood warbler | 56 | 0.0 | 13 | 0.0 | - | - | 1,3 |  | (*282*) |
| *Phylloscopus trochilus* | willow warbler | 109 | 33.0 | 20 | 50.0 | 61.42 | 8.87 | 1,3,6,10 |  | (*283*) |
| *Phylloscopus trochilus* | willow warbler | 68 | 27.9 | 12 | 58.3 | 56.92 | 18.12 | 1,3,6,10 |  | (*284*) |
| *Phylloscopus trochilus* | willow warbler | 200 | 23.5 | 34 | 47.1 | 56.28 | -2.70 | 1,3,6,10 |  | (*285*) |
| *Phylloscopus trochilus* | willow warbler | 120 | 0.0 | 19 | 0.0 | 59.32 | 18.00 | 1,3,6,10 |  | (*282*) |
| *Picoides tridactylus* | Eurasian three-toed woodpecker | 80 | 2.5 | 26 | 7.7 | 61.18 | 25.10 | - | Some quasi-parasitism | (*286*) |
| *Picoides tridactylus* | Eurasian three-toed woodpecker | 55 | 7.3 | 26 | 15.4 | 47.53 | 12.93 | - |  | (*287*) |
| *Pipilo maculatus* | spotted towhee | 575 | 26.3 | 228 | 44.3 | 45.50 | -122.68 | 6,7,8 |  | (*288*) |
| *Piranga olivacea* | scarlet tanager | 54 | 16.7 | 17 | 29.4 | 41.77 | -79.93 | 1,8,9 |  | (*289*) |
| *Platalea ajaja* | roseate spoonbill | 74 | 2.7 | 37 | 5.4 | -32.48─  1.93 | -50.40─  -56.97 | - | Sibship analyses, sampled over large geographic range | (*29*) |
| *Plectrophenax nivalis* | snow bunting | 380 | 10.8 | 91 | 20.9 | 78.22 | 15.63 | 14,16 |  | (*290*) |
| *Pluvialis dominica* | American golden plover | 131 | 7.6 | 37 | 16.2 | 71.29 | -156.80 | - |  | (*291*) |
| *Poephila acuticauda* | long-tailed finch | 391 | 12.8 | 101 | 25.7 | -15.55 | 128.00 | 2 |  | (*292*) |
| *Porphyrio hochstetteri* | takahe | 27 | 0.0 | 9 | 0.0 | -36.4̶─  -41.08 | 173.88─  174.9 | - | Sampled over large geographic range | (*293*) |
| *Progne subis* | purple martin | 1235 | 22.1 | 297 | 46.1 | 42.13 | -80.30 | 1,3,8 |  | (*294*) |
| *Progne subis* | purple martin | 138 | 18.8 | - | - | 39.08 | -76.57 | 1,3,8 |  | (*295*) |
| *Promerops cafer* | Cape sugarbird | 185 | 64.9 | 104 | 70.2 | -34.00 | 18.97 | 6,9 |  | (*296*) |
| *Prosthemadera novaeseelandiae* | tui | 163 | 55.2 | 57 | 71.9 | -36.37 | 174.83 | 6,8,7,9 |  | (*297*) |
| *Puffinus tenuirostris* | short-tailed shearwater | 83 | 10.8 | 83 | 10.8 | -43.10 | 147.42 | - |  | (*298*) |
| *Pygoscelis adeliae* | Adélie penguin | 22 | 9.1 | 18 | 11.1 | -74.35 | 165.13 | - |  | (*299*) |
| *Pygoscelis antarcticus* | chinstrap penguin | 76 | 0.0 | 38 | 0.0 | -63.00 | -60.67 | - |  | (*300*) |
| *Quelea quelea* | red-billed quelea | 56 | 21.4 | 37 | 32.4 | -21.08 | 31.92 | 4 | Difficult to extract sample sizes | (*301*) |
| *Ramphocelus costaricensis* | Cherrie’s tanager | 55 | 49.1 | 31 | 54.8 | 8.70 | -83.20 | 6,8,4,9 |  | (*302*) |
| *Remiz coronatus* | white-crowned penduline tit | 29 | 0.0 | 5 | 0.0 | 42.42 | 70.48 | 1,3,9 |  | (*303*) |
| *Rhipidura fuliginosa* | grey fantail | 49 | 55.1 | 25 | 64.0 | -35.45 | 149.28 | 1,3,8,13 |  | (*304*) |
| *Riparia riparia* | sand martin | 167 | 13.8 | 45 | 35.6 | 56.20 | -3.98 | 3,9,4 |  | (*305*) |
| *Riparia riparia* | sand martin | 168 | 20.2 | 41 | 36.6 | 48.30 | 21.08 | 3,9,4 |  | (*306*) |
| *Rissa tridactyla* | black-legged kittiwake | 119 | 0.0 | 86 | 0.0 | 48.08 | -4.58 | - |  | (*307*) |
| *Sayornis phoebe* | Eastern phoebe | 769 | 5.1 | 174 | 9.2 | 38.90 | -86.90 | 1,8,3 |  | (*308*) |
| *Sayornis phoebe* | Eastern phoebe | 76 | 11.8 | 20 | 20.0 | 44.57 | -76.32 | 1,8,3 |  | (*309*) |
| *Serinus canaria* | canary | 45 | 0.0 | 15 | 0.0 | 32.58 | -16.47 | 1,8,9 |  | (*310*) |
| *Serinus serinus* | serin | 139 | 9.4 | 47 | 14.9 | 37.87 | -3.92 | 1,9 |  | (*311*) |
| *Serinus serinus* | serin | 61 | 0.0 | 21 | 0.0 | 40.17 | -8.55 | 1,9 |  | (*312*) |
| *Setophaga ruticilla* | American redstart | 81 | 44.4 | 28 | 64.3 | 41.77 | -79.93 | 1,6,8 |  | (*313*) |
| *Setophaga ruticilla* | American redstart | 108 | 39.8 | 32 | 59.4 | 45.07 | -67.03 | 1,6,8 |  | (*314*) |
| *Setophaga ruticilla* | American redstart | 239 | 23.4 | 75 | 42.7 | 44.57 | -76.32 | 1,6,8 |  | (*315*) |
| *Sialia currucoides* | mountain bluebird | 465 | 36.3 | 92 | 71.7 | 44.63 | -107.00 | 3,4,2,1 |  | (*316*) |
| *Sialia currucoides* | mountain bluebird | 900 | 32.0 | 177 | 65.0 | 51.00 | -122.00 | 3,4,2,1 | Control dataset | (*317*) |
| *Sialia sialis* | Eastern bluebird | 83 | 8.4 | 21 | 23.8 | 44.57 | -76.32 | 1,2,3,9 |  | (*318*) |
| *Sitta europaea* | European nuthatch | 188 | 9.6 | 32 | 37.5 | 48.53 | 9.05 | 1,8 |  | (*319*) |
| *Spheniscus humboldti* | Humboldt penguin | 49 | 0.0 | 21 | 0.0 | -15.37 | -75.20 | - |  | (*320*) |
| *Spiza americana* | dickcissel | 218 | 38.5 | 92 | 52.2 | 39.08 | -96.58 | 4 |  | (*321*) |
| *Spizella pusilla* | field sparrow | 308 | 9.7 | 80 | 40.0 | 40.20 | -87.72 | 1,6,9 |  | (*322*) |
| *Steganopus tricolor* | Wilson’s phalarope | 43 | 0.0 | 15 | 0.0 | 51.45 | -105.17 | - |  | (*323*) |
| *Sterna hirundo* | common tern | 102 | 1.0 | 34 | 2.9 | 53.52 | 8.08 | - |  | (*324*) |
| *Sterna hirundo* | common tern | 29 | 0.0 | 10 | 0.0 | 45.35 | 12.27 | - |  | (*325*) |
| *Strix aluco* | tawny owl | 137 | 0.7 | 37 | 2.7 | 46.00 | 6.00 | - |  | (*326*) |
| *Sturnus unicolor* | spotless starling | 334 | 15.9 | 96 | 41.7 | 40.50 | -4.00 | 1,3,9 |  | (*327*) |
| *Sturnus unicolor* | spotless starling | 202 | 15.3 | 20 | 65.0 | 40.50 | -4.00 | 1,3,9 | Control dataset | (*328*) |
| *Sturnus vulgaris* | common starling | 196 | 16.8 | 48 | 43.8 | 46.52 | 6.57 | 1,3,9 |  | (*329*) |
| *Sturnus vulgaris* | common starling | 62 | 9.7 | 14 | 28.6 | 51.22 | 4.67 | 1,3,9 |  | (*330*) |
| *Sturnus vulgaris* | common starling | 157 | 15.9 | 37 | 40.5 | 55.72 | 13.45 | 1,3,9 |  | (*331*) |
| *Sula dactylatra* | masked booby | 6 | 0.0 | 6 | 0.0 | -18.17 | -39.33 | - |  | (*332*) |
| *Sula granti* | Nazca booby | 32 | 0.0 | 21 | 0.0 | -1.33 | -89.67 | - |  | (*333*) |
| *Sula nebouxii* | blue-footed booby | 799 | 6.9 | 453 | 10.6 | 21.87 | -105.90 | - |  | (*334*) |
| *Sula sula* | red-footed booby | 14 | 0.0 | 14 | 0.0 | -1.33 | -89.67 | - |  | (*335*) |
| *Tachycineta albilinea* | mangrove swallow | 97 | 15.5 | 31 | 25.8 | 9.17 | -79.85 | 1,3 |  | (*336*) |
| *Tachycineta bicolor* | tree swallow | 63 | 33.3 | 11 | 63.6 | 44.57 | -76.32 | 3,12 | Control dataset | (*337*) |
| *Tachycineta bicolor* | tree swallow | 111 | 68.5 | 25 | 84.0 | 44.70 | -76.20 | 3,12 | Data from natural nest holes | (*338*) |
| *Tachycineta bicolor* | tree swallow | 529 | 51.4 | 106 | 74.5 | 44.57 | -76.32 | 3,12 |  | (*339*) |
| *Tachycineta bicolor* | tree swallow | 67 | 55.2 | 13 | 84.6 | 44.58 | -66.77 | 3,12 |  | (*339*) |
| *Tachycineta bicolor* | tree swallow | 365 | 47.9 | 67 | 82.1 | 44.57 | -76.32 | 3,12 |  | (*340*) |
| *Tachycineta bicolor* | tree swallow | - | - | 23 | 69.6 | 43.38 | -88.02 | 3,12 |  | (*341*) |
| *Tachycineta bicolor* | tree swallow | 3438 | 49.0 | 587 | 84.0 | 45.00 | -74.00 | 3,12 |  | (*342*) |
| *Tachycineta bicolor* | tree swallow | 216 | 35.2 | 40 | 85.0 | 53.00 | -123.00 | 3,12 |  | (*343*) |
| *Tachycineta bicolor* | tree swallow | 502 | 47.2 | 99 | 82.8 | 44.57 | -76.32 | 3,12 |  | (*344*) |
| *Tachycineta bicolor* | tree swallow | - | - | 248 | 83.9 | 45.00 | -74.00 | 3,12 |  | (*345*) |
| *Tachycineta bicolor* | tree swallow | 281 | 48.8 | 54 | 88.9 | 43.38 | -88.02 | 3,12 |  | (*346*) |
| *Tachycineta bicolor* | tree swallow | 439 | 38.5 | - | - | 43.38 | -88.02 | 3,12 |  | (*347*) |
| *Tachycineta leucorrhoa* | white rumped swallow | 342 | 56.4 | 78 | 76.9 | -35.57 | -58.02 | 3,9,12 |  | (*348*) |
| *Tachycineta meyeni* | Chilean swallow | 161 | 6.8 | 52 | 13.5 | -54.73 | -68.20 | 3,9,12 |  | (*349*) |
| *Taeniopygia guttata* | zebra finch | 82 | 2.4 | 25 | 8.0 | -36.15 | 145.43 | 3,4 |  | (*350*) |
| *Taeniopygia guttata* | zebra finch | 316 | 1.6 | 80 | 5.0 | -31.08 | 142.70 | 3,4 |  | (*351*) |
| *Thalassarche cauta* | shy albatross | 29 | 6.9 | 29 | 6.9 | -40.38 | 144.65 | - |  | (*352*) |
| *Thalassarche chrysostoma* | grey-headed albatross | 83 | 7.2 | 90 | 6.7 | -54.00 | -38.00 | - |  | (*86*) |
| *Thalassarche melanophrys* | black-browed albatross | 87 | 5.7 | 90 | 5.6 | -54.00 | -38.00 | - |  | (*86*) |
| *Thalassoica antarctica* | Antarctic petrel | 41 | 7.3 | 41 | 7.3 | -71.88 | 5.17 | - |  | (*353*) |
| *Thamnophilus atrinucha* | black-crowned antshrike | 89 | 3.4 | 50 | 4.0 | 9.22 | -79.65 | 8 |  | (*354*) |
| *Thryothorus ludovicianus* | Carolina wren | 84 | 0.0 | 23 | 0.0 | 34.82 | -87.63 | 1,8,9 |  | (*355*) |
| *Thryothorus pleurostictus* | banded wren | 156 | 4.5 | 50 | 10.0 | 10.67 | -85.50 | 6,8 |  | (*356*) |
| *Thryothorus rufalbus* | rufous-and-white wren | 158 | 1.9 | 51 | 5.9 | 10.67 | -85.50 | 8 |  | (*357*) |
| *Tockus monteiri* | Monteiro’s hornbill | 135 | 0.0 | 38 | 0.0 | -22.53 | 16.97 | - |  | (*358*) |
| *Troglodytes aedon* | house wren | 1772 | 16.4 | 361 | 35.2 | 40.67 | -88.88 | 1,3,8,9 |  | (*359*) |
| *Troglodytes aedon* | house wren | 54 | 1.9 | 9 | 11.1 | 49.38 | -88.02 | 1,3,8,9 | Control dataset | (*360*) |
| *Troglodytes aedon* | house wren | 857 | 13.5 | 181 | 37.6 | 42.52 | -76.47 | 1,3,8,9 |  | (*361*) |
| *Troglodytes aedon* | house wren | - | - | 46 | 45.7 | 44.68 | -106.98 | 1,3,8,9 | Control dataset | (*362*) |
| *Troglodytes aedon* | house wren | 316 | 12.7 | 79 | 63.3 | 44.67 | -105.93 | 1,3,8,9 |  | (*363*) |
| *Troglodytes aedon* | house wren | 377 | 24.9 | 82 | 53.7 | 42.52 | -76.47 | 1,3,8,9 |  | (*364*) |
| *Troglodytes aedon* | house wren | 166 | 15.7 | 40 | 32.5 | -36.43 | -56.94 | 1,3,8,9 |  | (*364*) |
| *Troglodytes aedon* | house wren | 584 | 10.1 | 103 | 28.2 | 43.38 | -88.02 | 1,3,8,9 |  | (*365*) |
| *Troglodytes aedon* | house wren | 790 | 8.4 | 146 | 26.7 | 40.67 | -88.88 | 1,3,8,9 |  | (*366*) |
| *Troglodytes troglodytes* | Eurasian wren | 153 | 16.3 | 29 | 37.9 | 53.13 | 6.58 | 1,3,8,9 |  | (*367*) |
| *Turdus albicollis* | white-necked thrush | 22 | 18.2 | 11 | 36.4 | -24.07 | -47.97 | 1,3,6,8,9 |  | (*368*) |
| *Turdus grayi* | clay-colored robin | 37 | 37.8 | 19 | 52.6 | 9.12 | -79.70 | 1,3,8,9 |  | (*369*) |
| *Turdus migratorius* | American robin | 187 | 48.1 | 64 | 71.9 | 40.03 | -88.17 | 1,3,8,9 |  | (*370*) |
| *Tyrannus forficatus* | scissor-tailed flycatchers | 168 | 48.8 | 44 | 65.9 | 34.77 | -98.48 | 1,3,2,4,15 | Text reports 42 nests, but table reports 44 | (*371*) |
| *Tyrannus tyrannus* | Eastern kingbird | 264 | 47.0 | 89 | 60.7 | 43.00 | -119.00 | 1,3,8,9,15 |  | (*372*) |
| *Tyrannus tyrannus* | Eastern kingbird | 64 | 42.2 | 20 | 60.0 | 43.30 | -74.88 | 1,3,8,9,15 |  | (*373*) |
| *Tyto alba* | barn owl | 455 | 1.3 | 95 | 2.1 | 46.82 | 6.95 | - |  | (*374*) |
| *Upupa epops* | Eurasian hoopoe | 254 | 3.9 | 41 | 17.1 | 46.33 | 7.67 | - |  | (*375*) |
| *Upupa epops* | Eurasian hoopoe | 126 | 7.1 | 36 | 13.9 | 37.00 | -3.00 | - |  | (*376*) |
| *Uria aalge* | common murres | 77 | 7.8 | 77 | 7.8 | 51.75 | -5.28 | - |  | (*377*) |
| *Uria lomvia* | thick-billed murres | 27 | 7.4 | 27 | 7.4 | 62.00 | -83.00 | - |  | (*378*) |
| *Vermivora chrysoptera* | golden-winged warbler | 62 | 38.7 | 17 | 76.5 | 44.57 | -76.32 | 1,6 |  | (*379*) |
| *Vireo griseus* | white-eyed vireo | 102 | 2.0 | 36 | 5.6 | 31.40 | -97.80 | 6,7 |  | (*380*) |
| *Vireo olivaceus* | red-eyed vireo | 19 | 57.9 | 7 | 57.1 | 41.00 | -79.00 | 8 |  | (*381*) |
| *Vireo solitarius* | blue-headed vireo | 37 | 2.7 | 16 | 6.2 | 41.00 | -79.00 | 8 |  | (*381*) |
| *Volatinia jacarina* | blue-black grassquits | 20 | 50.0 | 11 | 63.6 | -15.95 | -47.93 | 4,6 |  | (*382*) |
| *Volatinia jacarina* | blue-black grassquits | 208 | 21.2 | 95 | 30.5 | -15.93 | -47.93 | 4,6 |  | (*383*) |
| *Wilsonia citrina* | hooded warbler | 356 | 26.7 | 119 | 35.3 | 41.00 | -79.00 | 1,7,8 |  | (*384*) |
| *Zonotrichia albicollis* | white-throated sparrow | 1183 | 15.4 | 379 | 26.6 | 44.15 | -74.78 | 8,9 |  | (*385*) |
| *Zonotrichia albicollis* | white-throated sparrow | 89 | 18.0 | 32 | 31.2 | 44.15 | -74.78 | 8,9 | Data for two morphs combined | (*386*) |
| *Zonotrichia capensis* | rufous-collared sparrow | 24 | 41.7 | 11 | 63.6 | -0.35 | -78.15 | 6,3,9 |  | (*387*) |
| *Zonotrichia capensis* | rufous-collared sparrow | 23 | 52.2 | 10 | 60.0 | -0.12 | -77.92 | 6,3,9 |  | (*387*) |
| *Zonotrichia leucophrys* | white-crowned sparrow | 68 | 47.1 | 28 | 64.3 | 48.02 | -122.05 | 1,6,9 |  | (*42*, *388*) |
| *Zonotrichia leucophrys* | white-crowned sparrow | 342 | 41.2 | 96 | 44.8 | 38.57 | -119.03 | 1,6,9 |  | (*389*) |
| *Zonotrichia leucophrys* | white-crowned sparrow | 189 | 29.1 | 62 | 38.7 | 43.16 | -124.41 | 1,6,9 |  | (*390*) |
| *Zosterops lateralis* | capricorn silvereye | 122 | 0.0 | 53 | 0.0 | -23.43 | 151.90 | 1,3,6,8,9 |  | (*391*) |

References

1. J. del Hoyo, A. Elliott, J. Sargatal, *Handbook of the birds of the world.* (Lynx Edit., Barcelona, 1992).

2. D. J. Green, E. A. Krebs, A. Cockburn, Mate choice in the brown thornbill ( Acanthiza pusilla ): are settlement decisions, divorce and extrapair mating complementary strategies? *Behav. Ecol. Sociobiol.* **55**, 278–285 (2004).

3. R. N. Rosenfield, S. A. Sonsthagen, W. E. Stout, S. L. Talbot, High frequency of extra-pair paternity in an urban population of Cooper’s Hawks: Extra-Pair Paternity in Cooper’s Hawks. *J. Field Ornithol.* **86**, 144–152 (2015).

4. B. Leisler, Variation in extra-pair paternity in the polygynous Great Reed Warbler (Acrocephalus arundinaceus). *J. Für Ornithol.* **141**, 77 (2000).

5. D. Hasselquist, S. Bensch, T. von Schantz, Correlation between male song repertoire, extra-pair paternity and offspring survival in the great reed warbler. *Nature*. **381**, 229–232 (1996).

6. B. Hansson, D. Hasselquist, S. Bensch, Do female great reed warblers seek extra-pair fertilizations to avoid inbreeding? *Proc. R. Soc. Lond. Ser. B-Biol. Sci.* **271**, S290–S292 (2004).

7. S. Hamao, D. S. Saito, Extrapair fertilization in the black-browed reed warbler (Acrocephalus bistrigiceps): effects on mating status and nesting cycle of cuckolded and cuckolder males. *The Auk*. **122**, 1086 (2005).

8. B. Leisler, M. Wink, Frequencies of multiple paternity in three *Acrocephalus* species (Aves Sylviidae) with different mating systems ( *A. palustris, A. arundinaceus, A. paludicola* ). *Ethol. Ecol. Evol.* **12**, 237–249 (2000).

9. A. Langefors, D. Hasselquist, T. von Schantz, Extra-pair fertilizations in the Sedge Warbler. *J. Avian Biol.* **29**, 134–144 (1998).

10. R. C. Marshall, K. L. Buchanan, C. K. Catchpole, Song and female choice for extrapair copulations in the sedge warbler, Acrocephalus schoenobaenus. *Anim. Behav.* **73**, 629–635 (2007).

11. N. B. Davies, S. H. M. Butchart, T. A. Burke, N. Chaline, I. R. K. Stewart, Reed warblers guard against cuckoos and cuckoldry. *Anim. Behav.* **65**, 285–295 (2003).

12. A. Mee, D. P. Whitfield, D. B. A. Thompson, T. Burke, Extrapair paternity in the common sandpiper, Actitis hypoleucos, revealed by DNA fingerprinting. *Anim. Behav.* **67**, 333–342 (2004).

13. D. Blomqvist, M. Andersson, C. Küpper, I. C. Cuthill, J. Kis, R. B. Lanctot, B. K. Sandercock, T. Székely, J. Wallander, B. Kempenaers, Genetic similarity between mates and extra-pair parentage in three species of shorebirds. *Nature*. **419**, 613–615 (2002).

14. M. E. Koopman, D. B. McDonald, G. D. Hayward, Microsatellite Analysis Reveals Genetic Monogamy Among Female Boreal Owls. *J. Raptor Res.* **41**, 314–318 (2007).

15. I. A. Liu, J. E. Johndrow, J. Abe, S. Lüpold, K. Yasukawa, D. F. Westneat, S. Nowicki, Genetic diversity does not explain variation in extra-pair paternity in multiple populations of a songbird. *J. Evol. Biol.* **28**, 1156–1169 (2015).

16. D. F. Westneat, Polygyny and extrapair fertilizations in eastern red-winged blackbirds (Agelaius phoeniceus). *Behav. Ecol.* **4**, 49–60 (1993).

17. P. J. Weatherhead, P. T. Boag, Pair and extra-pair mating success relative to male quality in red-winged blackbirds. *Behav. Ecol. Sociobiol.* **37**, 81–91 (1995).

18. E. M. Gray, Female control of offspring paternity in a western population of red-winged blackbirds (Agelaius phoeniceus). *Behav. Ecol. Sociobiol.* **38**, 267–278 (1996).

19. I. A. Liu, Conservation genetics and genetic mating system of the yellow-shouldered blackbird (Agelaius xanthomus), an endangered island endemic. *Conserv. Genet.* **16**, 1041–1053 (2015).

20. J. M. C. Hutchinson, S. C. Griffith, Extra-pair paternity in the Skylark Alauda arvensis: Extra-pair paternity in the Skylark. *Ibis*. **150**, 90–97 (2007).

21. J. T. Lifjeld, Ann M. A. Harding, F. Mehlum, T. Øigarden, No Evidence of Extra-Pair Paternity in the Little Auk Alle alle. *J. Avian Biol.* **36**, 484–487 (2005).

22. K. Wojczulanis-Jakubas, D. Jakubas, T. Øigarden, J. T. Lifjeld, Extrapair copulations are frequent but unsuccessful in a highly colonial seabird, the little auk, Alle alle. *Anim. Behav.* **77**, 433–438 (2009).

23. C. E. Hill, W. Post, Extra-pair paternity in Seaside Sparrows. *J. Field Ornithol.* **76**, 119–126 (2005).

24. J. Kreisinger, P. Munclinger, V. Javůrková, T. Albrecht, Analysis of extra-pair paternity and conspecific brood parasitism in mallards Anas platyrhynchos using non-invasive techniques. *J. Avian Biol.* **41**, 551–557 (2010).

25. J. L. Peters, G. L. Brewer, L. M. Bowe, Extrapair Paternity and Breeding Synchrony in Gadwalls (Anas strepera) in North Dakota. *The Auk*. **120**, 883–888 (2003).

26. H. U. Reyer, K. Bollmann, A. R. Schlapfer, A. Schymainda, G. Klecack, Ecological determinants of extrapair fertilizations and egg dumping in Alpine water pipits (Anthus spinoletta). *Behav. Ecol.* **8**, 534–543 (1997).

27. Thaís L. F. Martins, J. K. Blakey, J. Wright, Low Incidence of Extra-Pair Paternity in the Colonially Nesting Common Swift Apus apus. *J. Avian Biol.* **33**, 441–446 (2002).

28. J. A. Rudnick, T. E. Katzner, E. A. Bragin, O. E. Rhodes, J. A. Dewoody, Using naturally shed feathers for individual identification, genetic parentage analyses, and population monitoring in an endangered Eastern imperial eagle (Aquila heliaca) population from Kazakhstan. *Mol. Ecol.* **14**, 2959–2967 (2005).

29. C. I. Miño, M. A. Russello, P. F. Mussi Gonçalves, S. N. Del Lama, Reconstructing genetic mating systems in the absence of parental information in colonially breeding waterbirds. *BMC Evol. Biol.* **11** (2011), doi:10.1186/1471-2148-11-196.

30. J. S. Marks, J. L. Dickinson, J. Haydock, Genetic Monogamy in Long-Eared Owls. *The Condor*. **101**, 854–859 (1999).

31. S. Rodriguez-Martínez, M. Carrete, S. Roques, N. Rebolo-Ifrán, J. L. Tella, High Urban Breeding Densities Do Not Disrupt Genetic Monogamy in a Bird Species. *PLoS ONE*. **9**, e91314 (2014).

32. W. Müller, J. T. Epplen, T. Lubjuhn, Genetic paternity analyses in Little Owls (Athene noctua): does the high rate of paternal care select against extra-pair young? *J. Ornithol.* **142**, 195–203 (2001).

33. E. V. Pravosudova, P. G. Parker, A. S. Gaunt, Genetic Evidence for Extrapair Paternity in the Tufted Titmouse. *Wilson Bull.* **114**, 279–281 (2002).

34. A. E. Casey, B. K. Sandercock, S. M. Wisely, Genetic Parentage and Local Population Structure in the Socially Monogamous Upland Sandpiper. *The Condor*. **113**, 119–128 (2011).

35. P. R. Lemons, T. C. Marshall, S. E. McCloskey, S. A. Sethi, J. A. Schmutz, J. S. Sedinger, A likelihood-based approach for assessment of extra-pair paternity and conspecific brood parasitism in natural populations. *Mol. Ecol. Resour.* **15**, 107–116 (2015).

36. J. A. Moore, A. M. Kamarainen, K. T. Scribner, C. Mykut, H. H. Prince, The effects of anthropogenic alteration of nesting habitat on rates of extra-pair fertilization and intraspecific brood parasitism in Canada Geese Branta canadensis: Alternative reproductive strategies in Canada Geese. *Ibis*. **154**, 354–362 (2012).

37. S. Choudhury, C. S. Jones, J. M. Black, J. Prop, Adoption of Young and Intraspecific Nest Parasitism in Barnacle Geese. *The Condor*. **95**, 860–868 (1993).

38. K. Larsson, H. Tegelström, P. Forslund, Intraspecific nest parasitism and adoption of young in the barnaele goose: effects on survival and reproductive performance. *Anim. Behav.* **50**, 1349–1360 (1995).

39. L. G. Woolaver, R. K. Nichols, E. S. Morton, B. J. M. Stutchbury, Social and genetic mating system of Ridgway’s hawk (Buteo ridgwayi), an endemic raptor on Hispaniola. *J. Trop. Ecol.* **29**, 531–540 (2013).

40. C. W. Briggs, M. W. Collopy, Extra-pair paternity in Swainson’s Hawks: Swainson’s Hawk Extra-Pair Paternity. *J. Field Ornithol.* **83**, 41–46 (2012).

41. A. S. Chaine, B. E. Lyon, Adaptive Plasticity in Female Mate Choice Dampens Sexual Selection on Male Ornaments in the Lark Bunting. *Science*. **319**, 459–462 (2008).

42. F. Bonier, C. Eikenaar, P. R. Martin, I. T. Moore, Extrapair Paternity Rates Vary with Latitude and Elevation in Emberizid Sparrows. *Am. Nat.* **183**, 54–61 (2014).

43. J. Reneerkens, P. van Veelen, M. van der Velde, P. Luttikhuizen, T. Piersma, Within-population variation in mating system and parental care patterns in the Sanderling ( *Calidris alba* ) in northeast Greenland. *The Auk*. **131**, 235–247 (2014).

44. E. P. Pierce, J. T. Lifjeld, High Paternity without Paternity-Assurance Behavior in the Purple Sandpiper, a Species with High Paternal Investment. *The Auk*. **115**, 602–612 (1998).

45. C. A. Davis, J. P. Orange, R. A. Van Den Bussche, R. D. Elmore, S. D. Fuhlendorf, J. M. Carroll, E. P. Tanner, D. M. Leslie, Extrapair paternity and nest parasitism in two sympatric quail. *The Auk*. **134**, 811–820 (2017).

46. J. Bried, M.-P. Dubois, P. Jarne, P. Jouventin, R. S. Santos, Does competition for nests affect genetic monogamy in Cory’s shearwater Calonectris diomedea? *J. Avian Biol.* **41**, 407–418 (2010).

47. C. Rabouam, V. Bretagnolle, Y. Bigot, G. Periquet, Genetic Relationships of Cory’s Shearwater: Parentage, Mating Assortment, and Geographic Differentiation Revealed by DNA Fingerprinting. *The Auk*. **117**, 651–662 (2000).

48. I. Swatschek, D. Ristow, M. Wink, Mate fidelity and parentage in Cory’s shearwater Calonectris diomedea - field studies and DNA fingerprinting. *Mol. Ecol.* **3**, 259–262 (1994).

49. G. Ritchison, P. H. Klatt, D. F. Westneat, Mate guarding and extra-pair paternity in northern cardinals. *Condor*. **96**, 1055–1063 (1994).

50. J. Bonlokke-Pedersen, J. Drachmann, J. Frydenberg, J. J. Boomsma, Rare extra-pair fertilizations in the semi-colonially breeding linnet Carduelis cannabina. *J. Avian Biol.* **33**, 203–206 (2002).

51. G. J. Gissing, T. J. Crease, A. L. A. Middleton, Extrapair paternity associated with renesting in the American Goldfinch. *Auk*. **115**, 230–234 (1998).

52. J. C. Winternitz, M. Promerova, R. Polakova, M. Vinker, J. Schnitzer, P. Munclinger, W. Babik, J. Radwan, J. Bryja, T. Albrecht, Effects of heterozygosity and MHC diversity on patterns of extra-pair paternity in the socially monogamous scarlet rosefinch. *Behav. Ecol. Sociobiol.* **69**, 459–469 (2015).

53. E. R. Lindstedt, K. P. Oh, A. V. Badyaev, Ecological, social, and genetic contingency of extrapair behavior in a socially monogamous bird. *J. Avian Biol.* **38**, 214–223 (2007).

54. G. E. Hill, R. Montgomerie, C. Roeder, P. Boag, Sexual selection and cuckoldry in a monogamous songbird - implications for sexual selection theory. *Behav. Ecol. Sociobiol.* **35**, 193–199 (1994).

55. C. Millar, Patterns of reproductive success determined by DNA fingerprinting in a communally breeding oceanic bird. *Biol. J. Linn. Soc.* **52**, 31–48 (1994).

56. C. D. Millar, D. M. Lambert, E. C. Young, Minisatellite DNA Detects Sex, Parentage, and Adoption in the South Polar Skua. *J. Hered.* **88**, 235–238 (1997).

57. G. Maurer, M. C. Double, O. Milenkaya, M. Süsser, R. D. Magrath, Breaking the rules: sex roles and genetic mating system of the pheasant coucal. *Oecologia*. **167**, 413–425 (2011).

58. T. Anker-Nilssen, O. Kleven, T. Aarvak, J. T. Lifjeld, Low or no occurrence of extra-pair paternity in the Black Guillemot Cepphus grylle. *J. Ornithol.* **151**, 247–250 (2010).

59. R. C. Fleischer, C. L. Tarr, E. S. Morton, A. Sangmeister, K. C. Derrickson, Mating System of the Dusky Antbird, a Tropical Passerine, as Assessed by DNA Fingerprinting. *The Condor*. **99**, 512–514 (1997).

60. K. H. Maher, L. J. Eberhart-Phillips, A. Kosztolányi, N. dos Remedios, M. C. Carmona-Isunza, M. Cruz-López, S. Zefania, J. J. H. St Clair, M. Alrashidi, M. A. Weston, M. A. Serrano-Meneses, O. Krüger, J. I. Hoffman, T. Székely, T. Burke, C. Küpper, High fidelity: extra-pair fertilisations in eight *Charadrius* plover species are not associated with parental relatedness or social mating system. *J. Avian Biol.* **48**, 910–920 (2017).

61. J. Wallander, D. Blomqvist, J. T. Lifjeld, Genetic and Social Monogamy - Does It Occur Without Mate Guarding in the Ringed Plover? *Ethology*. **107**, 561–572 (2001).

62. Y. Zharikov, E. Nol, Copulation Behavior, Mate Guarding, and Paternity in the Semipalmated Plover. *The Condor*. **102**, 231–235 (2000).

63. P. O. Dunn, A. D. Afton, M. L. Gloutney, R. T. Alisaukas, Forced copulation results in few extrapair fertilizations in Ross’s and lesser snow geese. *Anim. Behav.* **57**, 1071–1081 (1999).

64. P. Minias, A. Minias, J. Dziadek, Occurrence of extra-pair paternity and intraspecific brood parasitism in the Whiskered Tern *Chlidonias hybrida*. *Bird Study*. **61**, 130–134 (2014).

65. D. A. Shealer, S. Devbhandari, M. G. Garcia-Mendoza, Evidence for Genetic Monogamy But Low Mate Retention in the North American Black Tern (*Chlidonias niger surinamensis*). *Waterbirds*. **37**, 129–135 (2014).

66. S. F. Turjeman, A. Centeno-Cuadros, U. Eggers, S. Rotics, J. Blas, W. Fiedler, M. Kaatz, F. Jeltsch, M. Wikelski, R. Nathan, Extra-pair paternity in the socially monogamous white stork (Ciconia ciconia) is fairly common and independent of local density. *Sci. Rep.* **6**, 27976 (2016).

67. T. Øigarden, T. Borge, J. T. Lifjeld, Extrapair paternity and genetic diversity: the white-throated dipper Cinclus cinclus. *J. Avian Biol.* **41**, 248–257 (2010).

68. R. Rutkowski, D. Krupiński, I. Kitowski, D. Popović, A. Gryczyńska, M. Molak, B. Dulisz, K. Poprach, S. Müller, R. Müller, K.-D. Gierach, Genetic structure and diversity of breeding Montagu’s harrier (Circus pygargus) in Europe. *Eur. J. Wildl. Res.* **61**, 691–701 (2015).

69. K. L. Wiebe, B. Kempenaers, The social and genetic mating system in flickers linked to partially reversed sex roles. *Behav. Ecol.* **20**, 453–458 (2009).

70. A. Sánchez-Tójar, D. Parejo, J. G. Martínez, J. Rodríguezruiz, J. M. Avilés, Parentage Analyses Reveal Hidden Breeding Strategies of European Rollers *Coracias garrulus*. *Acta Ornithol.* **50**, 252–258 (2015).

71. M. D. Decker, et al., Monogamy in Black Vultures: genetic evidence from DNA fingerprinting. *Behav. Ecol.* **4**, 29–35 (1993).

72. D. Liebers, H.-U. Peter, Intraspecific interactions in Jackdaws Corvus monedula: a field study combined with parentage analysis. *Ardea*. **86**, 221–235 (1999).

73. I. G. Henderson, P. J. B. Hart, T. Burke, Strict monogamy in a semi-colonial passerine: the Jackdaw Corvus monedula. *J. Avian Biol.* **31**, 177–182 (2000).

74. K. R. Overeem, P. O. Gabriel, J. A. Zirpoli, J. M. Black, Steller Sex: Infidelity and Sexual Selection in a Social Corvid (Cyanocitta stelleri). *PLoS ONE*. **9**, e105257 (2014).

75. J. F. Masello, A. Sramkova, P. Quillfeldt, J. T. Epplen, T. Lubjuhn, Genetic monogamy in burrowing parrots Cyanoliseus patagonus? *J. Avian Biol.* **33**, 99–103 (2002).

76. K. Kraaijeveld, P. J. Carew, T. Billing, G. J. Adcock, R. A. Mulder, Extra-pair paternity does not result in differential sexual selection in the mutually ornamented black swan (Cygnus atratus). *Mol. Ecol.* **13**, 1625–1633 (2004).

77. H. T. Riley, D. M. Bryant, R. E. Carter, D. T. Parkin, Extra-pair fertilizations and paternity defense in house martins, Delichon urbica. *Anim. Behav.* **49**, 495–509 (1995).

78. L. A. Whittingham, J. T. Lifjeld, High paternal investment in unrelated young: extra-pair paternity and male parental care in house martins. *Behav. Ecol. Sociobiol.* **37**, 103–108 (1995).

79. Klaus G. Michalek, H. Winkler, Parental Care and Parentage in Monogamous Great Spotted Woodpeckers (Picoides major) and Middle Spotted Woodpeckers (Picoides medius). *Behaviour*. **138**, 1259–1285 (2001).

80. M. S. Webster, H. C. Chuang-Dobbs, R. T. Holmes, Microsatellite identification of extrapair sires in a socially monogamous warbler. *Behav. Ecol.* **12**, 439–446 (2001).

81. H. C. Chuang, M. S. Webster, R. T. Holmes, Extrapair paternity and local synchrony in the Black-throated Blue Warbler. *Auk*. **116**, 726–736 (1999).

82. B. E. Byers, H. L. Mays, I. R. K. Stewart, D. F. Westneat, Extrapair paternity increases variability in male reproductive success in the chestnut-slded warbler (Dendroica pensylvanica), a socially monogamous songbird. *Auk*. **121**, 788–795 (2004).

83. A. S. Grunst, M. L. Grunst, Multiple sexual pigments, assortative social pairing, and genetic paternity in the yellow warbler (Setophaga petechia). *Behav. Ecol. Sociobiol.* **68**, 1451–1463 (2014).

84. S. M. Yezerinac, H. L. Gibbs, J. V. Briskie, R. Whittam, R. Montgomerie, Extrapair paternity in a far northern population of yellow warblers Dendroica petechia. *J. Avian Biol.* **30**, 234–237 (1999).

85. S. M. Yezerinac, P. J. Weatherhead, Extra-pair mating, male plumage coloration and sexual selection in yellow warblers (Dendroica petechia). *Proc. R. Soc. Lond. Ser. B-Biol. Sci.* **264**, 527–532 (1997).

86. T. M. Burg, J. P. Croxall, Extrapair Paternities in Black-Browed Thalassarche melanophris, Grey-Headed T. chrysostoma and Wandering Albatrosses Diomedea exulans at South Georgia. *J. Avian Biol.* **37**, 331–338 (2006).

87. M. G. W. Jones, N. M. S. M. Techow, P. G. Ryan, Dalliances and doubtful dads: what determines extra-pair paternity in socially monogamous wandering albatrosses? *Behav. Ecol. Sociobiol.* **66**, 1213–1224 (2012).

88. P. Jouventin, A. Charmantier, M.-P. Dubois, P. Jarne, J. Bried, Extra-pair paternity in the strongly monogamous Wandering Albatross Diomedea exulans has no apparent benefits for females: Extra-pair paternity in Wandering Albatrosses. *Ibis*. **149**, 67–78 (2006).

89. T. B. Ryder, R. C. Fleischer, W. G. Shriver, P. P. Marra, The ecological-evolutionary interplay: density-dependent sexual selection in a migratory songbird: Density-Dependent Sexual Selection in a Migratory Songbird. *Ecol. Evol.* **2**, 976–987 (2012).

90. B. J. M. Stutchbury, E. S. Morton, B. Woolfenden, Comparison of the Mating Systems and Breeding Behavior of a Resident and a Migratory Tropical Flycatcher. *J. Field Ornithol.* **78**, 40–49 (2007).

91. J. Sundberg, A. Dixon, Old, colourful male yellowhammers, Emberiza citrinella, benefit from extra-pair copulations. *Anim. Behav.* **52**, 113–122 (1996).

92. A. Dixon, D. Ross, S. L. C. O’Malley, T. Burke, Paternal investment inversely related to degree of extra-pair paternity in the reed bunting. *Nature*. **371**, 698–700 (1994).

93. K. M. Bouwman, T. Burke, J. Komdeur, How reed buntings benefit from extra-pair mating behaviour: testing hypotheses through patterns of paternity in sequential broods. *Mol. Ecol.* **15**:2589-600 (2006).

94. S. M. Suter, M. Keiser, R. Feignoux, D. R. Meyer, Reed bunting females increase fitness through extra-pair mating with genetically dissimilar males. *Proc. R. Soc. B Biol. Sci.* **274**, 2865–2871 (2007).

95. C. Mayer, G. Pasinelli, New support for an old hypothesis: density affects extra-pair paternity. *Ecol. Evol.* **3**, 694–705 (2013).

96. O. Kleven, J. T. Lifjeld, No evidence for increased offspring heterozygosity from extrapair mating in the reed bunting (Emberiza schoeniclus). *Behav. Ecol.* **16**, 561–565 (2005).

97. S. A. Tarof, L. M. Ratcliffe, M. M. Kasumovic, P. T. Boag, Are least flycatcher (Empidonax minimus) clusters hidden leks? *Behav. Ecol.* **16**, 207–217 (2005).

98. T. Pearson, M. J. Whitfield, T. C. Theimer, P. Keim, Polygyny and extra-pair paternity in a population of southwestern willow flycatchers. *The Condor*. **108**, 571 (2006).

99. S. Hung, S. A. Tarof, B. J. M. Stutchbury, Extra-Pair Mating Tactics and Vocal Behavior of Female Acadian Flycatchers - Tácticas de Apareamiento Extra-Pareja y Comportamiento Vocal de las Hembras de Empidonax virescens. *The Condor*. **111**, 653–661 (2009).

100. M. L. Evans, B. E. Woolfenden, L. Friesen, B. J. M. Stutchbury, Variation in the extra-pair mating systems of Acadian Flycatchers and Wood Thrushes in forest fragments in southern Ontario. *J. Field Ornithol.* **80**, 146–153 (2009).

101. B. E. Woolfenden, B. J. M. Stutchbury, E. S. Morton, Male Acadian flycatchers, Empidonax virescens, obtain extrapair fertilizations with distant females. *Anim. Behav.* **69**, 921–929 (2005).

102. P. E. Bolton, L. A. Rollins, J. Brazill-Boast, K.-W. Kim, T. Burke, S. C. Griffith, The colour of paternity: extra-pair paternity in the wild Gouldian finch does not appear to be driven by genetic incompatibility between morphs. *J. Evol. Biol.* **30**, 174–190 (2017).

103. I. P. F. Owens, A. Dixon, T. Burke, D. B. A. Thompson, Strategic paternity assurance in the sex-role reversed Eurasian dotterel ( *Charadrius morinellus* ): behavioral and genetic evidence. *Behav. Ecol.* **6**, 14–21 (1995).

104. I. G. McLean, S. D. Kayes, J. O. Murie, L. S. Davis, D. M. Lambert, Genetic monogamy mirrors social monogamy in the Fiordland crested penguin. *N. Z. J. Zool.* **27**, 311–316 (2000).

105. C. C. St Clair, J. R. Waas, R. C. St Clair, P. T. Boag, Unfit mothers? Maternal infanticide in royal penguins. *Anim. Behav.* **50**, 1177–1185 (1995).

106. R. Edler, T. W. P. Friedl, Within-pair young are more immunocompetent than extrapair young in mixed-paternity broods of the red bishop. *Anim. Behav.* **75**, 391–401 (2008).

107. T. W. P. Friedl, G. M. Klump, Determinants of male mating success in the red bishop ( Euplectes orix ). *Behav. Ecol. Sociobiol.* **46**, 387–399 (1999).

108. I. G. Warkentin, A. D.-Curzon, R. E. Carter, J. H. Wetton, P. C. James, L. W. Oliphant, D. T. Parkin, No evidence for extrapair fertilizations in the merlin revealed by DNA fingerprinting. *Mol. Ecol.* **3**, 229–234 (1994).

109. I. Swatschek, D. Ristow, W. Scharlau, C. Wink, M. Wink, Populationsgenetik und Vaterschaftsanalyse beim Eleonorenfalken (Falco eleonorae). *J. Ornithol.* **134**, 137–143 (1993).

110. M. Alcaide, J. J. Negro, D. Serrano, J. L. Tella, C. Rodríguez, Extra-pair paternity in the Lesser Kestrel Falco naumanni: a re-evaluation using microsatellite markers: Extra-pair paternity in the Lesser Kestrel. *Ibis*. **147**, 608–611 (2005).

111. M. Nesje, K. H. Roed, J. T. Lifjeld, P. Lindberg, O. F. Steen, Genetic relationships in the peregrine falcon (Falco peregrinus) analysed by microsatellite DNA markers. *Mol. Ecol.* **9**, 53–60 (2000).

112. M. Villarroel, D. M. Bird, U. Kuhnlein, Copulatory behaviour and paternity in the American kestrel: the adaptive significance of frequent copulations. *Anim. Behav.* **56**, 289–299 (1998).

113. E. Korpimäki, K. Lahti, C. A. May, D. T. Parkin, G. B. Powell, P. Tolonen, J. H. Wetton, Copulatory behaviour and paternity determined by DNA fingerprinting in kestrels: effects of cyclic food abundance. *Anim. Behav.* **51**, 945–955 (1996).

114. M. Krist, P. Munclinger, Superiority of extra-pair offspring: maternal but not genetic effects as revealed by a mixed cross-fostering design. *Mol. Ecol.* **20**, 5074–5091 (2011).

115. B. C. Sheldon, H. Ellegren, Sexual selection resulting from extrapair paternity in collared flycatchers. *Anim. Behav.* **57**, 285–298 (1999).

116. M. Krist, P. Nádvorník, L. Uvírová, S. Bureš, Paternity covaries with laying and hatching order in the collared flycatcher Ficedula albicollis. *Behav. Ecol. Sociobiol.* **59**, 6–11 (2005).

117. B. Rosivall, E. Szöllősi, D. Hasselquist, J. Török, Effects of extrapair paternity and sex on nestling growth and condition in the collared flycatcher, Ficedula albicollis. *Anim. Behav.* **77**, 611–617 (2009).

118. T. Veen, T. Borge, S. C. Griffith, G. P. Saetre, S. Bures, L. Gustafsson, B. C. Sheldon, Hybridization and adaptive mate choice in flycatchers. *Nature*. **411**, 45–49 (2001).

119. T. Wilk, M. Cichoń, K. Wolff, Lack of Evidence for Improved Immune Response of Extra-Pair Nestlings in Collared Flycatcher Ficedula albicollis. *J. Avian Biol.* **39**, 546–552 (2008).

120. J. Moreno, J. G. Martínez, S. González-Braojos, R. Ruiz-de-Castañeda, A. Cantarero, A. Sánchez-Tójar, Extra-pair matings, context-dependence and offspring quality: a brood manipulation experiment in pied flycatchers. *Behaviour*. **150**, 1–22 (2013).

121. T. Lubjuhn, W. Winkel, J. T. Epplen, J. Brün, Reproductive success of monogamous and polygynous pied flycatchers ( Ficedula hypoleuca ). *Behav. Ecol. Sociobiol.* **48**, 12–17 (2000).

122. J. T. Lifjeld, T. Slagsvold, H. M. Lampe, Low frequency of extra-pair paternity in Pied Flycatchers revealed by DNA fingerprinting. *Behav. Ecol. Sociobiol.* **29**, 95–101 (1991).

123. J. Brün, W. Winkel, J. T. Epplen, T. Lubjuhn, Elternschaftsnachweise bei TrauerschnäppernFicedula hypoleuca am Westrand ihres mitteleuropäischen Verbreitungsareals. *J. Ornithol.* **137**, 435–446 (1996).

124. D. Canal, R. Jovani, J. Potti, Male decisions or female accessibility? Spatiotemporal patterns of extra pair paternity in a songbird. *Behav. Ecol.* **23**, 1146–1153 (2012).

125. P. K. Lehtonen, C. R. Primmer, T. Laaksonen, Different traits affect gain of extrapair paternity and loss of paternity in the pied flycatcher, Ficedula hypoleuca. *Anim. Behav.* **77**, 1103–1110 (2009).

126. H. Ellegren, J. T. Lifjeld, T. Slagsvold, C. R. Primmer, Handicapped males and extrapair paternity in pied flycatchers: a study using microsatellite markers. *Mol. Ecol.* **4**, 739–744 (1995).

127. H. P. Gelter, H. Tegelström, High frequency of extra-pair paternity in Swedish pied flycatchers revealed by allozyme electrophoresis and DNA fingerprinting. *Behav. Ecol. Sociobiol.* **31**, 1–7 (1992).

128. J. Moreno, J.-G. MartÃ­nez, J. Morales, E. Lobato, S. Merino, G. TomÃ¡s, R. A. VÃ¡squez, E. MÃ¶stl, J. L. Osorno, Paternity Loss in Relation to Male Age, Territorial Behaviour and Stress in the Pied Flycatcher: Cuckoldry and Male Behaviour. *Ethology*. **116**, 76–84 (2010).

129. J. Moreno, J. G. Martínez, S. González-Braojos, A. Cantarero, R. Ruiz-de-Castañeda, M. Precioso, J. López-Arrabé, Extra-Pair Paternity Declines with Female Age and Wing Length in the Pied Flycatcher. *Ethology*. **121**, 501–512 (2015).

130. O. Rätti, M. Hovi, A. Lundberg, H. Tegelström, R. V. Alatalo, Extra-Pair Paternity and male characteristics in the pied flycatcher. *Behav. Ecol. Sociobiol.* **37**, 419–425 (1995).

131. O. Rätti, A. Lundberg, H. Tegelström, R. V. Alatalo, No evidence for effects of breeding density and male removal on extrapair paternity in the pied flycatcher. *The Auk*. **118**, 147–155 (2001).

132. B. M. Tomotani, E. Caglar, I. de la Hera, A. C. Mateman, M. E. Visser, Early arrival is not associated with more extra-pair fertilizations in a long-distance migratory bird. *J. Avian Biol.* **48**, 854–861 (2017).

133. J. Mitrus, C. Mitrus, R. Rutkowski, M. Sikora, Extra-pair Paternity in Relation to Age of the Red-breasted Flycatcher *Ficedula Parva* males. *Avian Biol. Res.* **7**, 111–116 (2014).

134. M. E, Y. Gong, J. Yu, S. Zhang, Q. Fan, Y. Jiang, H. Wang, Low level of extra-pair paternity between nearest neighbors results from female preference for high-quality males in the yellow-rumped flycatcher (Ficedula zanthopygia). *PLOS ONE*. **12**, e0172713 (2017).

135. T. Anker-Nilssen, O. Kleven, T. Aarvak, J. T. Lifjeld, No evidence of extra-pair paternity in the Atlantic Puffin Fratercula arctica: Paternity in Atlantic Puffins. *Ibis*. **150**, 619–622 (2008).

136. D. C. Dearborn, Sexual dimorphism, extrapair fertilizations, and operational sex ratio in great frigatebirds (Fregata minor). *Behav. Ecol.* **12**, 746–752 (2001).

137. F. A. Juola, D. C. Dearborn, Sequence-based evidence for major histocompatibility complex-disassortative mating in a colonial seabird. *Proc. R. Soc. B Biol. Sci.* **279**, 153–162 (2012).

138. B. C. Sheldon, T. Burke, Copulation behavior and paternity in the chaffinch. *Behav. Ecol. Sociobiol.* **34**, 149–156 (1994).

139. F. M. Hunter, ˜, S. E. Watts, Frequent copulation as a method of paternity assutance in the Northern Fulmar. *Anim. Behav.* **44**, 149–156 (1992).

140. S. B. McRae, T. Burke, Intraspecific brood parasitism in the moorhen: parentage and parasite-host relationships determined by DNA fingerprinting. *Behav. Ecol. Sociobiol.* **38**, 115–129 (1996).

141. W. H. Piper, D. C. Evers, M. W. Meyer, K. B. Tischler, J. D. Kaplan, R. C. Fleischer, Genetic monogamy in the common loon (Gavia immer). *Behav. Ecol. Sociobiol.* **41**, 25–31 (1997).

142. P. R. Grant, B. R. Grant, Causes of lifetime fitness of Darwin’s finches in a fluctuating environment. *Proc. Natl. Acad. Sci.* **108**, 674–679 (2011).

143. B. Abroe, J. C. Garvin, M. C. Pedersen, L. A. Whittingham, P. O. Dunn, D. B. Lank, Brood Sex Ratios Are Related to Male Size but Not to Attractiveness in Common Yellowthroats (Geothlypis trichas) (El Cociente de Sexos en las Nidadas Está Relacionado con el Tamaño de los Machos pero no con el Atractivo en Geothlypis trichas). *The Auk*. **124**, 176–184 (2007).

144. M. L. Hall, R. D. Magrath, Duetting and mate-guarding in Australian magpie-larks (Grallina cyanoleuca). *Behav. Ecol. Sociobiol.* **47**, 180–187 (2000).

145. M. A. Hayes, H. B. Britten, J. A. Barzen, Extra-pair fertilizations in sandhill cranes revealed using microsatellite DNA markers. *The Condor*. **108**, 970 (2006).

146. P. Le Gouar, J. Sulawa, S. Henriquet, C. Tessier, F. Sarrazin, Low evidence for extra-pair fertilizations in two reintroduced populations of Griffon Vulture (Gyps fulvus). *J. Ornithol.* **152**, 359–364 (2011).

147. I. Chiver, B. J. M. Stutchbury, E. S. Morton, The function of seasonal song in a tropical resident species, the Red-throated Ant-tanager (Habia fuscicauda). *J. Ornithol.* **156**, 55–63 (2015).

148. D. Heg, B. J. Ens, T. Burke, L. Jenkins, J. P. Kruijt, Why does the typically monogamous Oystercatcher (Haematopus ostralegus) engage in extra-pair copulations? *Behaviour*. **126**, 247–289 (1993).

149. M. J. L. Magrath, M. A. Elgar, Paternal care declines with increased opportunity for extra-pair matings in fairy martins. *Proc. R. Soc. B Biol. Sci.* **264**, 1731–1736 (1997).

150. N. Saino, C. R. Primmer, H. Ellegren, A. P. Moller, Breeding synchrony and paternity in the barn swallow (Hirundo rustica). *Behav. Ecol. Sociobiol.* **45**, 211–218 (1999).

151. J. T. Lifjeld, O. Kleven, F. Jacobsen, K. J. McGraw, R. J. Safran, R. J. Robertson, Age before beauty? Relationships between fertilization success and age-dependent ornaments in barn swallows. *Behav. Ecol. Sociobiol.* **65**, 1687–1697 (2011).

152. O. Kleven, F. Jacobsen, R. J. Robertson, J. T. Lifjeld, Extrapair mating between relatives in the barn swallow: a role for kin selection? *Biol. Lett.* **1**, 389–392 (2005).

153. A. P. Møller, H. Tegelström, Extra-pair paternity and tail ornamentation in the barn swallow Hirundo rustica. *Behav. Ecol. Sociobiol.* **41**, 353–360 (1997).

154. T. Laskemoen, T. Albrecht, A. Bonisoli-Alquati, J. Cepak, F. de Lope, I. G. Hermosell, L. E. Johannessen, O. Kleven, A. Marzal, T. A. Mousseau, A. P. Møller, R. J. Robertson, G. Rudolfsen, N. Saino, Y. Vortman, J. T. Lifjeld, Variation in sperm morphometry and sperm competition among barn swallow (Hirundo rustica) populations. *Behav. Ecol. Sociobiol.* **67**, 301–309 (2013).

155. H. Ellegren, G. Lindgren, C. R. Primmer, A. P. Moller, Fitness loss and germline mutations in barn swallows breeding in Chernobyl. *Nature*. **389**, 593–596 (1997).

156. C. Eikenaar, M. Whitham, J. Komdeur, M. van der Velde, I. T. Moore, Testosterone, plumage colouration and extra-pair paternity in male North-American barn swallows. *PLoS ONE*. **6**, e23288 (2011).

157. M. Hasegawa, E. Arai, W. Kojima, W. Kitamura, G. Fujita, H. Higuchi, M. Watanabe, M. Nakamura, Low level of extra-pair paternity in a population of the barn swallow Hirundo Rustica Gutturalis. *Ornithol. Sci.* **9**, 161–164 (2010).

158. W. Kojima, W. Kitamura, S. Kitajima, Y. Ito, K. Ueda, G. Fujita, H. Higuchi, Female Barn Swallows Gain Indirect but not Direct Benefits through Social Mate Choice. *Ethology*. **115**, 939–947 (2009).

159. A. P. Moller, Extrapair paternity in relation to sexual ornamentation, arrival date, and condition in a migratory bird. *Behav. Ecol.* **14**, 707–712 (2003).

160. C. R. Neuman, R. J. Safran, I. J. Lovette, Male tail streamer length does not predict apparent or genetic reproductive success in North American barn swallows Hirundo rustica erythrogaster. *J. Avian Biol.* **38**, 28–36 (2007).

161. H. G. Smith, Sexual selection and the tail ornaments of North American barn swallows. *Behav. Ecol. Sociobiol.* **28**, 195–201 (1991).

162. Y. Vortman, A. Lotem, R. Dor, I. J. Lovette, R. J. Safran, The sexual signals of the East-Mediterranean barn swallow: a different swallow tale. *Behav. Ecol.* **22**, 1344–1352 (2011).

163. M. L. Evans, B. J. M. Stutchbury, B. E. Woolfenden, Off-territory forays and genetic mating system of the wood thrush Hylocichla mustelina. *The Auk*. **125**, 67–75 (2008).

164. S. J. Triggs, M. J. Williams, S. J. Marshall, G. K. Chambers, Genetic relationships within a population of Blue Duck Hymenolaimus malacorhynchos. *Wildfowl*. **42**, 87–93 (1991).

165. C. I. Miño, I. L. Pollet, C. A. Bishop, M. A. Russello, Genetic mating system and population history of the endangered Western Yellow-breasted Chat ( *Icteria virens auricollis* ) in British Columbia, Canada. *Can. J. Zool.* **89**, 881–891 (2011).

166. D. S. Richardson, T. Burke, Extra-pair paternity in relation to male age in Bullock’s orioles. *Mol. Ecol.* **8**, 2115–2126 (1999).

167. I. F. Lopes, C. I. Miño, C. D. Rocha, D. M. M. Oliveira, S. N. Del Lama, Inferred kinship patterns reveal low levels of extra-pair paternity in the endangered Neotropical Jabiru Stork (Jabiru mycteria, Aves: Ciconiiformes). *Genetica*. **141**, 195–203 (2013).

168. J. W. Atwell, G. C. Cardoso, D. J. Whittaker, T. D. Price, E. D. Ketterson, Hormonal, Behavioral, and Life-History Traits Exhibit Correlated Shifts in Relation to Population Establishment in a Novel Environment. *Am. Nat.* **184**, E147–E160 (2014).

169. N. M. Gerlach, J. W. McGlothlin, P. G. Parker, E. D. Ketterson, Promiscuous mating produces offspring with higher lifetime fitness. *Proc. R. Soc. B Biol. Sci.* **279**, 860–866 (2012).

170. N. M. Gerlach, E. D. Ketterson, Experimental elevation of testosterone lowers fitness in female dark-eyed juncos. *Horm. Behav.* **63**, 782–790 (2013).

171. M. Wink, D. Becker, D. Tolkmitt, V. Knigge, H. Sauer-Gürth, H. Staudter, Mating system, paternity and sex allocation in Eurasian Wrynecks (Jynx torquilla). *J. Ornithol.* **152**, 983–989 (2011).

172. J. R. Freeland, S. J. Hannon, G. Dobush, P. T. Boag, Extra-pair paternity in willow ptarmigan broods: measuring costs of polygyny to males. *Behav. Ecol. Sociobiol.* **36**, 349–355 (1995).

173. D. P. Benson, Low Extra-Pair Paternity in White-Tailed Ptarmigan. *The Condor*. **104**, 192–197 (2002).

174. I. M. van den Heuvel, M. I. Cherry, G. M. Klump, Crimson-breasted Shrike females with extra pair offspring contributed more to duets. *Behav. Ecol. Sociobiol.* **68**, 1245–1252 (2014).

175. S. Yamagishi, Isao Nishiumi, Chikashi Shimoda, Extrapair fertilization in monogamous bull-headed shrikes revealed by DNA fingerprinting. *The Auk*. **109**, 711–721 (1992).

176. M. A. Etterson, Parentage in an Oklahoma population of loggerhead shrikes assessed using nuclear microsatellites. *The Condor*. **106**, 401–404 (2004).

177. F. Valera, Male shrikes punish unfaithful females. *Behav. Ecol.* **14**, 403–408 (2003).

178. M. Bukacińska, D. Bukaciński, J. T. Epplen, K. P. Sauer, T. Lubjuhn, Low frequency of extra-pair paternity in Common Gulls (Larus canus) as revealed by DNA fingerprinting. *J. Für Ornithol.* **139**, 413–420 (1998).

179. L. Gilbert, T. Burke, A. Krupa, No evidence for extra-pair paternity in the western gull. *Mol. Ecol.* **7**, 1549–1552 (1998).

180. R. Ležalová-Piálková, Molecular evidence for extra-pair paternity and intraspecific brood parasitism in the Black-headed Gull. *J. Ornithol.* **152**, 291–295 (2011).

181. J. G. Ewen, K. L. Ciborowski, R. H. Clarke, R. L. Boulton, M. F. Clarke, Evidence of extra-pair paternity in two socially monogamous Australian passerines: the Crescent Honeyeater and the Yellow-faced Honeyeater. *Emu*. **108**, 133 (2008).

182. J. Neto, B. Hansson, D. Hasselquist, Low frequency of extra-pair paternity in Savi’s Warblers (Locustella luscinioides). *Behaviour*. **147**, 1413–1429 (2010).

183. O. Kleven, B.-A. Bjerke, J. T. Lifjeld, Genetic monogamy in the Common Crossbill (Loxia curvirostra). *J. Ornithol.* **149**, 651–654 (2008).

184. R. C. Fleischer, C. L. Tarr, T. K. Pratt, Genetic structure and mating system in the palila, an endangered Hawaiian honeycreeper, as assessed by DNA-fingerprinting. *Mol. Ecol.* **3**, 383–392 (1994).

185. C. Landgraf, K. Wilhelm, J. Wirth, M. Weiss, S. Kipper, Affairs happen—to whom? A study on extrapair paternity in common nightingales. *Curr. Zool.* **63**, 421–431 (2017).

186. A. Johnsen, J. T. Lifjeld, Ecological constraints on extra-pair paternity in the bluethroat. *Oecologia*. **136**, 476–483 (2003).

187. A. Johnsen, K. L. Carter, K. Delhey, J. T. Lifjeld, R. J. Robertson, B. Kempenaers, Laying-order effects on sperm numbers and on paternity: comparing three passerine birds with different life histories. *Behav. Ecol. Sociobiol.* **66**, 181–190 (2012).

188. S. Questiau, M.-C. Eybert, P. Taberlet, Amplified fragment length polymorphism (AFLP) markers reveal extra-pair parentage in a bird species: the bluethroat (Luscinia svecica). *Mol. Ecol.* **8**, 1331–1339 (1999).

189. S. G. Lawless, G. Ritchison, P. H. Klatt, D. F. Westneat, The Mating Strategies of Eastern Screech-Owls: A Genetic Analysis. *The Condor*. **99**, 213–217 (1997).

190. B. J. Olsen, R. Greenberg, R. C. Fleischer, J. R. Walters, Extrapair paternity in the swamp sparrow, Melospiza georgiana: male access or female preference? *Behav. Ecol. Sociobiol.* **63**, 285–294 (2008).

191. C. E. Hill, C. Akcay, S. E. Campbell, M. D. Beecher, Extrapair paternity, song, and genetic quality in song sparrows. *Behav. Ecol.* **22**, 73–81 (2011).

192. D. L. Major, C. A. Barber, Extra-pair paternity in first and second broods of eastern Song Sparrows. *J. Field Ornithol.* **75**, 152–156 (2004).

193. J. M. Reid, L. F. Keller, A. B. Marr, P. Nietlisbach, R. J. Sardell, P. Arcese, Pedigree error due to extra-pair reproduction substantially biases estimates of inbreeding depression: pedigree error and inbreeding depression. *Evolution*. **68**, 802–815 (2014).

194. I. R. Hartley, M. Shepherd, T. Robson, T. Burke, Reproductive success of polygynous male corn buntings (Miliaria calandra) as confirmed by DNA fingerprinting. *Behav Ecol*. **4**, 310–317 (1993).

195. A. G. Da Silva, J. R. Eberhard, T. F. Wright, M. L. Avery, M. A. Russello, Genetic evidence for high propagule pressure and long-distance dispersal in monk parakeet (Myiopsitta monachus) invasive populations. *Mol. Ecol.* **19**, 3336–3350 (2010).

196. R. Zilberman, B. Moav, Y. Yom-Tov, Extra-pair paternity in the socially monogamous orange-tufted sunbird (Nectarinia Osea Osea). *Israel Journal of Zoology*. **45**, 407–421 (1999).

197. P. Brekke, P. Cassey, C. Ariani, J. G. Ewen, Evolution of extreme-mating behaviour: patterns of extrapair paternity in a species with forced extrapair copulation. *Behav. Ecol. Sociobiol.* **67**, 963–972 (2013).

198. I. Castro, K. M. Mason, D. P. Armstrong, D. M. Lambert, Effect of Extra-Pair Paternity on Effective Population Size in a Reintroduced Population of the Endangered Hihi, and Potential for Behavioural Management. *Conserv. Genet.* **5**, 381–393 (2004).

199. J. G. Ewen, D. P. Armstrong, D. M. Lambert, Floater males gain reproductive success through extrapair feritilizations in the stichbird. *Anim. Behav.* **58**, 321–328 (1999).

200. P. Quillfeldt, T. Schmoll, H.-U. Peter, J. T. Epplen, T. Lubjuhn, Genetic Monogamy in Wilson’s Storm-Petrel. *The Auk*. **118**, 242 (2001).

201. R. A. Mauck, T. A. Waite, P. G. Parker, Monogamy in Leach’s Storm-Petrel: DNA-Fingerprinting Evidence. *The Auk*. **112**, 473–482 (1995).

202. D. R. Currie, T. Burke, R. L. Whitney, D. B. A. Thompson, Male and female behaviour and extra-pair paternity in the wheatear. *Anim. Behav.* **55**, 689–703 (1998).

203. D. Currie, A. P. Krupa, T. Burke, D. B. A. Thompson, The effect of experimental male removals on extrapair paternity in the wheatear,Oenanthe oenanthe. *Anim. Behav.* **57**, 145–152 (1999).

204. D. Kudernatsch, M. Buchmann, W. Fiedler, G. Segelbacher, Extrapair paternity in a German population of the Northern Wheatear (Oenanthe oenanthe). *J. Ornithol.* **151**, 491–498 (2010).

205. Y.-C. Hsu, S.-H. Li, Y.-S. Lin, M. T. Philippart, L. L. Severinghaus, High Frequency of Extra-Pair Copulation with Low Level of Extra-Pair Fertilization in the Lanyu Scops Owl Otus elegans botelensis. *J. Avian Biol.* **37**, 36–40 (2006).

206. D. P. Arsenault, P. B. Stacey, G. A. Hoelzer, No Extra-Pair Fertilization in Flammulated Owls despite Aggregated Nesting. *The Condor*. **104**, 197–202 (2002).

207. W. F. D. van Dongen, R. A. Mulder, Multiple ornamentation, female breeding synchrony, and extra-pair mating success of golden whistlers (Pachycephala pectoralis). *J. Ornithol.* **150**, 607–620 (2009).

208. P. Quillfeldt, J. F. Masello, G. Segelbacher, Extra-pair paternity in seabirds: a review and case study of Thin-billed Prions Pachyptila belcheri. *J. Ornithol.* **153**, 367–373 (2012).

209. H. Hoi, M. HoiLeitner, An alternative route to coloniality in the bearded tit: Females pursue extra-pair fertilizations. *Behav. Ecol.* **8**, 113–119 (1997).

210. J.-W. Lee, M.-S. Kim, T. Burke, B. J. Hatchwell, Extrapair paternity in a flock-living passerine, the vinous-throated parrotbill *Paradoxornis webbianus*. *J. Avian Biol.* **40**, 469–474 (2009).

211. V. Dietrich, T. Schmoll, W. Winkel, J. T. Epplen, T. Lubjuhn, Pair identity - an important factor concerning variation in extra-pair paternity in the coal tit (Parus ater). *Behaviour*. **141**, 817–835 (2004).

212. T. Lubjuhn, T. Gerken, J. Brün, J. T. Epplen, High Frequency of Extra-Pair Paternity in the Coal Tit. *J. Avian Biol.* **30**, 229–233 (1999).

213. D. J. Mennill, S. M. Ramsay, P. T. Boag, L. M. Ratcliffe, Patterns of extrapair mating in relation to male dominance status and female nest placement in black-capped chickadees. *Behav. Ecol.* **15**, 757–765 (2004).

214. K. Otter, L. Ratcliffe, D. Michaud, P. T. Boag, Do female black-capped chickadees prefer high-ranking males as extra-pair partners? *Behav. Ecol. Sociobiol.* **43**, 25–36 (1998).

215. A. Charmantier, C. Blondel, A contrast in extra-pair paternity levels on mainland and island populations of mediterranean blue tits. *Ethology*. **109**, 351–363 (2003).

216. A. Charmantier, J. Blondel, P. Perret, M. M. Lambrechts, Do extra-pair paternities provide genetic benefits for female bue tits Parus caeruleus? *J. Avian Biol.* **35**, 524–532 (2004).

217. B. de Jong, L. Lens, M. van der Velde, P. Korsten, T. Groothuis, J. Komdeur, Testosterone Reduces Promiscuity of Female Blue Tits (*Cyanistes caeruleus*): An Experimental Study. *Ethology*. **123**, 69–82 (2017).

218. A. Dreiss, M. Richard, F. Moyen, J. White, A. P. Møller, E. Danchin, Sex ratio and male sexual characters in a population of blue tits, Parus caeruleus. *Behav. Ecol.* **17**, 13–19 (2006).

219. K. Foerster, K. Delhey, A. Johnsen, J. T. Lifjeld, B. Kempenaers, Females increase offspring heterozygosity and fitness through extra-pair matings. *Nature*. **425**, 714–717 (2003).

220. A. Gullberg, H. Tegelstrom, H. P. Gelter, DNA fingerprinting reveals multiple paternity in families of Great and Blue Tits (Parus major and P. caeruleus). *Hereditas*. **117**, 103–108 (1992).

221. L. E. Johannessen, T. Slagsvold, B. T. Hansen, J. T. Lifjeld, Manipulation of male quality in wild tits: effects on paternity loss. *Behav. Ecol.* **16**, 747–754 (2005).

222. B. Kempenaers, G. R. Verheyen, A. A. Dhondi, Extrapair paternity in the blue tit (Parus caeruleus) : female choice, male characteristics, and offspring quality. *Behav. Ecol.* **8**, 481–492 (1997).

223. C. Krokene, K. Rigstad, M. Dale, J. T. Lifjeld, The function of extrapair paternity in blue tits and great tits: good genes or fertility insurance? *Behav. Ecol.* **9**, 649–656 (1998).

224. D. I. Leech, No effect of parental quality or extrapair paternity on brood sex ratio in the blue tit (Parus caeruleus). *Behav. Ecol.* **12**, 674–680 (2001).

225. M. J. L. Magrath, O. Vedder, M. van der Velde, J. Komdeur, Maternal Effects Contribute to the Superior Performance of Extra-Pair Offspring. *Curr. Biol.* **19**, 792–797 (2009).

226. L. Schlicht, M. Valcu, B. Kempenaers, Spatial patterns of extra-pair paternity: beyond paternity gains and losses. *J. Anim. Ecol.* **84**, 518–531 (2015).

227. L. Schlicht, A. Girg, P. Loës, M. Valcu, B. Kempenaers, Male extrapair nestlings fledge first. *Anim. Behav.* **83**, 1335–1343 (2012).

228. O. Vedder, M. J. L. Magrath, A. M. F. Harts, E. Schut, M. van der Velde, J. Komdeur, Reduced extrapair paternity in response to experimental stimulation of earlier incubation onset in blue tits. *Behav. Ecol.* **21**, 9–15 (2010).

229. L. Lens, S. Van Dongen, M. Van den Broeck, C. Van Broeckhoven, A. A. Dhondt, Why female crested tits copulate repeatedly with the same partner: evidence for the mate assessment hypothesis. *Behav. Ecol.* **8**, 87–91 (1997).

230. Y. G. Araya-Ajoy, S. Kuhn, K. J. Mathot, A. Mouchet, A. Mutzel, M. Nicolaus, J. J. Wijmenga, B. Kempenaers, N. J. Dingemanse, Sources of (co)variation in alternative siring routes available to male great tits ( *Parus major* ). *Evolution*. **70**, 2308–2321 (2016).

231. V. Bókony, I. Pipoly, K. Szabó, B. Preiszner, E. Vincze, S. Papp, G. Seress, T. Hammer, A. Liker, Innovative females are more promiscuous in great tits (Parus major). *Behav. Ecol.* **28**, 579–588 (2017).

232. V. García-Navas, E. S. Ferrer, C. Cáliz-Campal, J. Bueno-Enciso, R. Barrientos, J. J. Sanz, J. Ortego, Spatiotemporal and genetic contingency of extrapair behaviour in a songbird. *Anim. Behav.* **106**, 157–169 (2015).

233. K. M. Kawano, N. Yamaguchi, E. Kasuya, T. Yahara, Extra-pair mate choice in the female great tit Parus major: good males or compatible males. *J. Ethol.* **27**, 349–359 (2009).

234. T. Lubjuhn, T. Gerken, J. Brün, T. Schmoll, Yearling male great tits, Parus major, suffer more strongly from cuckoldry than older males. *Zoology*. **110**, 387–397 (2007).

235. K. A. Otter, I. R. K. Stewart, P. K. McGregor, A. M. R. Terry, T. Dabelsteen, T. Burke, Extra-pair paternity among Great Tits Parus major following manipulation of male signals. *J. Avian Biol.* **32**, 338–344 (2001).

236. S. C. Patrick, J. R. Chapman, H. L. Dugdale, J. L. Quinn, B. C. Sheldon, Promiscuity, paternity and personality in the great tit. *Proc. R. Soc. B Biol. Sci.* **279**, 1724–1730 (2012).

237. S. Strohbach, E. Curio, A. Bathen, J. T. Epplen, T. Lubjuhn, Extrapair paternity in the great tit (Parus major): a test of the “good genes” hypothesis. *Behav. Ecol.* **9**, 388–396 (1998).

238. K. van Oers, P. J. Drent, N. J. Dingemanse, B. Kempenaers, Personality is associated with extrapair paternity in great tits, Parus major. *Anim. Behav.* **76**, 555–563 (2008).

239. N. Verboven, C. Mateman, Low frequency of extra-pair fertilizations in the great tit Parus major revealed by DNA fingerprinting. *J. Avian Biol.* **28**, 231–239 (1997).

240. W. Winkel, D. Winkel, T. Lubjuhn, Paternity analysis of four Great Tit (Parus major) broods in unusually close proximity. *J. Ornithol.* **142**, 429–432 (2001).

241. T. Yuta, I. Koizumi, Does nest predation risk affect the frequency of extra-pair paternity in a socially monogamous passerine? *J. Avian Biol.* **47**, 153–158 (2016).

242. S. Lampila, M. Orell, L. Kvist, Willow tit Parus montanus extrapair offspring are more heterozygous than their maternal half-siblings. *J. Avian Biol.* **42**, 355–362 (2011).

243. M. Orell, S. Rytkonen, V. Launonen, P. Welling, K. Korvula, K. Kumpulainen, L. Bachmann, Low frequency extra-pair paternity in the Willow Tit Parus montanus as revealed by DNA fingerprinting. *Ibis*. **139**, 562–566 (1997).

244. E. Garcia-Del-Rey, O. Kleven, J. T. Lifjeld, Extrapair paternity in insular African Blue Tits *Cyanistes teneriffae* is no less frequent than in continental Eurasian Blue Tits *Cyanistes caeruleus*. *Ibis*. **154**, 862–867 (2012).

245. J. Ju, J. Yin, P. Racey, L. Zhang, D. Li, D. Wan, Extra-Pair Paternity in Varied Tits *Poecile varius*. *Acta Ornithol.* **49**, 131–137 (2014).

246. C. Bichet, D. J. Penn, Y. Moodley, L. Dunoyer, E. Cellier-Holzem, M. Belvalette, A. Grégoire, S. Garnier, G. Sorci, Females tend to prefer genetically similar mates in an island population of house sparrows. *BMC Evol. Biol.* **14**, 47 (2014).

247. P. J. Cordero, J. H. Wetton, D. T. Parkin, Extra-pair paternity and male badge size in the House Sparrow. *J. Avian Biol.* **30**, 97–102 (1999).

248. C. Edly-Wright, P. L. Schwagmeyer, P. G. Parker, D. W. Mock, Genetic similarity of mates, offspring health and extrapair fertilization in house sparrows. *Anim. Behav.* **73**, 367–378 (2007).

249. S. C. Griffith, I. R. K. Stewart, D. A. Dawson, I. P. F. Owens, T. Burke, Contrasting levels of extra-pair paternity in mainland and island populations of the house sparrow (Passer domesticus): is there an ?island effect?? *Biol. J. Linn. Soc.* **68**, 303–316 (1999).

250. Y.-H. Hsu, J. Schroeder, I. Winney, T. Burke, S. Nakagawa, Are extra-pair males different from cuckolded males? A case study and a meta-analytic examination. *Mol. Ecol.* **24**, 1558–1571 (2015).

251. I. R. K. Stewart, R. D. Hanschu, T. Burke, D. F. Westneat, Tests of Ecological, Phenotypic, and Genetic Correlates of Extra-Pair Paternity in the House Sparrow. *The Condor*. **108**, 399–413 (2006).

252. I. R. K. Stewart, D. F. Westneat, R. L. Barrick, Food supplementation and extrapair paternity in house sparrows. *Am. Midl. Nat.* **174**, 278–289 (2015).

253. R. Václav, H. Hoi, Experimental manipulation of timing of breeding suggests laying order instead of breeding synchrony affects extra-pair paternity in house sparrows. *J. Ornithol.* **148**, 395–400 (2007).

254. R. Vaclav, H. Hoi, D. Blomqvist, Food supplementation affects extrapair paternity in house sparrows (Passer domesticus). *Behav. Ecol.* **14**, 730–735 (2003).

255. J. P. Veiga, L. Boto, Low frequency of extra-pair fertilisations in House Sparrows breeding at high density. *J. Avian Biol.* **31**, 237–244 (2000).

256. J. H. Wetton, D. T. Parkin, An association between fertility and cuckoldry in the House Sparrow, Passer domesticus. *Proc. R. Soc. Lond. Ser. B-Biol. Sci.* **245**, 227–23 (1991).

257. R. R. Whitekiller, D. F. Westneat, P. L. Schwagmeyer, D. W. Mock, Badge size and extra-pair fertilizations in the house sparrow. *The Condor*. **102**, 342–348 (2000).

258. P. J. Cordero, P. Heeb, J. H. Wetton, D. T. Parkin, Extra-pair fertilizations in Tree Sparrows Passer montanus: Extra-pair fertilizations in Tree Sparrows. *Ibis*. **144**, E67–E72 (2002).

259. G. Seress, K. Szabó, D. Nagy, A. Liker, Extra-pair paternity of tree sparrow (Passer Montanus ) in a semi-urban population. *TISCIA*, 17–21 (2007).

260. C. R. Freeman-Gallant, DNA fingerprinting reveals female preference for male parental care in Savannah sparrows. *Proc. R. Soc. Lond. Ser. B-Biol. Sci.* **263**, 157–160 (1996).

261. C. R. Freeman-Gallant, M. Meguerdichian, N. T. Wheelwright, S. V. Sollecito, Social pairing and female mating fidelity predicted by restriction fragment length polymorphism similarity at the major histocompatibility complex in a songbird. *Mol. Ecol.* **12**, 3077–3083 (2003).

262. C. R. Freeman-Gallant, N. T. Wheelwaright, K. E. Meiklejohn, S. L. States, S. V. Sollecito, Little effect of extrapair paternity on the opportunity for sexual selection in savannah sparrows (Passerculus sandwichenisis). *Evolution*. **59**, 422–430 (2005).

263. N. G. Perlut, C. R. Freeman-Gallant, A. M. Strong, T. M. Donovan, C. W. Kilpatrick, N. J. Zalik, Agricultural management affects evolutionary processes in a migratory songbird. *Mol. Ecol.* **17**, 1248–1255 (2008).

264. L. K. Estep, H. Mays, Jr., A. J. Keyser, B. Ballentine, G. E. Hill, Effects of breeding density and plumage coloration on mate guarding and cuckoldry in blue grosbeaks ( *Passerina caerulea* ). *Can. J. Zool.* **83**, 1143–1148 (2005).

265. D. F. Westneat, Genetic parentage in the Indigo Bunting: a study using DNA fingerprinting. *Behav. Ecol. Sociobiol.* **27**, 67–76 (1990).

266. S. L. Ardern, Wei Ma, J. G. Ewen, D. P. Armstrong, D. M. Lambert, Social and Sexual Monogamy in Translocated New Zealand Robin Populations Detected Using Minisatellite DNA. *The Auk*. **114**, 120–126 (1997).

267. S. S. Taylor, S. Boessenkool, I. G. Jamieson, Genetic monogamy in two long-lived New Zealand passerines. *J. Avian Biol.* **39**, 579–583 (2008).

268. D. K. Dowling, R. A. Mulder, Combined influence of maternal and paternal quality on sex allocation in red-capped robins. *J. Evol. Biol.* **19**, 440–449 (2006).

269. E. Nemeth, B. Kempenaers, G. Matessi, H. Brumm, Rock sparrow song reflects male age and seproductive success. *PLoS ONE*. **7**, e43259 (2012).

270. A. Pilastro, M. Griggio, L. Biddau, T. Mingozzi, Extrapair paternity as a cost of polygyny in the rock sparrow: behavioural and genetic evidence of the “trade-off” hypothesis. *Anim. Behav.* **63**, 967–974 (2002).

271. M. Chu, W. D. Koenig, R. C. Fleischer, Social and genetic monogamy in territorial and loosely colonial populations of Phainopepla (Phainopepla nitens). *The Auk*. **119**, 770–777 (2002).

272. J. Graves, R. T. Hay, M. Scallan, S. Rowe, Extra-pair paternity in the shag, *Phalacrocorax aristotelis* as determined by DNA fingerprinting. *J. Zool.* **226**, 399–408 (1992).

273. L. Calderón, W. S. Svagelj, F. Quintana, S. C. Lougheed, P. L. Tubaro, No evidence of extra-pair paternity or intraspecific brood parasitism in the Imperial Shag Phalacrocorax atriceps. *J. Ornithol.* **153**, 399–404 (2012).

274. P. Minias, K. Wojczulanis-Jakubas, R. Rutkowski, K. Kaczmarek, T. Janiszewski, Spatial patterns of extra-pair paternity in a waterbird colony: separating the effects of nesting density and nest site location. *Behav. Ecol. Sociobiol.* **70**, 369–376 (2016).

275. D. Schamel, D. M. Tracy, D. B. Lank, D. F. Westneat, Mate Guarding, Copulation Strategies and Paternity in the Sex-Role Reversed, Socially Polyandrous Red-Necked Phalarope Phalaropus lobatus. *Behav. Ecol. Sociobiol.* **57**, 110–118 (2004).

276. K. P. Huyvaert, D. J. Anderson, T. C. Jones, W. Duan, P. G. Parker, Extra-pair paternity in waved albatrosses. *Mol. Ecol.* **9**, 1415–1419 (2000).

277. K. P. Huyvaert, D. J. Anderson, P. G. Parker, Mate Opportunity Hypothesis and Extrapair Paternity in Waved Albatrosses (Phoebastria irrorata) (La Hipótesis de Oportunidad de Apareamiento y Paternidad Extra-pareja en Phoebastria irrorata). *The Auk*. **123**, 524–536 (2006).

278. C. P. Villavicencio, B. Apfelbeck, W. Goymann, Parental care, loss of paternity and circulating levels of testosterone and corticosterone in a socially monogamous song bird. *Front. Zool.* **11**, 11 (2014).

279. O. Kleven, T. Øigarden, B. E. Foyn, A. Moksnes, E. Røskaft, G. Rudolfsen, B. G. Stokke, J. T. Lifjeld, Low frequency of extrapair paternity in the common redstart (Phoenicurus phoenicurus). *J. Ornithol.* **148**, 373–378 (2007).

280. W. Forstmeier, B. Kempenaers, A. Meyer, B. Leisler, A novel song parameter correlates with extra-pair paternity and reflects male longevity. *Proc. R. Soc. B Biol. Sci.* **269**, 1479–1485 (2002).

281. A. Grendelmeier, R. Arlettaz, J. Olano-Marin, G. Pasinelli, Experimentally provided conspecific cues boost bird territory density but not breeding performance. *Behav. Ecol.* **28**, 174–185 (2017).

282. U. B. Gyllensten, S. Jakobsson, H. Temrin, No evidence for illegitimate young in monogamous and polygynous warblers. *Nature*. **343**, 168–170 (1990).

283. G. Bj›rnstad, J. T. Lifjeld, High frequency of extra-pair paternity in a dense and synchronous population of willow warblers Phylloscopus trochilus. *J. Avian Biol.* **28**, 319–324 (1997).

284. A. K. Fridolfsson, U. B. Gyllensten, S. Jakobsson, Microsatellite markers for paternity testing in the Willow warbler Phylloscopus trochilus: high frequency of extra-pair young in an island population. *hereditas*. **126**, 127–132 (1997).

285. D. Gil, P. J. B. Slater, J. A. Graves, Extra-pair paternity and song characteristics in the willow warbler Phylloscopus trochilus. *J. Avian Biol.* **38**, 291–297 (2007).

286. M.-H. Li, K. Välimäki, M. Piha, T. Pakkala, J. Merilä, Extrapair paternity and maternity in the three-toed woodpecker, Picoides tridactylus: insights from microsatellite-based parentage analysis. *PLoS ONE*. **4**, e7895 (2009).

287. P. Pechacek, Klaus G. Michalek, H. Winkler, D. Blomqvist, Monogamy with exceptions: social and genetic mating system in a bird species with high paternal investment. *Behaviour*. **142**, 1093–1114 (2005).

288. S. B. Smith, J. E. McKay, M. T. Murphy, D. A. Duffield, Spatial patterns of extra-pair paternity for spotted towhees *Pipilo maculatus* in urban parks. *J. Avian Biol.* **47**, 815–823 (2016).

289. P. H. Klatt, B. J. M. Stutchbury, M. L. Evans, Incubation Feeding by Male Scarlet Tanagers: A Mate Removal Experiment. *J. Field Ornithol.* **79**, 1–10 (2008).

290. K. S. Hoset, Y. Espmark, F. Fossøy, B. G. Stokke, H. Jensen, M. I. Wedege, A. Moksnes, Extra-pair paternity in relation to regional and local climate in an Arctic-breeding passerine. *Polar Biol.* **37**, 89–97 (2014).

291. S. Yezerinac, R. B. Lanctot, G. K. Sage, S. L. Talbot, Social and Genetic Mating System of the American Golden-Plover. *The Condor*. **115**, 808–815 (2013).

292. E. P. van Rooij, L. A. Rollins, C. E. Holleley, S. C. Griffith, Extra-pair paternity in the long-tailed finch *Poephila acuticauda*. *PeerJ*. **4**, e1550 (2016).

293. M. Lettink, I. G. Jamieson, C. D. Millar, D. M. Lambert, Mating system and genetic variation in the endangered New Zealand takahe. *Conserv. Genet.* **3**, 427–434 (2002).

294. S. A. Tarof, P. M. Kramer, J. Tautin, B. J. M. Stutchbury, Effects of known age on male paternity in a migratory songbird. *Behav. Ecol.* **23**, 313–321 (2012).

295. R. H. Wagner, M. D. Schug, E. S. Morton, Confidence of paternity, actual paternity and parental effort by purple martins. *Anim. Behav.* **52**, 123–132 (1996).

296. M. L. McFarlane, M. R. Evans, K. A. Feldheim, M. Preault, R. C. K. Bowie, M. I. Cherry, Long tails matter in sugarbirds--positively for extrapair but negatively for within-pair fertilization success. *Behav. Ecol.* **21**, 26–32 (2010).

297. S. J. Wells, W. Ji, J. Dale, B. Jones, D. Gleeson, Male size predicts extrapair paternity in a socially monogamous bird with extreme sexual size dimorphism. *Behav. Ecol.* **26**, 200–206 (2015).

298. J. J. Austin, D. T. Parkin, Low frequency of extra-pair paternity in two colonies of the socially monogamous short-tailed shearwater Puffinus tenuirostris. *Mol. Ecol.* **5**, 145–150 (1996).

299. A. Pilastro, F. Pezzo, S. Olmastroni, C. Callegarin, S. Corsolini, S. Focardi, Extrapair paternity in the Adélie Penguin Pygoscelis adeliae. *Ibis*. **143**, 681–684 (2001).

300. J. Moreno, L. Boto, J. A. Fargallo, A. de Leon, J. Potti, Absence of extra-pair fertilisations in the Chinstrap Penguin Pygoscelis antarctica. *J. Avian Biol.* **31**, 580–583 (2000).

301. M. Dallimer, P. J. Jones, An estimation of the rate of reproductive cheating in the Red-billed Quelea *Quelea quelea*. *Ostrich*. **78**, 637–639 (2007).

302. T. R. Krueger, D. A. Williams, W. A. Searcy, The genetic mating system of a tropical tanager. *The Condor*. **110**, 559–562 (2008).

303. A. D. Ball, R. E. van Dijk, P. Lloyd, Á. Pogány, D. A. Dawson, S. Dorus, R. C. K. Bowie, T. Burke, T. Székely, Levels of extra-pair paternity are associated with parental care in penduline tits (Remizidae). *Ibis*. **159**, 449–455 (2017).

304. J. I. Hoffman, K. Munro, R. M. Kilner, W. Amos, High rates of infidelity in the Grey Fantail *Rhipidura albiscapa* suggest that testis size may be a better correlate of extra-pair paternity than sexual dimorphism. *Ibis*. **152**, 378–385 (2010).

305. M. A. S. Alves, D. M. Bryant, Brood parasitism in the sand martin,Riparia riparia: evidence for two parasitic strategies in a colonial passerine. *Anim. Behav.* **56**, 1323–1331 (1998).

306. J. Augustin, D. Blomqvist, T. Szép, Z. D. Szabó, R. H. Wagner, No evidence of genetic benefits from extra-pair fertilisations in female sand martins (Riparia riparia). *J. Ornithol.* **148**, 189–198 (2007).

307. F. Helfenstein, C. Tirard, E. Danchin, R. H. Wagner, Low Frequency of Extra-Pair Paternity and High Frequency of Adoption in Black-Legged Kittiwakes. *The Condor*. **106**, 149–155 (2004).

308. A. S. Beheler, O. E. Rhodes, Within-season prevalence of extrapair young in broods of double-brooded and mate faithful Eastern Phoebes (Sayornis phoebe) in Indiana. *Auk*. **120**, 1054–1061 (2003).

309. K. F. Conrad, R. J. Robertson, P. T. Boag, Frequency of Extrapair Young Increases in Second Broods of Eastern Phoebes. *The Auk*. **115**, 497–502 (1998).

310. C. Voigt, S. Leitner, M. Gahr, Mate fidelity in a population of Island Canaries (Serinus canaria) in the Madeiran Archipelago. *J. Ornithol.* **144**, 86–92 (2003).

311. M. Hoi-Leitner, H. Hoi, R. Romero-Pujante, F. Valera, Female extra-pair behaviour and environmental quality in the serin (Serinus serinus): a test of the “constrained female hypothesis.” *Proc R Soc Lond B*. **266**, 1021–1026 (1999).

312. P. G. Mota, M. Hoi-Leitner, Intense extrapair behaviour in a semicolonial passerine does not result in extrapair fertilizations. *Anim. Behav.* **66**, 1019–1026 (2003).

313. P. J. Kappes, B. J. M. Stutchbury, B. E. Woolfenden, The relationship between carotenoid-based coloration and pairing, Within- and extra-pair mating success in the American redstart. *The Condor*. **111**, 684–693 (2009).

314. S. Perreault, R. E. Lemon, U. Kuhnlein, Patterns and correlates of extrapair paternity in American redstarts (Setophaga ruticilla). *Behav. Ecol.* **8**, 612–621 (1997).

315. M. W. Reudink, P. P. Marra, T. K. Kyser, P. T. Boag, K. M. Langin, L. M. Ratcliffe, Non-breeding season events influence sexual selection in a long-distance migratory bird. *Proc. R. Soc. B Biol. Sci.* **276**, 1619–1626 (2009).

316. S. L. Balenger, L. Scott Johnson, H. L. Mays Jr, B. S. Masters, Extra-pair paternity in the socially monogamous mountain bluebird Sialia currucoides and its effect on the potential for sexual selection. *J. Avian Biol.* **40**, 173–180 (2009).

317. E. L. O’Brien, R. D. Dawson, Plumage color and food availability affect male reproductive success in a socially monogamous bird. *Behav. Ecol.* **22**, 66–72 (2011).

318. S. B. Meek, R. J. Robertson, P. T. Boag, Extrapair paternity and intraspecific brood parasitism in eastern bluebirds revealed by DNA fingerprinting. *Auk*. **111**, 739–744 (1994).

319. G. Segelbacher, D. Kabisch, M. Stauss, J. Tomiuk, Extra-pair young despite strong pair bonds in the European Nuthatch (Sitta europaea). *J. Ornithol.* **146**, 99–102 (2005).

320. M. K. Schwartz, Female-solicited extrapair matings in Humboldt penguins fail to produce extrapair fertilizations. *Behav. Ecol.* **10**, 242–250 (1999).

321. B. F. Sousa, D. F. Westneat, Positive association between social and extra-pair mating in a polygynous songbird, the dickcissel (Spiza americana). *Behav. Ecol. Sociobiol.* **67**, 243–255 (2013).

322. A. Celis-Murillo, W. Schelsky, T. J. Benson, M. I. M. Louder, M. P. Ward, Patterns, correlates, and paternity consequences of extraterritorial foray behavior in the field sparrow (Spizella pusilla): an automated telemetry approach. *Behav. Ecol. Sociobiol.* **71**, 45 (2017).

323. D. J. Delehanty, R. C. Fleischer, M. A. Colwell, L. W. Oring, Sex-role reversal and the absence of extra-pair fertilization in Wilson’s phalaropes. *Anim. Behav.* **55**, 995–1002 (1998).

324. J. Gonzalez-Solis, E. Sokolov, P. H. Becker, Courtship feedings, copulations and paternity in common terns, Sterna hirundo. *Anim. Behav.* **61**, 1125–1132 (2001).

325. M. Griggio, G. Matessi, G. Marin, No evidence of extra‐pair paternity in a colonial seabird, the common tern *(Sterna hirundo)*. *Ital. J. Zool.* **71**, 219–222 (2004).

326. V. Saladin, M. Ritschard, A. Roulin, P. Bize, H. Richner, Analysis of genetic parentage in the tawny owl (Strix aluco) reveals extra-pair paternity is low. *J. Ornithol.* **148**, 113–116 (2007).

327. P. J. Cordero, J. P. Veiga, J. Moreno, D. T. Parkin, Extra-pair paternity in the facultatively polygynous spotless starling, Sturnus unicolor. *Behav. Ecol. Sociobiol.* **54**, 1–6 (2003).

328. E. García-Vigón, P. J. Cordero, J. P. Veiga, Exogenous testosterone in female spotless starlings reduces their rate of extrapair offspring. *Anim. Behav.* **76**, 345–353 (2008).

329. A. Loyau, B. Moureau, M. Richard, P. Christe, P. Heeb, G. Sorci, Cross-amplification of polymorphic microsatellites reveals extra-pair paternity and brood parasitism in Sturnus vulgaris. *Mol. Ecol. Notes*. **5**, 135–139 (2005).

330. R. Pinxten, et al., Extra-pair paternity and intraspecific brood parasitism in the European starling, Sturnus vulgaris: evidence from DNA fingerprinting. *Anim. Behav.* **45**, 795–809 (1993).

331. H. G. Smith, M. I. Sandell, Intersexual competition in a polygynous mating system. *Oikos*. **83**, 484–495 (1998).

332. M. M. Baumgarten, A. B. Kohlrausch, C. Yumimiyaki, T. R. Ochotorena de Freitas, A. Mellender de Araujo, DNA Fingerprinting and Parentage in Masked (Sula Dactylatra) and Brown (S. Leucogaster) Boobies. *Ornitologia Neotropical*, 319–326 (2001).

333. D. J. Anderson, P. T. Boag, No Extra-pair Fertilization Observed in Nazca Booby (Sula granti) Broods. *Wilson J. Ornithol.* **118**, 244–247 (2006).

334. A. G. Ramos, S. O. Nunziata, S. L. Lance, C. Rodríguez, B. C. Faircloth, P. A. Gowaty, H. Drummond, Habitat structure and colony structure constrain extrapair paternity in a colonial bird. *Anim. Behav.* **95**, 121–127 (2014).

335. P. C. Baião, P. G. Parker, No Evidence of Extra-Pair Fertilization in Red-footed Boobies *(Sula sula)*. *Waterbirds*. **32**, 179–182 (2009).

336. O. R. Moore, B. J. M. Stutchbury, J. S. Quinn, Extrapair mating system of an asynchronously breeding tropical songbird: The Mangrove Swallow. *Auk*. **116**, 1039–1046 (1999).

337. C. A. Barber, R. J. Robertson, P. T. Boag, Experimental mate replacement does not increase extra-pair paternity in tree swallows. *Proc. R. Soc. B Biol. Sci.* **265**, 2187–2190 (1998).

338. C. A. Barber, R. J. Robertson, P. T. Boag, The high frequency of extra-pair paternity in tree swallows is not an artifact of nestboxes. *Behav. Ecol. Sociobiol.* **38**, 425–430 (1996).

339. K. F. Conrad, P. V. Johnston, C. Crossman, B. Kempenaers, R. J. Robertson, N. T. Wheelwright, T. Boag, High levels of extra-pair paternity in an isolated, low-density, island population of tree swallows (Tachycineta bicolor). *Mol. Ecol.* **10**, 1301–1308 (2001).

340. K. E. Delmore, O. Kleven, T. Laskemoen, S. A. Crowe, J. T. Lifjeld, R. J. Robertson, Sex allocation and parental quality in tree swallows. *Behav. Ecol.* **19**, 1243–1249 (2008).

341. P. O. Dunn, L. A. Whittingham, Search costs influence the spatial distribution, but not the level, of extra-pair mating in tree swallows. *Behav. Ecol. Sociobiol.* **61**, 449–454 (2006).

342. A. Lessard, A. Bourret, M. Bélisle, F. Pelletier, D. Garant, Individual and environmental determinants of reproductive success in male tree swallow (Tachycineta bicolor). *Behav. Ecol. Sociobiol.* **68**, 733–742 (2014).

343. P.-P. Bitton, E. L. O’Brien, R. D. Dawson, Plumage brightness and age predict extrapair fertilization success of male tree swallows, Tachycineta bicolor. *Anim. Behav.* **74**, 1777–1784 (2007).

344. M. K. Stapleton, O. Kleven, J. T. Lifjeld, R. J. Robertson, Female tree swallows (Tachycineta bicolor) increase offspring heterozygosity through extrapair mating. *Behav. Ecol. Sociobiol.* **61**, 1725–1733 (2007).

345. S. Van Wijk, A. Bourret, M. Bélisle, D. Garant, F. Pelletier, The influence of iridescent coloration directionality on male tree swallows’ reproductive success at different breeding densities. *Behav. Ecol. Sociobiol.* **70**, 1557–1569 (2016).

346. L. A. Whittingham, P. O. Dunn, Survival of extrapair and within-pair young in tree swallows. *Behav. Ecol.* **12**, 496–500 (2001).

347. L. A. Whittingham, P. O. Dunn, Experimental evidence that brighter males sire more extra-pair young in tree swallows. *Mol. Ecol.* **25**, 3706–3715 (2016).

348. V. Ferretti, V. Massoni, F. Bulit, D. W. Winkler, I. J. Lovette, Heterozygosity and fitness benefits of extrapair mate choice in White-rumped Swallows (Tachycineta leucorrhoa). *Behav. Ecol.* **22**, 1178–1186 (2011).

349. V. Ferretti, M. Liljesthröm, A. S. López, I. J. Lovette, D. W. Winkler, Extra-pair paternity in a population of Chilean swallows breeding at 54 degrees south. *J. Field Ornithol.* **87**, 155–161 (2016).

350. T. R. Birkhead, T. Burke, R. Zann, F. M. Hunter, A. P. Krupa, Extra-pair paternity and intraspecific brood parasitism in wild zebra finches Taeniopygia guttata, revealed by DNA fingerprinting. *Behav. Ecol. Sociobiol.* **27**, 316–324 (1990).

351. S. C. Griffith, C. E. Holleley, M. M. Mariette, S. R. Pryke, N. Svedin, Low level of extrapair parentage in wild zebra finches. *Anim. Behav.* **79**, 261–264 (2010).

352. C. L. Abbott, M. C. Double, R. Gales, A. Cockburn, Copulation behaviour and paternity in shy albatrosses (Thalassarche cauta). *J. Zool.* **270**, 628–635 (2006).

353. S. Lorentsen, T. Amundsen, K. Anthonisen, J. T. Lifjeld, Molecular evidence for extrapair paternity and female-female pairs in Antarctic petrels. *The Auk*. **117**, 1042–1047 (2000).

354. C. E. Tarwater, J. D. Brawn, J. D. Maddox, Low extrapair paternity observed in a tropical bird despite ample opportunities for extrapair mating. *The Auk*. **130**, 733–741 (2013).

355. T. M. Haggerty, E. S. Morton, R. C. Fleischer, Genetic Monogamy in Carolina Wrens (Thryothorus ludovicianus). *The Auk*. **118**, 215 (2001).

356. E. R. A. Cramer, M. L. Hall, S. R. de Kort, I. J. Lovette, S. L. Vehrencamp, Infrequent extra-pair paternity in the banded wren, a synchronously breeding tropical passerine. *The Condor*. **113**, 637–645 (2011).

357. S. Douglas, D. D. Heath, D. J. Mennill, Low levels of extra-pair paternity in a neotropical duetting songbird, the rufous-and-white wren (*Thryothorus rufalbus* ). *The Condor*. **114**, 393–400 (2012).

358. M. Stanback, D. S. Richardson, C. Boix-Hinzen, J. Mendelsohn, Genetic monogamy in Monteiro’s hornbill, Tockus monteiri. *Anim. Behav.* **63**, 787–793 (2002).

359. E. K. Bowers, A. M. Forsman, B. S. Masters, B. G. P. Johnson, L. S. Johnson, S. K. Sakaluk, C. F. Thompson, Increased extra-pair paternity in broods of aging males and enhanced recruitment of extra-pair young in a migratory bird: brief communication. *Evolution*. **69**, 2533–2541 (2015).

360. A. M. Z. Brylawski, L. A. Whittingham, An experimental study of mate guarding and paternity in house wrens. *Anim. Behav.* **68**, 1417–1424 (2004).

361. E. R. A. Cramer, T. Laskemoen, O. Kleven, K. LaBarbera, I. J. Lovette, J. T. Lifjeld, No evidence that sperm morphology predicts paternity success in wild house wrens. *Behav. Ecol. Sociobiol.* **67**, 1845–1853 (2013).

362. L. S. Johnson, B. G. Hicks, B. S. Masters, Increased cuckoldry as a cost of breeding late for male house wrens (Troglodytes aedon). *Behav. Ecol.* **13**, 670–675 (2002).

363. L. S. Johnson, J. L. Brubaker, B. G. P. Johnson, B. S. Masters, Evidence for a maternal effect benefiting extra-pair offspring in a songbird, the house wren *Troglodytes aedon*. *J. Avian Biol.* **40**, 248–253 (2009).

364. K. LaBarbera, P. E. Llambías, E. R. A. Cramer, T. D. Schaming, I. J. Lovette, Synchrony does not explain extrapair paternity rate variation in northern or southern house wrens. *Behav. Ecol.* **21**, 773–780 (2010).

365. N. E. Poirier, L. A. Whittingham, P. O. Dunn, Males achieve greater reproductive success through multiple broods, than through extrapair mating in house wrens. *Anim. Behav.* **67**, 1109–1116 (2004).

366. S. S. Soukup, C. F. Thompson, Social mating system affects the frequency of extra-pair paternity in house wrens. *Anim. Behav.* **54**, 1089–1105 (1997).

367. J. E. Brommer, P. Korsten, K. M. Bouwman, M. L. Berg, J. Komdeur, Is extrapair mating random? On the probability distribution of extrapair young in avian broods. *Behav. Ecol.* **18**, 895–904 (2007).

368. C. Biagolini-Jr, M. C. Costa, D. F. Perrella, P. V. Q. Zima, L. Ribeiro-Silva, M. R. Francisco, Extra-pair paternity in a Neotropical rainforest songbird, the White-necked Thrush Turdus albicollis (Aves: Turdidae). *Zool. Curitiba*. **33** (2016), doi:10.1590/S1984-4689zool-20160068.

369. B. J. M. Stutchbury, E. S. Morton, W. H. Piper, Extra-pair mating system of a synchronously breeding tropical songbird. *J. Avian Biol.* **29**, 72–78 (1998).

370. K. Rowe, P. Weatherhead, Social and ecological factors affecting paternity allocation in American robins with overlapping broods. *Behav. Ecol. Sociobiol.* **61**, 1283–1291 (2007).

371. D. V. Roeder, M. S. Husak, M. T. Murphy, Frequency of extra-pair paternity in scissor-tailed Flycatchers ( *Tyrannus forficatus* ) and other suboscines: are oscines and suboscines different? *Wilson J. Ornithol.* **128**, 494–502 (2016).

372. A. C. Dolan, M. T. Murphy, L. J. Redmond, K. Sexton, D. Duffield, Extrapair paternity and the opportunity for sexual selection in a socially monogamous passerine. *Behav. Ecol.* **18**, 985–993 (2007).

373. D. L. Rowe, M. T. Murphy, R. C. Fleischer, P. G. Wolf, High frequency of extra-pair paternity in eastern Kingbirds. *The Condor*. **103**, 845–851 (2001).

374. I. Henry, S. Antoniazza, S. Dubey, C. Simon, C. Waldvogel, R. Burri, A. Roulin, Multiple Paternity in Polyandrous Barn Owls (Tyto alba). *PLoS ONE*. **8**, e80112 (2013).

375. K. Berthier, F. Leippert, L. Fumagalli, R. Arlettaz, Massive nest-box supplementation boosts fecundity, survival and even immigration without altering mating and reproductive behaviour in a rapidly recovered bird population. *PLoS ONE*. **7**, e36028 (2012).

376. M. Martín-Vivaldi, J. G. Martínez, J. J. Palomino, M. Soler, Extrapair paternity in the Hoopoe Upupa epops: an exploration of the influence of interactions between breeding pairs, non-pair males and strophe length. *Ibis*. **144**, 236–247 (2002).

377. T. R. Birkhead, B. J. Hatchwell, R. Lindner, D. Blomqvist, E. J. Pellatt, R. Griffiths, J. T. Lifjeld, Extra-pair paternity in the common murre. *The Condor*. **103**, 158 (2001).

378. G. Ibarguchi, Male-biased Mutation Rates and the Overestimation of Extrapair Paternity: Problem, Solution, and Illustration Using Thick-Billed Murres (Uria lomvia, Alcidae). *J. Hered.* **95**, 209–210 (2004).

379. S. L. Harper, R. Vallender, R. J. Robertson, Male song variation and female mate choice in the golden-winged warbler. *The Condor*. **112**, 105–114 (2010).

380. A. J. Campomizzi, M. L. Morrison, J. A. DeWoody, S. L. Farrell, R. N. Wilkins, Win-stay, lose-switch and public information strategies for patch fidelity of songbirds with rare extra-pair paternity. *Sci. Rep.* **2** (2012), doi:10.1038/srep00294.

381. E. S. Morton, B. J. M. Stutchbury, J. S. Howlett, W. H. Piper, Genetic monogamy in blue-headed vireos and a comparison with a sympatric vireo with extrapair paternity. *Behav. Ecol.* **9**, 515–524 (1998).

382. C. B. V. Carvalho, R. H. Macedo, J. A. Graves, Breeding strategies of a socially monogamous neotropical passerine: extra-pair fertilizations, behavior, and morphology. *The Condor*. **108**, 579 (2006).

383. L. T. Manica, J. A. Graves, J. Podos, R. H. Macedo, Multimodal flight display of a neotropical songbird predicts social pairing but not extrapair mating success. *Behav. Ecol. Sociobiol.* **70**, 2039–2052 (2016).

384. B. J. M. Stutchbury, W. H. Piper, D. L. Neudorf, S. A. Tarof, J. M. Rhymer, G. Fuller, R. C. Fleischer, Correlates of extra-pair fertilization success in hooded warblers. *Behav. Ecol. Sociobiol.* **40**, 119–126 (1997).

385. M. L. Grunst, A. S. Grunst, R. A. Gonser, E. M. Tuttle, Breeding synchrony and extrapair paternity in a species with alternative reproductive strategies. *J. Avian Biol.* **48**, 1087–1094 (2017).

386. E. M. Tuttle, Alternative reproductive strategies in the white-throated sparrow: behavioral and genetic evidence. *Behav. Ecol.* **14**, 425–432 (2003).

387. C. Eikenaar, F. Bonier, P. R. Martin, I. T. Moore, High rates of extra-pair paternity in two equatorial populations of rufous-collared sparrow, *Zonotrichia capensis*. *J. Avian Biol.* **44**, 600–602 (2013).

388. F. Bonier, P. R. Martin, K. S. Sheldon, J. P. Jensen, S. L. Foltz, J. C. Wingfield, Sex-specific consequences of life in the city. *Behav. Ecol.* **18**, 121–129 (2007).

389. E. A. MacDougall-Shackleton, Nonlocal male mountain white-crowned sparrows have lower paternity and higher parasite loads than males singing local dialect. *Behav. Ecol.* **13**, 682–689 (2002).

390. A. Poesel, H. L. Gibbs, D. A. Nelson, Extrapair fertilizations and the potential for sexual selection in a socially monogamous songbird. *The Auk*. **128**, 770–776 (2011).

391. B. C. Robertson, Genetic monogamy in the absence of paternity guards: the Capricorn silvereye, Zosterops lateralis chlorocephalus, on Heron Island. *Behav. Ecol.* **12**, 666–673 (2001).
