# Supplementary material for: Extra‐pair paternity in birds
Source: Mol Ecol. 2019 Oct 31;28(22):4864–82. doi: 10.1111/mec.15259 (PMC6899757; doi:10.1111/mec.15259)
Supplement: Supplementary file 2 [file MEC-28-4864-s002.docx]

**Supplemental Information for:**

**Extra-pair paternity in birds**

Lyanne Brouwer^1,2,3*^ & Simon C. Griffith^4^

^1^Department of Animal Ecology & Physiology, Institute for Water and Wetland Research, Radboud University, Nijmegen, The Netherlands

^2^Department of Animal Ecology, Netherlands Institute of Ecology (NIOO-KNAW), Wageningen, The Netherlands

^3^Division of Ecology and Evolution, Research School of Biology, The Australian National University, Canberra ACT 2601, Australia

^4^Department of Biological Sciences, Macquarie University, North Ryde, NSW 2109, Australia

**Table S2**. Overview of the studies on birds with breeding systems other than socially monogamous, reporting 105 rates of the percentage of extra-pair paternity (EPP) for offspring and/or for the percentage of broods (EPbr) with at least one extra-pair offspring. N= number of offspring sampled, Nbr= number of broods sampled, Lat=latitude, Long=longitude. Breeding system categories are as follows: 1 monogamy or polygyny, 2 polyandry (multiple males, single female), 3 polygynandry (multiple males and females), 4 cooperative breeder, 5 no social bond, 6 lekking. The remarks column indicates studies which were conducted over a large geographic range, intra- or conspecific brood parasitism (IBP/CBP) was present, we report a subset of the data, we had difficulty in extracting numbers (and thus should be treated with care), or details about extra-group paternity (EGP).

| Scientific name | Common name | N | EPP | Nbr | EPbr | Lat | Long | Breeding system | Remarks | Ref |
| --- | --- | --- | --- | --- | --- | --- | --- | --- | --- | --- |
| *Acrocephalus melanopogon* | moustached warbler | 44 | 27.3 | 18 | 38.9 | 47.93 | 16.75 | 2 |  | (*1*) |
| *Acrocephalus paludicola* | aquatic warbler | 340 | 39.1 | 75 | 76.0 | 53.33 | 22.67 | 5 |  | (*2*) |
| *Acrocephalus paludicola* | aquatic warbler | 70 | 35.7 | 18 | 50.0 | 53.48 | 23.00 | 5 |  | (*1*) |
| *Acrocephalus sechellensis* | Seychelles warbler | 55 | 38.2 | 45 | 40.0 | -4.32 | 55.65 | 4 |  | (*3*) |
| *Acrocephalus vaughani* | Henderson reed-warbler | 28 | 7.1 | - | - | -24.38 | -128.32 | 1,2 |  | (*4*) |
| *Actitis macularius* | spotted sandpiper | 34 | 2.9 | 9 | 11.1 | 47.17 | -94.42 | 2 | Control dataset | (*5*) |
| *Aegithalos caudatus* | long-tailed tit | 372 | 1.9 | 48 | 12.5 | 53.37 | -1.57 | 4 |  | (*6*) |
| *Aegithalos concinnus* | black-throated tit | 383 | 5.7 | 64 | 17.2 | 31.95 | 114.25 | 4 |  | (*7*) |
| *Aegithalos glaucogularis* | silver-throated bush tit | 412 | 6.8 | 63 | 33.3 | 31.83 | 114.02 | 4 |  | (*7*) |
| *Alectura lathami* | Australian brush-turkey | 65 | 27.7 | 10 | - | -27.85 | 153.17 | 5 |  | (*8*) |
| *Ammodramus caudacutus* | saltmarsh sparrow | - | - | 60 | 95.0 | 41.00 | -72.00 | 5 |  | (*9*) |
| *Anthoscopus minutus* | Cape pendulin tit | 42 | 7.1 | 13 | 15.4 | -33.68 | 18.43 | 4 |  | (*10*) |
| *Aphelocoma coerulescens* | Florida scrub-jay | 139 | 0.0 | 60 | 0.0 | 27.17 | -81.35 | 4 |  | (*11*) |
| *Aphelocoma ultramarina* | Mexican jay | 139 | 39.6 | 52 | 61.5 | 31.92 | -109.25 | 4 |  | (*12*) |
| *Bubalornis niger* | red-billed buffalo weaver | 55 | 34.5 | 25 | 32.0 | NA | NA | 3,4 |  | (*13*) |
| *Buteo galapagoensis* | Galapagos hawk | 22 | 0.0 | 10 | 0.0 | -0.23 | -90.70 | 2 |  | (*14*) |
| *Calcarius pictus* | Smith´s longspur | 114 | 0.9 | 31 | 3.2 | 58.77 | -94.15 | 3 |  | (*15*) |
| *Calocitta formosa* | white-throated magpie-jay | 105 | 33.3 | 32 | 71.9 | 10.83 | -85.62 | 4 | Difficult to extract sample sizes | (*16*) |
| *Campylorhynchus griseus* | bicolored wren | 222 | 2.3 | 99 | - | 8.50 | -67.60 | 4 |  | (*17*) |
| *Campylorhynchus nuchalis* | stripe-backed wren | 69 | 1.4 | 34 | 2.9 | NA | NA | 4 |  | (*18*) |
| *Centrocercus urophasianus* | greater sage-grouse | - | - | 10 | 20.0 | 37.67 | -118.83 | 5 |  | (*19*) |
| *Centrocercus urophasianus* | greater sage-grouse | - | - | 191 | 7.9 | 49.40 | -110.70 | 5 | Also IBP | (*20*) |
| *Centropus grillii* | black coucal | 127 | 14.2 | 35 | 37.1 | -8.68 | 34.08 | 2 |  | (*21*) |
| *Chiroxiphia lanceolata* | lance-tailed manakin |  |  | 147 | 15.0 | 8.20 | -82.20 | 6 | Subset of data with two nestlings that were both assigned. | (*22*) |
| *Chrysococcyx basalis* | Horsfield’s bronze-cuckoo | 42 | 0.0 | NA | NA | -35.27 | 149.15 | 5 |  | (*23*) |
| *Chthonicola sagittatus* | speckled warbler | 103 | 10.7 | 48 | 12.5 | -35.27 | 149.15 | 2 |  | (*24*) |
| *Clamator glandarius* | great spotted cuckoo |  |  | 19 | 68.4 | 42.62 | -5.43 | 5 |  | (*25*) |
| *Clamator glandarius* | great spotted cuckoo |  |  | 9 | 88.9 | 37.30 | -3.18 | 5 |  | (*25*) |
| *Clamator glandarius* | great spotted cuckoo | 57 | - | 10 | 20.0 | 37.18 | -3.05 | 5 |  | (*26*) |
| *Colaptes auratus* | northern flicker | 41 | 2.4 | 7 | 14.3 | 52.87 | -123.03 | 2 |  | (*27*) |
| *Colaptes campestris* | Campo Flicker | 90 | 1.1 | 33 | 3.0 | -15.93 | -47.92 | 4 | No EGP | (*28*) |
| *Corcorax melanorhamphos* | white-winged chough | 68 | 0.0 | 28 | 0.0 | -35.27 | 149.15 | 4 | Difficult to extract sample sizes | (*29*) |
| *Corvus brachyrhynchos* | American crow | 202 | 10.4 | 60 | 21.7 | 42.40 | -76.50 | 4 | EGP: 35 offspring from 17 broods | (*30*) |
| *Corvus corone* | carrion crow |  |  | 19 | 15.8 | 42.00 | -5.00 | 1,4 | Difficult to extract sample sizes | (*31*) |
| *Crotophaga major* | greater ani | 357 | 13.7 | 53 | 71.7 | 9.20 | -80.00 | 3 | EGP: 11 offspring from 5 clutches | (*32*) |
| *Cuculus canorus* | common cuckoo |  |  | 21 | 14.3 | 36.00 | 138.00 | 5 | Difficult to extract sample sizes | (*33*) |
| *Cyanocorax morio* | brown jay | 113 | 16.8 | 37 | 21.6 | 10.20 | -84.70 | 4 |  | (*34*) |
| *Dacelo novaeguineae* | laughing kookaburra | 140 | 0.0 | 62 | 0.0 | -35.27 | 149.10 | 4 |  | (*35*) |
| *Eclectus roratus* | Eclectus parrot | 198 | 8.1 | 99 | 8.1 | -12.75 | 143.28 | 4 | Sibship analyses | (*36*) |
| *Erythropygia coryphaeus* | Karoo scrub-robin | 33 | 18.2 | 12 | 33.3 | -33.68 | 18.43 | 4 | No EGP | (*37*) |
| *Gallinula mortierii* | Tasmanian native hen | 28 | 0.0 | 6 | 0.0 | -42.68 | 148.02 | 2,3 |  | (*38*) |
| *Guira guira* | Guira cuckoo | 99 | 11.1 | - | - | -15.78 | -47.93 | 1,2,3 | Complicated because all sorts of social groups present. | (*39*) |
| *Gymnorhina tibicen* | Australian magpie | 79 | 44.3 | - | - | -37.72 | 144.40 | 4 |  | (*40*) |
| *Gymnorhina tibicen* | Australian magpie | 43 | 81.4 | 22 | 86.4 | -31.90 | 115.98 | 4 | Also IBP | (*41*) |
| *Irediparra gallinacea* | comb-crested jacana | 35 | 2.9 | 10 | 10.0 | -19.57 | 146.78 | 2 |  | (*42*) |
| *Jacana jacana* | wattled jacana | 235 | 1.3 | 74 | 2.7 | 9.23 | -79.50 | 2 |  | (*43*) |
| *Lamprotornis superbus* | superb starling |  |  | 208 | 17.8 | 0.12 | 37.87 | 4 |  | (*44*) |
| *Lanius collurio* | red-backed shrike | 19 | 5.3 | 6 | 16.7 | 46.20 | 9.40 | 4 |  | (*45*) |
| *Lophura leucomelanos* | kalij pheasant | 79 | 30.4 | 13 | 53.8 | 19.43 | -155.30 | 4 |  | (*46*) |
| *Malurus coronatus* | purple-crowned fairy-wren | 509 | 5.7 | 217 | 6.9 | -17.52 | 126.10 | 4 |  | (*47*) |
| *Malurus cyaneus* | superb fairy-wren | 27 | 66.7 | 12 | 83.3 | -35.62 | 138.03 | 4 |  | (*48*) |
| *Malurus cyaneus* | superb fairy-wren | 70 | 58.6 | 33 | 54.5 | -38.02 | 144.40 | 4 | Data for continuous habitat | (*49*) |
| *Malurus cyaneus* | superb fairy-wren | 44 | 41 | 16 | 75.0 | -36.78 | 145.80 | 4 | Data for linear habitat | (*49*) |
| *Malurus cyaneus* | superb fairy-wren | 2852 | 71.8 | - | - | -35.27 | 149.10 | 4 |  | (*50*) |
| *Malurus elegans* | red-winged fairy-wren | 47 | 68.1 | - | - | -34.35 | 116.02 | 4 | Data for linear habitat | (*51*) |
| *Malurus elegans* | red-winged fairy-wren | 932 | 56.4 | - | - | -34.35 | 116.02 | 4 | Data for continuous habitat | (*51*) |
| *Malurus melanocephalus* | red-backed fairy-wren | 673 | 53.8 | 231 | 68.4 | -17.37 | 125.75 | 4 |  | (*52*) |
| *Malurus splendens* | splendid fairy-wren | 386 | 42.2 | 159 | 55.3 | -34.33 | 139.03 | 4 |  | (*53*) |
| *Manorina melanocephala* | noisy miner | 85 | 5.9 | 35 | 5.7 | -27.33 | 152.55 | 4 |  | (*54*) |
| *Manorina melanophrys* | bell miner | 24 | 4.2 | 13 | 7.7 | -37.68 | 145.52 | 4 |  | (*55*) |
| *Melanerpes formicivorus* | acorn woodpecker | 386 | 0.0 | 123 | 0.0 | 36.37 | -121.05 | 4 | No EGP | (*56*) |
| *Meleagris gallopavo* | wild turkey | 250 | 11.6 | 31 | 45.2 | 36.37 | -121.05 | 2 | Some multiple maternity, and some quasi-parasitism | (*57*) |
| *Molothrus ater* | brown-headed cowbird | - | - | 42 | 42.9 | 50.18 | -98.38 | 5 |  | (*58*) |
| *Molothrus ater* | brown-headed cowbird | 43 | 4.7 | - | - | 50.18 | -98.38 | 5 |  | (59) |
| *Monias benschi* | subdesert mesite | 17 | 11.8 | - | - | -23.07 | 43.62 | 3,4 |  | (60) |
| *Myiopsitta monachus* | monk parakeet | - | - | 33 | 45.5 | -31.67 | -62.82 | 1,4 | Sibship analyses, also 3% IBP, possibly cooperative breeding | (61) |
| *Myiopsitta monachus* | monk parakeet | 34 | 0.0 | 12 | 0.0 | -31.00 | -59.00 | 4 |  | (62) |
| *Perisoreus infaustus* | Siberian jay | 40 | 0.0 | 23 | 0.0 | 62.37 | 21.50 | 4 | Subset of data from individuals sampled in nest | (63) |
| *Phalaropus fulicarius* | red phalarope | 70 | 8.6 | 18 | 33.3 | 69.40 | 81.82 | 2 |  | (64) |
| *Philetairus socius* | sociable weaver | 56 | 0.0 | 20 | 0.0 | -28.88 | 24.89 | 4 |  | (65) |
| *Philomachus pugnax* | ruff | - | - | 66 | 51.5 | 57.17 | 18.33 | 6 |  | (66) |
| *Philomachus pugnax* | ruff | - | - | 34 | 50.0 | 60.83 | 25.33 | 6 |  | (67) |
| *Picoides borealis* | red-cockaded woodpecker | 80 | 1.2 | 44 | 2.3 | 35.52 | -79.45 | 4 |  | (68) |
| *Plocepasser mahali* | white-browed sparrow weaver | 292 | 11.6 | 180 | 13.9 | -27.27 | 22.42 | 4 |  | (69) |
| *Pomatostomus temporalis* | grey-crowned babbler | 112 | 18.8 | 60 | 25.0 | -30.88 | 14.50 | 4 | Also IBP | (70) |
| *Porphyrio porphyrio* | pukeko | 73 | 0.0 | 12 | 0.0 | -45.93 | 170.25 | 4 |  | (71) |
| *Prunella collaris* | Alpine accentor | 110 | 0.0 | 38 | 0.0 | 42.97 | 1.58 | 2 |  | (72) |
| *Prunella collaris* | Alpine accentor |  |  | 5 | 60.0 | 46.20 | 7.63 | 2 |  | (73) |
| *Prunella modularis* | dunnock | 133 | 0.8 | 45 | 2.2 | 52.18 | 0.12 | 1,2,3 |  | (74) |
| *Prunella modularis* | dunnock | 288 | 17.0 | 98 | 26.5 | -45.87 | 170.50 | 1,2 |  | (75) |
| *Psaltriparus minimus* | bushtit | 59 | 0.0 | 10 | 0.0 | 31.85 | -109.25 | 4 |  | (76) |
| *Pseudopodoces humilis* | ground tit | 352 | 0.3 | 75 | 1.3 | 30.48 | 91.10 | 4 |  | (77) |
| *Pseudopodoces humilis* | ground tit | 966 | 12.6 | 172 | 32.0 | 34.23 | 102.03 | 1,4 |  | (78) |
| *Pyrrhura orcesi* | el oro parakeet | 104 | 1.0 | 28 | 3.6 | -3.65 | -79.77 | 4 | Difficult to extract sample sizes, also some paternity by within group helpers | (79) |
| *Ramphocinclus brachyurus* | white-breasted thrasher | 67 | 7.5 | 30 | 13.3 | 13.92 | -60.92 | 4 |  | (80) |
| *Remiz pendulinus* | penduline tit | 201 | 7.0 | 52 | 17.3 | 47.93 | 17.72 | 3 |  | (81) |
| *Remiz pendulinus* | penduline tit | 166 | 23.5 | 55 | 52.7 | 46.32 | 20.10 | 3 |  | (82) |
| *Sericornis frontalis* | white-browed scrubwren | 137 | 12.4 | 51 | 23.5 | -35.27 | 149.10 | 4 |  | (83) |
| *Sialia mexicana* | western bluebird | 324 | 35.2 | 72 | 70.8 | 35.88 | -106.03 | 4 |  | (84) |
| *Sialia mexicana* | western bluebird | 207 | 18.8 | 51 | 45.1 | 36.37 | -121.05 | 4 |  | (85) |
| *Sialia mexicana* | western bluebird | 1046 | 22.1 | 256 | 44.9 | 36.37 | -121.05 | 4 |  | (86) |
| *Sitta pusilla* | brown-headed nuthatch | 237 | 27.8 | 59 | 40.7 | 30.65 | -84.20 | 4 |  | (87) |
| *Stipiturus malachurus* | southern emu-wren | 50 | 12.0 | 27 | 14.8 | -38.38 | 141.60 | 4 |  | (88) |
| *Struthidea cinerea* | apostlebird |  |  | 78 | 2.6 | -31.00 | 142.00 | 2,4 | Subset data from monogamous pairs | (89) |
| *Struthidea cinerea* | apostlebird | 41 | 0.0 | 15 | 0.0 | -34.57 | 145.75 | 2,4 | EGP most likely 0 | (90) |
| *Struthio camelus* | ostrich | 61 | 49.2 | 6 | 100.0 | -1.40 | 36.80 | 2 | Also IBP | (91) |
| *Tetrao tetrix* | black grouse | 66 | 0.0 | 11 | 0.0 | 62.17 | 25.08 | 5 |  | (92) |
| *Tetrao tetrix* | black grouse |  |  | 130 | 3.8 | 63.00 | 26.00 | 5 |  | (93) |
| *Thryothorus leucotis* | buff-breasted wrens | 53 | 3.8 | 31 | 3.2 | 10.17 | -79.70 | 4 |  | (94) |
| *Tinamus major* | great tinamou | 121 | 24.0 | 28 | 57.1 | 10.43 | -83.98 | 2 | Also IBP | (95) |
| *Tryngites subruficollis* | buff-breasted sandpiper |  |  | 47 | 40.4 | 70.20 | -148.25 | 5 |  | (96) |
| *Turdoides bicolor* | pied babbler | 145 | 4.8 | - | - | -26.97 | 21.82 | 4 |  | (97) |
| *Turdoides squamiceps* | Arabian babbler | 186 | 0.0 | 44 | 0.0 | 30.77 | 35.27 | 4 |  | (98) |
| *Tympanuchus cupido* | prairie chicken |  |  | 25 | 44.0 | 44.33 | -89.63 | 6 |  | (99) |
| *Vanellus chilensis* | Southern lapwing | 41 | 9.8 | 16 | 18.8 | -15.77 | -47.87 | 4 | Data from groups where dominant male sampled, unclear whether helpers present or not | (100) |

**References**

1. K. Schulze-Hagen, I. Swatschek, A. Dyrcz, M. Wink, Multiple paternity in broods of aquatic warblers Acrocephalus paludicola: first results of a DNA-fingerprinting study. *J. Ornithol.* **134**, 145–154 (1993).

2. B. Leisler, M. Wink, Frequencies of multiple paternity in three *Acrocephalus* species (Aves Sylviidae) with different mating systems (*A. palustris, A. arundinaceus, A. paludicola* ). *Ethol. Ecol. Evol.* **12**, 237–249 (2000).

3. D. S. Richardson, F. L. Jury, K. Blaakmeer, J. Komdeur, T. Burke, Parentage assignment and extra-group paternity in a cooperative breeder: the Seychelles warbler (*Acrocephalus sechellensis*). *Mol. Ecol.* **10**, 2263–2273 (2001).

4. M. de L. Brooke, I. R. Hartley, Nesting Henderson reed-warblers (Acrocephalus vaughani taiti) studied by DNA fingerprinting: unrelated coalitions in a stable habitat? *The Auk*. **112**, 77–86 (1995).

5. L. W. Oring, R. C. Fleischer, J. M. Reed, K. E. Marsden, Cuckoldry through stored sperm in the sequentially polyandrous spotted sandpiper. *Nature*. **359**, 631–633 (1992).

6. B. J. Hatchwell, D. J. Ross, N. Chaline, M. K. Fowlie, T. Burke, Parentage in the cooperative breeding system of long-tailed tits, Aegithalos caudatus. *Anim. Behav.* **64**, 55–63 (2002).

7. J. Li, Y. Liu, Y. Wang, Z. Zhang, Extra-pair paternity in two sympatric Aegithalos tits: patterns and implications. *J. Ornithol.* **155**, 83–90 (2014).

8. S. M. Birks, Paternity in the Australian brush-turkey, *Alectura lathami* , a megapode bird with uniparental male care. *Behav. Ecol.* **8**, 560–568 (1997).

9. C. E. Hill, C. Gjerdrum, C. S. Elphick, Extreme Levels of Multiple Mating Characterize the Mating System of the Saltmarsh Sparrow (*Ammodramus caudacutus* ). *The Auk*. **127**, 300–307 (2010).

10. A. D. Ball *et al.*, Levels of extra-pair paternity are associated with parental care in penduline tits (Remizidae). *Ibis*. **159**, 449–455 (2017).

11. J. S. Quinn, G. E. Woolfenden, J. W. Fitzpatrick, B. N. White, Multi-Locus DNA Fingerprinting Supports Genetic Monogamy in Florida Scrub-Jays. *Behav. Ecol. Sociobiol.* **45**, 1–10 (1999).

12. S.-H. Li, J. L. Brown, High frequency of extrapair fertilization in a plural breeding bird, the Mexican jay, revealed by DNA microsatellites. *Anim. Behav.* **60**, 867–877 (2000).

13. M. Winterbottom, T. Burke, T. R. Birkhead, The phalloid organ, orgasm and sperm competition in a polygynandrous bird: the red-billed buffalo weaver (Bubalornis niger). *Behav. Ecol. Sociobiol.* **50**, 474–482 (2001).

14. J. Faaborg *et al.*, Confirmation of cooperative polyandry in the Galapagos hawk (Buteo galapagoensis). *Behav. Ecol. Sociobiol.* **36**, 83–90 (1995).

15. J. V. Briskie, R. Montgomerie, T. Põldmaa, P. T. Boag, Paternity and paternal care in the polygynandrous Smith’s longspur. *Behav. Ecol. Sociobiol.* **43**, 181–190 (1998).

16. E. C. Berg, Parentage and reproductive success in the white-throated magpie-jay, Calocitta formosa, a cooperative breeder with female helpers. *Anim. Behav.* **70**, 375–385 (2005).

17. J. Haydock, P. G. Parker, K. N. Rabenold, Extra-pair paternity uncommon in the cooperatively breeding bicolored wren. *Behav. Ecol. Sociobiol.* **38**, 1–16 (1996).

18. P. P. Rabenold, K. N. Rabenold, W. H. Piper, J. Haydock, S. W. Zack, Shared paternity revealed by genetic analysis in cooperatively breeding tropical wrens. *Nature*. **348**, 538–540 (1990).

19. K. Semple, R. K. Wayne, R. M. Gibson, Microsatellite analysis of female mating behaviour in lek-breeding sage grouse. *Mol. Ecol.* **10**, 2043–2048 (2001).

20. K. L. Bird *et al.*, The secret sex lives of sage-grouse: multiple paternity and intraspecific nest parasitism revealed through genetic analysis. *Behav. Ecol.* **24**, 29–38 (2013).

21. C. Muck, B. Kempenaers, S. Kuhn, M. Valcu, W. Goymann, Paternity in the classical polyandrous black coucal (*Centropus grillii*)—a cuckoo accepting cuckoldry? *Behav. Ecol.* **20**, 1185–1193 (2009).

22. E. H. DuVal, B. Kempenaers, Sexual selection in a lekking bird: the relative opportunity for selection by female choice and male competition. *Proc. R. Soc. B Biol. Sci.* **275**, 1995–2003 (2008).

23. N. E. Langmore, G. J. Adcock, R. M. Kilner, The spatial organization and mating system of Horsfield’s bronze-cuckoos, Chalcites basalis. *Anim. Behav.* **74**, 403–412 (2007).

24. J. L. Gardner, R. D. Magrath, P. D. Olsen, Speckled warblers break cooperative rules: absence of helping in a group-living member of the Pardalotidae. *Anim. Behav.* **67**, 719–728 (2004).

25. D. Bolopo *et al.*, Flexible mating patterns in an obligate brood parasite. *Ibis*. **159**, 103–112 (2017).

26. J. G. Martinez *et al.*, Microsatellite typing reveals mating patterns in the brood parasitic great spotted cuckoo (*Clamator glandarius*). *Mol. Ecol.* **7**, 289–297 (1998).

27. K. L. Wiebe, B. Kempenaers, The social and genetic mating system in flickers linked to partially reversed sex roles. *Behav. Ecol.* **20**, 453–458 (2009).

28. R. I. Dias, R. H. Macedo, D. Goedert, M. S. Webster, Cooperative Breeding in the Campo Flicker II: Patterns of Reproduction and Kinship. *The Condor*. **115**, 855–862 (2013).

29. R. Heinsohn, P. Dunn, S. Legge, M. Double, Coalitions of Relatives and Reproductive Skew in Cooperatively Breeding White-Winged Choughs. *Proc. Biol. Sci.* **267**, 243–249 (2000).

30. A. K. Townsend, Extrapair Copulations Predict Extrapair Fertilizations in the American Crow. *Condor Ornithol. Appl.* **111**, 387–392 (2009).

31. V. Baglione, J. M. Marcos, D. Canestrari, J. Ekman, Direct fitness benefits of group living in a complex cooperative society of carrion crows, Corvus corone corone. *Anim. Behav.* **64**, 887–893 (2002).

32. C. Riehl, Mating system and reproductive skew in a communally breeding cuckoo: hard-working males do not sire more young. *Anim. Behav.* **84**, 707–714 (2012).

33. K. Marchetti, H. Nakamura, H. L. Gibbs, Host-Race Formation in the Common Cuckoo. *Science*. **282**, 471–472 (1998).

34. D. Williams, Female control of reproductive skew in cooperatively breeding brown jays (*Cyanocorax morio*). *Behav. Ecol. Sociobiol.* **55**, 370–380 (2004).

35. S. Legge, A. Cockburn, Social and mating system of cooperatively breeding laughing kookaburras (*Dacelo novaeguineae*). *Behav. Ecol. Sociobiol.* **47**, 220–229 (2000).

36. R. Heinsohn, D. Ebert, S. Legge, R. Peakall, Genetic evidence for cooperative polyandry in reverse dichromatic Eclectus parrots. *Anim. Behav.* **74**, 1047–1054 (2007).

37. Â. M. Ribeiro, P. Lloyd, K. A. Feldheim, R. C. K. Bowie, Microgeographic socio-genetic structure of an African cooperative breeding passerine revealed: integrating behavioural and genetic data. *Mol. Ecol.* **21**, 662–672 (2012).

38. H. L. Gibbs, A. W. Goldizen, Cindy Bullough, A. R. Goldizen, Parentage analysis of multi-male social groups of Tasmanian native hens (Tribonyx mortierii): genetic Evidence for monogamy and polyandry. *Behav. Ecol. Sociobiol.* **35**, 363–371 (1994).

39. M. R. Lima, R. H. Macedo, L. Muniz, A. Pacheco, J. A. Graves, Group composition, mating system, and relatedness in the communally breeding Guira cuckoo (*Guira guira*) in Central Brazil. *The Auk*. **128**, 475–486 (2011).

40. K. L. Durrant, J. M. Hughes, Differing rates of extra-group paternity between two populations of the Australian magpie (*Gymnorhina tibicen*). *Behav. Ecol. Sociobiol.* **57**, 536–545 (2005).

41. J. M. Hughes *et al.*, High levels of extra-group paternity in a population of Australian magpies Gymnorhina tibicen: evidence from microsatellite analysis. *Mol. Ecol.* **12**, 3441–3450 (2003).

42. S. M. Haig, Parentage and relatedness in polyandrous comb-crested jacanas using ISSRs. *J. Hered.* **94**, 302–309 (2003).

43. S. T. Emlen, P. H. Wrege, M. S. Webster, Cuckoldry as a cost of polyandry in the sex–role–reversed wattled jacana, Jacana jacana. *Proc. R. Soc. Lond. B Biol. Sci.* **265**, 2359–2364 (1998).

44. L. R. Weinman, J. W. Solomon, D. R. Rubenstein, A comparison of single nucleotide polymorphism and microsatellite markers for analysis of parentage and kinship in a cooperatively breeding bird. *Mol. Ecol. Resour.* **15**, 502–511 (2015).

45. L. Fornasari, et al., testosterone in the breeding cycle of the male Red-backed Shrike lanius collurio. *Ethol.Ecol.Evol.* **4**, 193–196 (1992).

46. L. Zeng, J. T. Rotenberry, M. Zuk, T. K. Pratt, Z. Zhang, Social behavior and cooperative breeding in a precocial species: The Kalij Pheasant ( *Lophura leucomelanos* ) in Hawaii. *The Auk*. **133**, 747–760 (2016).

47. S. A. Kingma, M. L. Hall, A. Peters, Breeding synchronization facilitates extrapair mating for inbreeding avoidance. *Behav. Ecol.* **24**, 1390–1397 (2013).

48. D. Colombelli-Négrel, B. E. Schlotfeldt, S. Kleindorfer, High levels of extra-pair paternity in Superb Fairy-wrens in South Australia despite low frequency of auxiliary males. *Emu*. **109**, 300–304 (2009).

49. G. C. Bain, M. L. Hall, R. A. Mulder, Territory configuration moderates the frequency of extra-group mating in superb fairy-wrens. *Mol. Ecol.* **23**, 5619–5627 (2014).

50. A. Cockburn, M. Double, Cooperatively breeding superb fairy-wrens show no facultative manipulation of offspring sex ratio despite plausible benefits. *Behav Ecol Sociobiol*. **62**, 681–688 (2008).

51. L. Brouwer, M. van de Pol, A. Cockburn, Habitat geometry does not affect levels of extrapair paternity in an extremely unfaithful fairy-wren. *Behav. Ecol.* **25**, 531–537 (2014).

52. C. W. Varian-Ramos, W.R. Lindsay, J. Karubian, M. S. Webster, Female Red-backed Fairy-Wrens (*Malurus melanocephalus* ) do not appear to pay a cost for high rates of promiscuity. *The Auk*. **129**, 529–536 (2012).

53. M. S. Webster, K. A. Tarvin, E. M. Tuttle, S. Pruett-Jones, Reproductive promiscuity in the splendid fairy-wren: effects of group size and auxiliary reproduction. *Behav. Ecol.* **15**, 907–915 (2004).

54. T. Põldmaa, R. Montgomerie, P. Boag, Mating system of the cooperatively breeding noisy miner Manorina melanocephala, as revealed by DNA profiling. *Behav. Ecol. Sociobiol.* **37**, 137–143 (1995).

55. K. F. Conrad, R. J. Robertson, P. T. Boag, Frequency of extrapair young increases in second broods of eastern phoebes. *Auk*. **115**, 497–502 (1998).

56. J. Haydock, W. D. Koenig, M. T. Stanback, Shared parentage and incest avoidance in the cooperatively breeding acorn woodpecker. *Mol. Ecol.* **10**, 1515–1525 (2001).

57. A. H. Krakauer, Sexual selection and the genetic mating system of wild turkeys. *The Condor*. **110**, 1–12 (2008).

58. B. Woolfenden, L. Gibbs, S. Sealy, High opportunity for sexual selection in both sexes of an obligate brood parasitic bird, the brown-headed cowbird (*Molothrus ater*). *Behav. Ecol. Sociobiol.* **52**, 417–425 (2002).

59. Alderson, G. W., Gibbs, H. L., & Sealy, S. G. Determining the reproductive behaviour of individual brown-headed cowbirds using microsatellite DNA markers. *Animal Behaviour*, *58*(4), 895–905 (1999).

60. N. Seddon *et al.*, Mating system, philopatry and patterns of kinship in the cooperatively breeding subdesert mesite Monias benschi. *Mol. Ecol.* **14**, 3573–3583 (2005).

61. J. J. Martínez, M. C. de Aranzamendi, J. F. Masello, E. H. Bucher, Genetic evidence of extra-pair paternity and intraspecific brood parasitism in the monk parakeet. *Front. Zool.* **10**, 68 (2013).

62. A. G. Da SILVA, J. R. Eberhard, T. F. Wright, M. L. Avery, M. A. Russello, Genetic evidence for high propagule pressure and long-distance dispersal in monk parakeet (*Myiopsitta monachus*) invasive populations. *Mol. Ecol.* **19**, 3336–3350 (2010).

63. P. Gienapp, J. Merilä, High Fidelity – No Evidence for Extra-Pair Paternity in Siberian Jays (Perisoreus infaustus). *PLoS ONE*. **5**, e12006 (2010).

64. J. Dale, R. Montgomerie, D. Michaud, P. Boag, Frequency and timing of extrapair fertilisation in the polyandrous red phalarope (*Phalaropus fulicarius* ). *Behav. Ecol. Sociobiol.* **46**, 50–56 (1999).

65. R. Covas, A. Dalecky, A. Caizergues, C. Doutrelant, Kin associations and direct vs indirect fitness benefits in colonial cooperatively breeding sociable weavers Philetairus socius. *Behav. Ecol. Sociobiol.* **60**, 323–331 (2006).

66. K. A. Thuman, S. C. Griffith, Genetic similarity and the nonrandom distribution of paternity in a genetically highly polyandrous shorebird. *Anim. Behav.* **69**, 765–770 (2005).

67. D. B. Lank, High frequency of polyandry in a lek mating system. *Behav. Ecol.* **13**, 209–215 (2002).

68. S. M. Haig, J. R. Walters, J. H. Plissner, Genetic evidence for monogamy in the cooperatively breeding red-cockaded woodpecker. *Behav. Ecol. Sociobiol.* **34**, 295–303 (1994).

69. X. A. Harrison, J. E. York, D. L. Cram, M. C. Hares, A. J. Young, Complete reproductive skew within white-browed sparrow weaver groups despite outbreeding opportunities for subordinates of both sexes. *Behav. Ecol. Sociobiol.* **67**, 1915–1929 (2013).

70. C. J. Blackmore, R. Heinsohn, Variable mating strategies and incest avoidance in cooperatively breeding grey-crowned babblers. *Anim. Behav.* **75**, 63–70 (2008).

71. I. G. Jamieson, J. S. Quinn, P. A. Rose, B. N. White, Shared paternity among non-relatives is a result of an egalitarian mating system in a communally breeding bird, the pukeko. *Proc. R. Soc. Lond. B Biol. Sci.* **257**, 271–277 (1994).

72. I. R. Hartley *et al.*, The polygynandrous mating system of the alpine accentor, Prunella collaris. II. Multiple paternity and parental effort. *Anim. Behav.* **49**, 789–803 (1995).

73. L. Heer, Cooperative breeding by Alpine AccentorsPrunella collaris: Polygynandry, territoriality and multiple paternity. *J. Für Ornithol.* **137**, 35–51 (1996).

74. T. Burke, N. B. Davies, M. W. Bruford, B. J. Hatchwell, Parental care and mating behaviour of polyandrous dunnocks *Prunella modularis* related to paternity by DNA fingerprinting. *Nature*. **338**, 249–251 (1989).

75. E. S. A. Santos, L. L. S. Santos, M. Lagisz, S. Nakagawa, Conflict and cooperation over sex: the consequences of social and genetic polyandry for reproductive success in dunnocks. *J. Anim. Ecol.* **84**, 1509–1519 (2015).

76. J. P. Bruce, J. S. Quinn, S. A. Sloane, B. N. White, DNA Fingerprinting Reveals Monogamy in the Bushtit, a Cooperatively Breeding Species. *The Auk*. **113**, 511–516 (1996).

77. L. E. Johannessen, D. Ke, X. Lu, J. T. Lifjeld, Geographical variation in patterns of parentage and relatedness in the co-operatively breeding Ground Tit Parus humilis: Parentage and relatedness in the Ground Tit. *Ibis*. **153**, 373–383 (2011).

78. C. Wang, X. Lu, Extra-pair paternity in relation to breeding synchrony in ground tits: an individual-based approach. *J. Avian Biol.* **45**, 561–565 (2014).

79. N. Klauke, G. Segelbacher, H. M. Schaefer, Reproductive success depends on the quality of helpers in the endangered, cooperative El Oro parakeet ( *Pyrrhura orcesi* ). *Mol. Ecol.* **22**, 2011–2027 (2013).

80. H. J. Temple, J. I. Hoffman, W. Amos, Group structure, mating system and extra-group paternity in the co-operatively breeding White-breasted Thrasher *Ramphocinclus brachyurus*. *Ibis*. **151**, 99–112 (2009).

81. B. Schleicher, H. Hoi, F. Valera, M. Hoi-Leitner, The Importance of Different Paternity Guards in the Polygynandrous Penduline Tit (*Remiz pendulinus*). *Behaviour*. **134**, 941–959 (1997).

82. R. E. van Dijk *et al.*, Nest desertion is not predicted by cuckoldry in the Eurasian penduline tit. *Behav. Ecol. Sociobiol.* **64**, 1425–1435 (2010).

83. L. A. Whittingham, P. O. Dunn, R. D. Magrath, Relatedness, polyandry and extra-group paternity in the cooperatively-breeding white-browed scrubwren (*Sericornis frontalis*). *Behav. Ecol. Sociobiol.* **40**, 261–270 (1997).

84. A. C. Jacobs, J. M. Fair, M. Zuk, Coloration, Paternity, and Assortative Mating in Western Bluebirds. *Ethology*. **121**, 176–186 (2015).

85. J. L. Dickinson, J. J. Akre, Extrapair paternity, inclusive fitness, and within‐group benefits of helping in western bluebirds. *Mol. Ecol.* **7**, 95–105 (1998).

86. E. D. Ferree, J. L. Dickinson, Natural extrapair paternity matches receptivity patterns in unguarded females: evidence for importance of female choice. *Anim. Behav.* **82**, 1167–1173 (2011).

87. K.-L. Han, J. A. Cox, R. T. Kimball, Uncommon Levels of Relatedness and Parentage in a Cooperatively Breeding Bird, the Brown-Headed Nuthatch ( *Sitta pusilla* ). *Wilson J. Ornithol.* **127**, 593–600 (2015).

88. G. S. Maguire, R. A. Mulder, Low levels of extra-pair paternity in southern emu-wrens (Aves : Maluridae). *Aust. J. Zool.* **56**, 79–84 (2008).

89. M. H. Warrington, L. A. Rollins, N. J. Raihani, A. F. Russell, S. C. Griffith, Genetic monogamy despite variable ecological conditions and social environment in the cooperatively breeding apostlebird. *Ecol. Evol.* **3**, 4669–4682 (2013).

90. I. A. Woxvold, R. A. Mulder, Mixed mating strategies in cooperatively breeding apostlebirds Struthidea cinerea. *J. Avian Biol.* **0**, 071202183307005–0 (2007).

91. C. N. Kimwele, J. A. Graves, A molecular genetic analysis of the communal nesting of the ostrich (Struthio camelus). *Mol. Ecol.* **12**, 229–236 (2002).

92. R. V. Alatalo, J. Hoglund, A. Lundberg, P. T. Rintam„ki, B. Silverin, Testosterone and male mating success on the black grouse leks. *Proc. R. Soc. Lond. Ser. B-Biol. Sci.* **263**, 1697–1702 (1996).

93. C. Lebigre, R. V. Alatalo, H. Siitari, S. Parri, Restrictive mating by females on black grouse leks. *Mol. Ecol.* **16**, 4380–4389 (2007).

94. S. A. Gill, M. J. Vonhof, B. J. M. Stutchbury, E. S. Morton, J. S. Quinn, No evidence for acoustic mate-guarding in duetting buff-breasted wrens (*Thryothorus leucotis*). *Behav. Ecol. Sociobiol.* **57**, 557–565 (2005).

95. P. L. R. Brennan, Mixed paternity despite high male parental care in great tinamous and other Palaeognathes. *Anim. Behav.* **84**, 693–699 (2012).

96. R. B. Lanctot, K. T. Scribner, R. B. Lanctot, P. J. Weatherhead, B. Kempenaers, Lekking Without a Paradox in the Buff‐Breasted Sandpiper. *Am. Nat.* **149**, 1051–1070 (1997).

97. M. J. Nelson-Flower *et al.*, Monogamous dominant pairs monopolize reproduction in the cooperatively breeding pied babbler. *Behav. Ecol.* **22**, 559–565 (2011).

98. K. J. Lundy, P. G. Parker, A. Zahavi, Reproduction by subordinates in cooperatively breeding Arabian babblers is uncommon but predictable. *Behav. Ecol. Sociobiol.* **43**, 173–180 (1998).

99. B. D. Hess, P. O. Dunn, L. Whittingham, Females Choose Multiple Mates in the Lekking Greater Prairie-Chicken (*Tympanuchus cupido*). *The Auk*. **129**, 133–139 (2012).

100. V. Saracura, R. H. Macedo, D. Blomqvist, Genetic parentage and variable social structure in breeding southern lapwings. *The Condor*. **110**, 554–558 (2008).
