# Supplementary material for: Extra‐pair paternity in birds
Source: Mol Ecol. 2019 Oct 31;28(22):4864–82. doi: 10.1111/mec.15259 (PMC6899757; doi:10.1111/mec.15259)
Supplement: Supplementary file 3 [file MEC-28-4864-s003.docx]

**Supplemental Information for:**

**Extra-pair paternity in birds**

Lyanne Brouwer^1,2,3*^ & Simon C. Griffith^4^

^1^Department of Animal Ecology & Physiology, Institute for Water and Wetland Research, Radboud University, Nijmegen, The Netherlands

^2^Department of Animal Ecology, Netherlands Institute of Ecology (NIOO-KNAW), Wageningen, The Netherlands

^3^Division of Ecology and Evolution, Research School of Biology, The Australian National University, Canberra ACT 2601, Australia

^4^Department of Biological Sciences, Macquarie University, North Ryde, NSW 2109, Australia

**Table S3**. Overview of the excluded studies reporting rates of the percentage of extra-pair paternity (EPP) for offspring and/or for the percentage of broods (EPbr) with at least one extra-pair offspring, with the reason for exclusion. N= number of offspring sampled, Nbr= number of broods sampled, Lat=latitude, Long=longitude. Note that the studies excluded based on ‘identical dataset' may not be exhaustive.

| Scientific name | Common name | N | EPP | Nbr | EPbr | Lat | Long | Habitat | Reason for exclusion | Ref |
| --- | --- | --- | --- | --- | --- | --- | --- | --- | --- | --- |
| *Ficedula hypoleuca* | pied flycatcher | 225 | 4.4 | 43 | 3 | 62.62 | 26.33 | 1,8,9 | Experimental | (*1*) |
| *Acrocephalus schoenobaenus* | sedge warbler | 143 | 8.4 | 33 | 27.3 | 51.45 | -0.52 | 12 | Identical dataset | (*2*) |
| *Agelaius phoeniceus* | red-winged blackbird | 1479 | 40.1 | 537 | 54.9 | 37.24 | -87.05 | 12,9 | Experimental | (*3*) |
| *Aphelocoma ultramarina* | Mexican jay | 93 | 16.1 | 31 | 38.7 | 31.88 | -109.20 | 1,4 | Identical dataset | (*4*) |
| *Carpodacus erythrinus* | scarlet rosefinch | 451 | 17.7 | 104 | 32.7 | 48.82 | 13.93 | 1,8,6,11,9 | Identical dataset | (*5*) |
| *Carpodacus erythrinus* | scarlet rosefinch | 453 | - | 108 | 38.0 | 48.82 | 13.93 | 1,8,6,11,9 | Identical dataset | (*6*) |
| *Carpodacus erythrinus* | scarlet rosefinch | - | - | 75 | 32.0 | 48.82 | 13.93 | 1,8,6,11,9 | Identical dataset | (*7*) |
| *Carpodacus erythrinus* | scarlet rosefinch | 266 | 18.0 | 62 | 33.9 | 48.82 | 13.93 | 1,8,6,11,9 | Identical dataset | (*8*) |
| *Carpodacus mexicanus* | house finch | 196 | 4.6 | 57 | 12.3 | 46.85 | -114.02 | 1,6,9 | Identical dataset | (*9*) |
| *Charadrius alexandrinus* | Kentish plover | 170 | 2.9 | 65 | 4.6 | 36.72 | 35.05 | - | Identical dataset | (*10*) |
| *Dendroica caerulescens* | black-throated blue warbler | 900 | 43.9 | 271 | 56.1 | 43.93 | -71.75 | 8 | Experimental | (*11*, *12*) |
| *Dendroica petechia* | yellow warbler | 355 | 36.6 | 90 | 58.9 | 44.57 | -76.33 | 1,6,9 | Identical dataset | (*13*) |
| *Dromaius novaehollandiae* | emu | 106 | 50.9 | 18 | 88.9 | -32.22 | 115.82 | - | semi-wild, enclosure, supplemental food and water | (*14*) |
| *Emberiza schoeniclus* | reed bunting | 529 | 38.9 | 132 | 63.6 | 46.90 | 6.93 | 12 | Identical dataset | (*15*) |
| *Falco naumanni* | lesser kestrel | 87 | 3.4 | 26 | 3.8 | 41.35 | -0.18 | - | Identical dataset | (*16*) |
| *Ficedula hypoleuca* | pied flycatcher | 533 | 19.9 | 113 | 39.8 | 41.07 | -3.45 | 1,8,9 | Identical dataset | (*17*) |
| *Forpus passerinus* | green-rumped parrotlet | 827 | 7.7 | 160 | 14.4 | 8.57 | -67.58 | - | Non-peer reviewed | (*18*) |
| *Geospiza fortis* | medium ground-finch | 223 | 19.7 | 93 | 35.5 | -0.42 | -90.37 | 6,8 | Identical dataset | (*19*) |
| *Geospiza scandens* | cactus finch | 159 | 7.5 | 66 | 15.2 | -0.42 | -90.37 | 15 | Identical dataset | (*20*) |
| *Geothlypis trichas* | yellowthroat | 139 | 22.3 | 41 | 48.8 | 43.38 | -88.02 | 7,12 | Identical dataset | (*21*) |
| *Geothlypis trichas* | yellowthroat | 234 | 18.8 | 63 | 44.4 | 43.38 | -88.02 | 7,12 | Identical dataset | (*22*) |
| *Hirundo rustica* | barn swallow | 44 | 29.5 | 9 | 55.6 | 57.20 | 10.00 | 3,4,9 | Identical dataset | (*23*) |
| *Hirundo rustica* | barn swallow | 391 | 30.9 | 86 | 50.0 | 44.57 | -76.32 | 3,4,9 | Identical dataset | (*24*, *25*) |
| *Junco hyemalis* | dark-eyed junco | 233 | 29.6 | - | - | 37.37 | -80.53 | 1,8,9 | Experimental | (*26*) |
| *Junco hyemalis* | dark-eyed junco | 187 | 28.3 | 38 | 34.2 | 37.37 | -80.53 | 1,8,9 | Identical dataset | (*27*) |
| *Junco hyemalis* | dark-eyed junco | - | - | 41 | 43.9 | 37.37 | -80.53 | 1,8,9 | Identical dataset | (*28*) |
| *Lamprotornis superbus* | superb starling | 247 | 13.8 | 100 | 25.0 | 0.12 | 37.87 | 1,9 | Identical dataset | (*29*) |
| *Luscinia svecica* | bluethroat | 150 | 20.0 | 31 | 35.5 | 62.42 | 8.87 | 6,7,3,11,12 | Identical dataset | (*30*) |
| *Luscinia svecica* | bluethroat | 721 | 29.0 | 136 | 47.1 | 62.42 | 8.87 | 6,7,3,11,12 | Identical dataset | (*31*) |
| *Luscinia svecica* | bluethroat | 479 | 27.8 | 86 | 59.3 | 62.42 | 8.87 | 6,7,3,11,12 | Identical dataset | (*32*) |
| *Luscinia svecica* | bluethroat | 1032 | 25.9 | 188 | 50.0 | 62.42 | 8.87 | 6,7,3,11,12 | Identical dataset | (*33*) |
| *Luscinia svecica* | bluethroat | 142 | 16.2 | 25 | 44.0 | 62.42 | 8.87 | 6,7,3,11,12 | Identical dataset, control dataset | (*34*) |
| *Luscinia svecica* | bluethroat | 162 | 30.2 | 31 | 58.1 | 62.42 | 8.87 | 6,7,3,11,12 | Identical dataset, control dataset from figure | (*35*) |
| *Malurus cyaneus* | superb fairy-wren | 309 | 62.1 | - | - | -35.27 | 149.10 | 6,8,9 | Identical dataset | (*36*) |
| *Malurus cyaneus* | superb fairy-wren | 114 | 69.3 | 35 | 82.9 | -35.27 | 149.10 | 6,8,9 | Identical dataset | (*37*) |
| *Malurus cyaneus* | superb fairy-wren | 1895 | 60.7 | - | - | -35.27 | 149.10 | 6,8,9 | Identical dataset | (*38*) |
| *Malurus cyaneus* | superb fairy-wren | - | - | 414 | - | -35.27 | 149.10 | 6,8,9 | Identical dataset, %EPP only for assigned offspring | (*39*) |
| *Malurus cyaneus* | superb fairy-wren | 181 | 76.2 | 40 | 95.0 | -35.27 | 149.10 | 6,8,9 | Identical dataset | (*40*) |
| *Malurus elegans* | red-winged fairy-wren | 261 | 56.7 | 118 | 70.3 | -34.35 | 116.02 | 8,10, 6 | Identical dataset | (*41*) |
| *Malurus melanocephalus* | red-backed fairy-wren | 186 | 47.3 | 73 | 60.3 | -27.27 | 152.85 | 1,2,4 | experimental | (*42*) |
| *Melanerpes formicivorus* | acorn woodpecker | 51 | 0.0 | 19 | 0.0 | 36.37 | -121.05 | - | Identical dataset | (*43*) |
| *Melospiza melodia* | song sparrow | 2667 | 28.2 | - | - | 48.63 | -123.02 | 1,3 | Identical dataset | (*44*) |
| *Melospiza melodia* | song sparrow | 2207 | 28.4 | 811 | 43.8 | 48.63 | -123.02 | 1,3 | Identical dataset | (*45*–*48*) |
| *Molothrus ater* | brown-headed cowbird | 43 | 4.7 | 12 | 0.0 | 50.18 | -98.38 | 1,4,9 | Identical dataset | (*49*) |
| *Parus ater* | coal tit | 3143 | 31.7 | 431 | 71.7 | 52.45 | 7.25 | 1,8,9 | Identical dataset | (*50*) |
| *Parus ater* | coal tit | 3365 | 31.4 | 457 | 70.5 | 52.45 | 7.25 | 1,8,9 | Identical dataset | (*51*) |
| *Parus atricapillus & Poecile carolinensis* | chickadees hybrids between black-capped & Carolina | 477 | 26.4 | 90 | 55.6 | 40.28 | -75.97 | - | hybrid | (*52*) |
| *Parus caeruleus* | blue tit | 457 | 4.8 | 46 | 32.6 | 58.87 | 9.60 | 1,6,9 | Identical dataset | (*53*) |
| *Parus caeruleus* | blue tit | 314 | 10.5 | 36 | 30.6 | 51.25 | 4.47 | 1,6,9 | Identical dataset | (*54*) |
| *Parus caeruleus* | blue tit | 245 | 15.9 | 20 | 75.0 | 48.22 | 16.33 | 1,6,9 | Identical dataset | (*55*) |
| *Parus caeruleus* | blue tit | 505 | 13.5 | 48 | 58.3 | 48.22 | 16.33 | 1,6,9 | Identical dataset | (*56*) |
| *Parus caeruleus* | blue tit | 574 | 15.5 | 51 | 64.7 | 48.22 | 16.33 | 1,6,9 | Identical dataset | (*57*) |
| *Parus caeruleus* | blue tit | 427 | 14.1 | 50 | 46.0 | 43.67 | 3.67 | 1,6,9 | Identical dataset | (*58*) |
| *Parus caeruleus* | blue tit | 395 | 16.5 | 42 | 59.5 | 43.67 | 3.67 | 1,6,9 | Experimental, high density | (*59*) |
| *Parus caeruleus* | blue tit | 443 | 11.3 | 49 | 42.9 | 43.67 | 3.67 | 1,6,9 | Experimental, low density | (*59*) |
| *Parus caeruleus* | blue tit | - | - | 396 | 46.0 | 48.13 | 10.88 | 1,6,9 | Identical dataset | (*60*) |
| *Parus caeruleus* | blue tit | - | - | 561 | 57.0 | 48.22 | 16.33 | 1,6,9 | Identical dataset | (*60*) |
| *Parus caeruleus* | blue tit | 452 | 11.3 | 43 | 51.2 | 53.13 | 6.58 | 1,6,9 | experimental | (*61*) |
| *Parus caeruleus* | blue tit | 673 | 8.2 | 72 | 41.7 | 57.05 | 18.28 | 1,6,9 | experimental | (*62*) |
| *Parus caeruleus* | blue tit | 175 | 13.1 | 28 | 46.4 | 59.98 | 10.77 | 1,6,9 | experimental | (*53*) |
| *Parus major* | great tit | 4018 | 11.0 | 668 | - | 47.97 | 11.23 | 1,8,9 | Identical dataset | (*63*) |
| *Parus montanus* | willow tit | 273 | 10.6 | 40 | 35.0 | 65.00 | 25.50 | 1,8 | Identical dataset | (*64*) |
| *Passer domesticus* | house sparrow | 185 | 10.3 | 56 | 28.6 | 35.00 | -83.00 | 3,9 | Identical dataset | (*65*) |
| *Passer domesticus* | house sparrow | 3194 | 17.4 | - | - | 51.00 | -4.00 | 3,9 | Identical dataset | (*66*) |
| *Passerculus sandwichensis* | savannah sparrow | - | 47.0 | 13 | 69.2 | 44.00 | -72.00 | 4,14,9 | Identical dataset | (*67*) |
| *Perisoreus infaustus* | Siberian jay | 20 | 0.0 | 15 | 0.0 | 62.37 | 21.50 | 8 | Identical dataset | (*68*) |
| *Phylloscopus fuscatus* | dusky warbler | 190 | 46.3 | 45 | 60.0 | 59.85 | 154.02 | 8,3,6,11,12 | Identical dataset | (*69*) |
| *Pipilo crissalis* | California towhee | 81 | 25.9 | 31 | 41.9 | 36.37 | -121.05 | 6,9 | experimental | (*70*) |
| *Prosthemadera novaeseelandiae* | tui | 152 | 57.2 | 53 | 71.7 | -36.37 | 174.83 | 6,8,7,9 | Identical dataset | (*71*) |
| *Pseudopodoces humilis* | ground tit | 438 | 13.0 | 77 | 37.7 | 34.23 | 102.03 | 4,14,9 | Identical dataset | (*72*) |
| *Pyrocephalus rubinus* | vermilion flycatcher | 34 | 47.1 | 11 | 63.6 | 19.28 | -98.23 | 1,6,9,2 | results depend on methods used, socially monogamous but both IBP and EPM recorded | (*73*) |
| *Setophaga ruticilla* | American redstart | 135 | 32.6 | 44 | 50.0 | 44.57 | -76.32 | 1,6,8 | Identical dataset | (*74*) |
| *Sialia sialis* | Eastern bluebird | 305 | 11.1 | 79 | 26.6 | 37.67 | -84.25 | 1,2,3,9 | experimental | (*75*) |
| *Spizella pusilla* | field sparrow | 1051 | 0.9 | 338 | 1.2 | 41.43 | -75.57 | 1,6,9 | Non-peer reviewed | (*76*) |
| *Sturnus vulgaris* | common starling | 92 | 8.7 | 22 | 31.8 | 55.72 | 13.45 | 1,3,9 | Identical dataset | (*77*) |
| *Sula nebouxii* | blue-footed booby | 810 | 6.4 | 463 | - | 21.85 | -105.89 | - | Identical dataset | (*78*) |
| *Tachycineta bicolor* | tree swallow | 235 | 48.5 | 46 | 78.3 | 43.38 | -88.02 | 3,12 | Experimental | (*79*) |
| *Tachycineta bicolor* | tree swallow | 192 | 52.6 | 39 | 84.6 | 43.38 | -88.02 | 3,12 | Identical dataset | (*80*) |
| *Tachycineta bicolor* | tree swallow | 104 | 51.0 | 21 | 76.2 | 44.57 | -76.32 | 3,12 | Identical dataset | (*81*) |
| *Tachycineta bicolor* | tree swallow | 448 | 51.1 | 67 | 73.1 | 44.57 | -76.32 | 3,12 | Identical dataset | (*82*) |
| *Tachycineta bicolor* | tree swallow | 119 | 52.9 | 23 | 87.0 | 44.57 | -76.32 | 3,12 | Identical dataset | (*83*) |
| *Tachycineta bicolor* | tree swallow | 181 | 50.8 | 34 | 67.6 | 44.57 | -76.32 | 3,12 | Identical dataset | (*84*) |
| *Tachycineta bicolor* | tree swallow | 193 | 52.3 | 34 | 85.3 | 44.57 | -76.32 | 3,12 | Identical dataset | (*85*) |
| *Tachycineta bicolor* | tree swallow | 276 | 32.6 | 55 | 56.4 | 44.57 | -76.32 | 3,12 | Identical dataset | (*86*) |
| *Tachycineta bicolor* | tree swallow | 44 | 77.3 | 12 | 100.0 | 44.57 | -76.32 | 3,12 | Identical dataset, estimates from polygynous males | (*87*) |
| *Tachycineta bicolor* | tree swallow | 86 | 38.4 | 16 | 50.0 | 44.57 | -76.32 | 3,12 | Identical dataset | (*88*) |
| *Tachycineta bicolor* | tree swallow | 341 | 46.0 | 72 | 63.9 | 44.63 | -76.27 | 3,12 | Identical dataset | (*89*) |
| *Troglodytes aedon* | house wren | 584 | 10.1 | - | - | 43.38 | -88.02 | 1,3,8,9 | Identical dataset | (*90*) |
| *Troglodytes aedon* | house wren | 138 | 15.2 | 34 | 32.4 | -36.43 | -56.94 | 1,3,8,9 | Identical dataset | (*91*) |
| *Troglodytes aedon* | house wren | 158 | 17.7 | 30 | 43.3 | 42.52 | -76.47 | 1,3,8,9 | Identical dataset | (*91*) |
| *Troglodytes aedon* | house wren | 2345 | 14.9 | 476 | 36.6 | 40.67 | -88.88 | 1,3,8,9 | Identical dataset | (*92*) |
| *Troglodytes aedon* | house wren | 1466 | 16.6 | 283 | 35.0 | 40.67 | -88.88 | 1,3,8,9 | Identical dataset | (*93*) |
| *Tyrannus tyrannus* | Eastern kingbird | 132 | 40.9 | 45 | 57.8 | 43.00 | -119.00 | 1,3,8,9,15 | Identical dataset | (*94*) |
| *Tyto alba* | barn owl | 211 | 0.5 | 54 | 1.9 | 46.82 | 6.93 | - | Identical dataset | (*95*) |
| *Vermivora chrysoptera x V. pinus* | golden-winged warblers & golden-winged X blue-winged | 236 | 31.8 | 54 | 55.6 | 44.57 | -76.32 | 1,6 | hybrid | (*96*) |

**References**

1. O. Rätti, A. Lundberg, H. Tegelström, R. V. Alatalo, No evidence for effects of breeding density and male removal on extrapair paternity in the pied flycatcher. *The Auk*. **118**, 147–155 (2001).

2. K. L. Buchanan, C. K. Catchpole, Extra-pair paternity in the socially monogamous Sedge Warbler Acrocephalus schoenobaenus as revealed by multilocus DNA fingerprinting. *Ibis*. **142**, 12–20 (2000).

3. D. F. Westneat, H. L. Mays, Tests of spatial and temporal factors influencing extra-pair paternity in red-winged blackbirds: factors affecting EPP. *Mol. Ecol.* **14**, 2155–2167 (2005).

4. J. A. Eimes, P. G. Parker, J. L. Brown, E. R. Brown, Extrapair fertilization and genetic similarity of social mates in the Mexican jay. *Behav. Ecol.* **16**, 456–460 (2005).

5. J. Schnitzer *et al.*, Male ornamentation and within-pair paternity are not associated with male provisioning rates in scarlet rosefinches Carpodacus erythrinus. *Acta Ethologica*. **17**, 89–97 (2014).

6. M. Promerová *et al.*, Occurrence of extra-pair paternity is connected to social male’s MHC-variability in the scarlet rosefinch Carpodacus erythrinus. *J. Avian Biol.* **42**, 5–10 (2011).

7. T. Albrecht *et al.*, Extra-pair fertilizations contribute to selection on secondary male ornamentation in a socially monogamous passerine. *J. Evol. Biol.* **22**, 2020–2030 (2009).

8. T. Albrecht *et al.*, Extrapair paternity and the opportunity for sexual selection in long-distant migratory passerines. *Behav. Ecol.* **18**, 477–486 (2007).

9. K. P. Oh, A. V. Badyaev, Adaptive genetic complementarity in mate choice coexists with selection for elaborate sexual traits. *Proc. R. Soc. B Biol. Sci.* **273**, 1913–1919 (2006).

10. D. Blomqvist *et al.*, Genetic similarity between mates and extra-pair parentage in three species of shorebirds. *Nature*. **419**, 613–615 (2002).

11. S. A. Kaiser, T. S. Sillett, B. B. Risk, M. S. Webster, Experimental food supplementation reveals habitat-dependent male reproductive investment in a migratory bird. *Proc. R. Soc. B Biol. Sci.* **282**, 20142523–20142523 (2015).

12. S. A. Kaiser, B. B. Risk, T. S. Sillett, M. S. Webster, Ecological and Social Factors Constrain Spatial and Temporal Opportunities for Mating in a Migratory Songbird. *Am. Nat.* **189**, 283–296 (2017).

13. S. M. Yezerinac, P. J. Weatherhead, P. T. Boag, Extra-pair paternity and the opportunity for sexual selection in a socially monogamous bird (Dendroica petechia). *Behav. Ecol. Sociobiol.* **37**, 179–188 (1995).

14. E. L. Taylor, D. Blache, D. Groth, J. D. Wetherall, G. B. Martin, Genetic evidence for mixed parentage in nests of the emu (Dromaius novaehollandiae). *Behav. Ecol. Sociobiol.* **47**, 359–364 (2000).

15. S. M. Suter, D. Ermacora, N. Rieille, D. R. Meyer, A distinct reed bunting dawn song and its relation to extrapair paternity. *Anim. Behav.* **77**, 473–480 (2009).

16. J. J. Negro *et al.*, DNA fingerprinting reveals a low incidence of extra-pair fertilizations in the lesser kestrel. *Anim. Behav.* **51**, 935–943 (1996).

17. D. Canal, J. Dávila, J. Potti, Male phenotype predicts extra-pair paternity in pied flycatchers. *Behaviour*. **148**, 691–712 (2011).

18. S. R. Beissinger, Long-term studies of the green-rumped parrotlet (forpus passerinus) in venezuela: hatching asynchrony, social system and population structure. *Ornitol. Neotropical*. **19**, 73–84 (2008).

19. L. F. Keller, P. R. Grant, B. R. Grant, K. Petren, Heritability of morphological traits in Darwin’s Finches: misidentified paternity and maternal effects. *Heredity*. **87**, 325–336 (2001).

20. K. Petren, B. R. Grant, P. R. Grant, Low extrapair paternity in the cactus finch (Geospiza scandens). *Auk*. **116**, 252–256 (1999).

21. K. J. Thusius, Extrapair paternity is influenced by breeding synchrony and density in the common yellowthroat. *Behav. Ecol.* **12**, 633–639 (2001).

22. M. C. Pedersen, P. O. Dunn, L. A. Whittingham, Extraterritorial forays are related to a male ornamental trait in the common yellowthroat. *Anim. Behav.* **72**, 479–486 (2006).

23. C. R. Primmer, A. P. MøLler, H. Ellegren, Resolving genetic relationships with microsatellite markers: a parentage testing system for the swallow Hirundo rustica. *Mol. Ecol.* **4**, 493–498 (1995).

24. O. Kleven, F. Jacobsen, R. Izadnegahdar, R. J. Robertson, J. T. Lifjeld, Male tail streamer length predicts fertilization success in the North American barn swallow (Hirundo rustica erythrogaster). *Behav. Ecol. Sociobiol.* **59**, 412–418 (2006).

25. O. Kleven, F. Jacobsen, R. Izadnegahdar, R. J. Robertson, J. T. Lifjeld, No evidence of paternal genetic contribution to nestling cell-mediated immunity in the North American barn swallow. *Anim. Behav.* **71**, 839–845 (2006).

26. J. W. McGlothlin *et al.*, Natural selection on testosterone production in a wild songbird population. *Am. Nat.* **175**, 687–701 (2010).

27. E. D. Ketterson *et al.*, The Relative Impact of Extra-Pair Fertilizations on Variation in Male and Female Reproductive Success in Dark-Eyed Juncos (Junco hyemalis). *Ornithol. Monogr.*, 81–101 (1998).

28. N. M. Gerlach, E. D. Ketterson, Experimental elevation of testosterone lowers fitness in female dark-eyed juncos. *Horm. Behav.* **63**, 782–790 (2013).

29. D. R. Rubenstein, Female extrapair mate choice in a cooperative breeder: trading sex for help and increasing offspring heterozygosity. *Proc. R. Soc. B Biol. Sci.* **274**, 1895–1903 (2007).

30. C. Krokene, K. Anthonisen, J. T. Lifjeld, T. Amundsen, Paternity and paternity assurance behaviour in the bluethroat, Luscinia s svecica. *Anim. Behav.* **52**, 405–417 (1996).

31. A. Johnsen, J. T. Lifjeld, S. Andersson, J. Ornborg, T. Amundsen, Male characteristics and fertilisation success in bluethroats. *Behaviour*. **138**, 1371–1390 (2001).

32. A. Johnsen, V. Andersen, C. Sunding, J. T. Lifjeld, Female bluethroats enhance offspring immunocompetence through extra-pair copulations. *Nature*. **406**, 296–299 (2000).

33. F. Fossøy, A. Johnsen, J. T. Lifjeld, Multiple genetic benefits of female promiscuity in a socially monogamous passerine. *Evolution*. **62**, 145–156 (2008).

34. A. Johnsen *et al.*, Is female promiscuity constrained by the presence of her social mate? An experiment with bluethroats Luscinia svecica. *Behav. Ecol. Sociobiol.* **62**, 1761–1767 (2008).

35. A. Johnsen, J. T. Lifjeld, P. A. Rohde, C. R. Primmer, H. Ellegren, Sexual conflict over fertilizations: female bluethroats escape male paternity guards. *Behav. Ecol. Sociobiol.* **43**, 401–408 (1998).

36. P. O. Dunn, A. Cockburn, Extrapair mate choice and honest signaling in cooperatively breeding superb fairy-wrens. *Evolution*. **53**, 938–946 (1999).

37. P. O. Dunn, A. Cockburn, Evolution of male parental care in a bird with almost complete cuckoldry. *Evolution*. **50**, 2542–2548 (1996).

38. M. C. Double, A. Cockburn, Subordinate superb fairy-wrens (Malurus cyaneus) parasitize the reproductive success of attractive dominant males. *Proc. R. Soc. B Biol. Sci.* **270**, 379–384 (2003).

39. M. Double, A. Cockburn, Pre-dawn infidelity: females control extra-pair mating in superb fairy-wrens. *Proc. R. Soc. Lond. Ser. B-Biol. Sci.* **267**, 465–470 (2000).

40. R. A. Mulder, P. O. Dunn, A. Cockburn, K. A. Lazenbycohen, M. J. Howell, Helpers liberate female fairy-wrens from constraints on extra-pair mate choice. *Proc. R. Soc. Lond. Ser. B-Biol. Sci.* **255**, 223–229 (1994).

41. L. Brouwer, M. van de Pol, E. Atema, A. Cockburn, Strategic promiscuity helps avoid inbreeding at multiple levels in a cooperative breeder where both sexes are philopatric. *Mol. Ecol.* **20**, 4796–4807 (2011).

42. D. T. Baldassarre, E. I. Greig, M. S. Webster, The couple that sings together stays together: duetting, aggression and extra-pair paternity in a promiscuous bird species. *Biol. Lett.* **12**, 20151025 (2016).

43. J. Dickinson, J. Haydock, W. Koenig, M. Stanback, F. Pitelka, Genetic monogamy in single-male groups of acorn woodpeckers, Melanerpes formicivorus. *Mol. Ecol.* **4**, 765–770 (1995).

44. J. M. Reid, P. Arcese, L. F. Keller, S. Losdat, Female and male genetic effects on offspring paternity: additive genetic (co)variances in female extra-pair reproduction and male paternity success in song sparrows (Melospiza melodia). *Evolution*. **68**, 2357–2370 (2014).

45. R. J. Sardell, L. F. Keller, P. Arcese, T. Bucher, J. M. Reid, Comprehensive paternity assignment: genotype, spatial location and social status in song sparrows, Melospiza Melodia. *Mol. Ecol.* **19**, 4352–4364 (2010).

46. C. Lebigre, P. Arcese, J. M. Reid, Decomposing variation in male reproductive success: age-specific variances and covariances through extra-pair and within-pair reproduction. *J. Anim. Ecol.* **82**, 872–883 (2013).

47. J. M. Reid, R. J. Sardell, Indirect selection on female extra-pair reproduction? Comparing the additive genetic value of maternal half-sib extra-pair and within-pair offspring. *Proc. R. Soc. B Biol. Sci.* **279**, 1700–1708 (2012).

48. J. M. Reid, P. Arcese, R. J. Sardell, L. F. Keller, Additive Genetic Variance, Heritability, and Inbreeding Depression in Male Extra-Pair Reproductive Success. *Am. Nat.* **177**, 177–187 (2011).

49. G. W. Alderson, H. L. Gibbs, S. G. Sealy, Determining the reproductive behaviour of individual brown-headed cowbirds using microsatellite DNA markers. *Anim. Behav.* **58**, 895–905 (1999).

50. T. Schmoll *et al.*, Paternal genetic effects on offspring fitness are context dependent within the extrapair mating system of a socially monogamous passerine. *Evolution*. **59**, 645 (2005).

51. V. Dietrich-Bischoff, T. Schmoll, W. Winkel, S. Krackow, T. Lubjuhn, Extra-pair paternity, offspring mortality and offspring sex ratio in the socially monogamous coal tit (Parus ater). *Behav. Ecol. Sociobiol.* **60**, 563–571 (2006).

52. M. W. Reudink, S. G. Mech, R. L. Curry, Extrapair paternity and mate choice in a chickadee hybrid zone. *Behav. Ecol.* **17**, 56–62 (2006).

53. C. Krokene, J. T. Lifjeld, Variation in the frequency of extra-Pair paternity in birds: a comparison of an island and a mainland population of blue tits. *Behaviour*. **137**, 1317–1330 (2000).

54. B. Kempenaers, G. R. Verheyen, M. Van den Broeck, C. Van Broeckhoven, A. A. Dhondt, Extra-pair paternity results from female preference for high-quality males in the blue tit. *Nature*. **357**, 494–496 (1992).

55. A. Johnsen *et al.*, Laying-order effects on sperm numbers and on paternity: comparing three passerine birds with different life histories. *Behav. Ecol. Sociobiol.* **66**, 181–190 (2012).

56. A. Johnsen, K. Delhey, S. Andersson, B. Kempenaers, Plumage colour in nestling blue tits: sexual dichromatism, condition dependence and genetic effects. *Proc. R. Soc. Lond. B Biol. Sci.* **270**, 1263–1270 (2003).

57. K. Delhey, A. Johnsen, A. Peters, S. Andersson, B. Kempenaers, Paternity analysis reveals opposing selection pressures on crown coloration in the blue tit (Parus caeruleus). *Proc. R. Soc. Lond. Ser. B-Biol. Sci.* **270**, 2057–2063 (2003).

58. A. Charmantier, C. Blondel, A contrast in extra-pair paternity levels on mainland and island populations of mediterranean blue tits. *Ethology*. **109**, 351–363 (2003).

59. A. Charmantier, P. Perret, Manipulation of nest-box density affects extra-pair paternity in a population of blue tits (Parus caeruleus). *Behav. Ecol. Sociobiol.* **56**, 360–365 (2004).

60. L. Schlicht, M. Valcu, B. Kempenaers, Spatial patterns of extra-pair paternity: beyond paternity gains and losses. *J. Anim. Ecol.* **84**, 518–531 (2015).

61. J. E. Brommer, P. Korsten, K. M. Bouwman, M. L. Berg, J. Komdeur, Is extrapair mating random? On the probability distribution of extrapair young in avian broods. *Behav. Ecol.* **18**, 895–904 (2007).

62. A. Arct, S. M. Drobniak, E. Podmokła, L. Gustafson, M. Cichoń, Benefits of extra-pair mating may depend on environmental conditions—an experimental study in the blue tit (Cyanistes caeruleus). *Behav. Ecol. Sociobiol.* **67**, 1809–1815 (2013).

63. Y. G. Araya-Ajoy, N. J. Dingemanse, B. Kempenaers, Timing of extrapair fertilizations: within-pair fertilization trade-offs or pair synchrony spillovers? *Behav. Ecol.* **27**, 377–384 (2016).

64. S. Rytkönen, L. Kvist, R. Mikkonen, M. Orell, Intensity of nest defence is not related to degree of paternity in the willow tit Parus montanus. *J. Avian Biol.* **38**, 273–277 (2007).

65. S. C. Griffith, I. R. K. Stewart, D. A. Dawson, I. P. F. Owens, T. Burke, Contrasting levels of extra-pair paternity in mainland and island populations of the house sparrow (Passer domesticus): is there an ?island effect?? *Biol. J. Linn. Soc.* **68**, 303–316 (1999).

66. Y.-H. Hsu *et al.*, Age-dependent trajectories differ between within-pair and extra-pair paternity success. *J. Evol. Biol.* **30**, 951–959 (2017).

67. N. G. Perlut, L. M. Kelly, N. J. Zalik, A. M. Strong, Male savannah sparrows provide less parental care with increasing paternity loss. *Northeast. Nat.* **19**, 335–344 (2012).

68. B.-G. Lillandt, S. Bensch, T. von Schantz, Family structure in the Siberian jay as revealed by microsatellite analyses. *The Condor*. **105**, 505–514 (2003).

69. W. Forstmeier, Extra-pair paternity in the dusky warbler, Phylloscopus fuscatus: A test of the “constrained female hypothesis.” *Behaviour*. **140**, 1117–1134 (2003).

70. L. Benedict, Unusually high levels of extrapair paternity in a duetting songbird with long-term pair bonds. *Behav. Ecol. Sociobiol.* **62**, 983–988 (2008).

71. S. J. Wells, W. Ji, D. Gleeson, B. Jones, J. Dale, Population social structure facilitates indirect fitness benefits from extra-pair mating. *Front. Ecol. Evol.* **5** (2017), doi:10.3389/fevo.2017.00018.

72. B. Du, X. Lu, Bi-parental vs. cooperative breeding in a passerine: fitness-maximizing strategies of males in response to risk of extra-pair paternity? *Mol. Ecol.* **18**, 3929–3939 (2009).

73. A. A. Ríos-Chelén *et al.*, Intra-specific brood parasitism revealed by DNA micro-satellite analyses in a sub-oscine bird, the vermilion flycatcher. *Rev. Chil. Hist. Nat.* **81** (2008), doi:10.4067/S0716-078X2008000100002.

74. M. W. Reudink *et al.*, Non-breeding season events influence sexual selection in a long-distance migratory bird. *Proc. R. Soc. B Biol. Sci.* **276**, 1619–1626 (2009).

75. S. L. M. Stewart, D. F. Westneat, G. Ritchison, Extra-pair paternity in eastern bluebirds: effects of manipulated density and natural patterns of breeding synchrony. *Behav. Ecol. Sociobiol.* **64**, 463–473 (2010).

76. M. Carey, D. E. Burhans, D. A. Nelson, Field Sparrow (Spizella pusilla). *Birds N. Am. Online* (2008), doi:10.2173/bna.103.

77. H. G. Smith, T. von Schantz, Extra-pair paternity in the European starling: the effect of polygyny. *Condor*. **95**, 1006–1015 (1993).

78. L. M. Kiere, A. G. Ramos, H. Drummond, No evidence that genetic compatibility drives extra-pair behavior in female blue-footed boobies. *J. Avian Biol.* **47**, 871–879 (2016).

79. L. A. Whittingham, P. O. Dunn, M. K. Stapleton, Repeatability of extra-pair mating in tree swallows. *Mol. Ecol.* **15**, 841–849 (2006).

80. L. A. Whittingham, P. O. Dunn, E. D. Clotfelter, Parental allocation of food to nestling tree swallows: the influence of nestling behaviour, sex and paternity. *Anim. Behav.* **65**, 1203–1210 (2003).

81. B. Kempenaers, S. Everding, C. Bishop, P. Boag, R. J. Robertson, Extra-pair paternity and the reproductive role of male floaters in the Tree swallow (Tachycineta bicolor). *Behav. Ecol. Sociobiol.* **49**, 251–259 (2001).

82. B. Kempenaers, B. Congdon, P. Boag, R. J. Robertson, Extrapair paternity and egg hatchability in tree swallows: evidence for the genetic compatibility hypothesis? *Behav. Ecol.* **10**, 304–311 (1999).

83. P. O. Dunn, et al., Extra-pair paternity in tree swallows: why do females mate with more than one male? *Behav. Ecol. Sociobiol.* **35**, 273–281 (1994).

84. P. O. Dunn, L. A. Whittingham, J. T. Lifjeld, R. J. Robertson, P. T. Boag, Effects of breeding density, synchrony, and experience on extra-pair paternity in tree swallows. *Behav. Ecol.* **57**, 1071–1081 (1994).

85. S. A. Crowe *et al.*, Paternity assurance through frequent copulations in a wild passerine with intense sperm competition. *Anim. Behav.* **77**, 183–187 (2009).

86. C. A. Barber, R. J. Robertson, Timing of Copulations and the Pattern of Paternity in Relation to Laying Order in Tree Swallows Tachycineta bicolor. *J. Avian Biol.* **38**, 249–254 (2007).

87. P. O. Dunn, R. J. Robertson, Extra-pair paternity in polygynous tree swallows. *Anim. Behav.* **45**, 231–239 (1993).

88. J. T. Lifjeld, P. O. Dunn, R. J. Robertson, P. T. Boag, Extra-pair paternity in monogamous tree swallows. *Anim. Behav.* **45**, 213–229 (1993).

89. C. A. Barber, M. J. Edwards, R. J. Robertson, A test of the genetic compatibility hypothesis with tree swallows, *Tachycineta bicolor*. *Can. J. Zool.* **83**, 955–961 (2005).

90. N. E. Poirier, L. A. Whittingham, P. O. Dunn, Males achieve greater reproductive success through multiple broods, than through extrapair mating in house wrens. *Anim. Behav.* **67**, 1109–1116 (2004).

91. K. LaBarbera, I. J. Lovette, P. E. Llambías, Mating opportunities, paternity, and sexual conflict: paternal care in northern and southern temperate house wrens. *Behav. Ecol. Sociobiol.* **66**, 253–260 (2012).

92. L. S. Johnson *et al.*, Extra-pair young in house wren broods are more likely to be male than female. *Proc. R. Soc. B Biol. Sci.* **276**, 2285–2289 (2009).

93. A. M. Forsman *et al.*, Female house wrens ( *Troglodytes aedon* ) increase the size, but not immunocompetence, of their offspring through extra-pair mating. *Mol. Ecol.* **17**, 3697–3706 (2008).

94. C. M. Chutter *et al.*, Paternal behaviour in a socially monogamous but sexually promiscuous passerine bird. *Behaviour*. **153**, 443–466 (2016).

95. A. Roulin *et al.*, Extra-Pair Paternity, Testes Size and Testosterone Level in Relation to Colour Polymorphism in the Barn Owl Tyto alba. *J. Avian Biol.* **35**, 492–500 (2004).

96. R. Vallender, V. L. Friesen, R. J. Robertson, Paternity and performance of golden-winged warblers (Vermivora chrysoptera) and golden-winged X blue-winged warbler (V. pinus) hybrids at the leading edge of a hybrid zone. *Behav. Ecol. Sociobiol.* **61**, 1797–1807 (2007).
