# Supplementary material for: Extra‐pair paternity in birds
Source: Mol Ecol. 2019 Oct 31;28(22):4864–82. doi: 10.1111/mec.15259 (PMC6899757; doi:10.1111/mec.15259)
Supplement: Supplementary file 4 [file MEC-28-4864-s004.docx]

**Supplemental Information for:**

**Extra-pair paternity in birds**

Lyanne Brouwer^1,2,3*^ & Simon C. Griffith^4^

^1^Department of Animal Ecology & Physiology, Institute for Water and Wetland Research, Radboud University, Nijmegen, The Netherlands

^2^Department of Animal Ecology, Netherlands Institute of Ecology (NIOO-KNAW), Wageningen, The Netherlands

^3^Division of Ecology and Evolution, Research School of Biology, The Australian National University, Canberra ACT 2601, Australia

^4^Department of Biological Sciences, Macquarie University, North Ryde, NSW 2109, Australia

**Table S4**. Discrepancies between our data-set and a dataset by Cornwallis et al. 2017 (1) in the number of broods (Nbr) or proportion of broods with at least one extra-pair offspring (EPbr) indicating the difficulties in extracting data from the literature.

|  | This study | | Cornwallis et al. 2017 | |
| --- | --- | --- | --- | --- |
| Reference | Nbr | EPbr | Nbr | EPbr |
| (*2*) | 29 | 6.9 | 32 | 10.3 |
| (*3*) | 6 | 0.0 | 13 | 0.0 |
| (*4*) | 121 | 73.6 | 89 | 73.5 |
| (*5*) | 10 | 0.0 | 32 | 0.0 |
| (*6*) | 19 | 52.6 | 19 | NA |
| (*7*) | 20 | 0.0 | 44 | 2.3 |
| (*8*) | 414 | NA | 253 | 65.2 |
| (*9*) | 47 | 63.8 | 66 | 45.5 |
| (*10*) | 36 | 66.7 | 136 | 17.6 |
| (*11*) | 44 | 2.3 | 54 | 0.0 |
| (*12*) | 60 | 95.0 | 50 | 95.0 |
| (*13*) | 52 | 26.9 | 143 | 51.8 |
| (*14*) | 72 | 54.2 | 39 | 54.2 |
| (*15*) | 164 | 12.8 | 96 | 10.4 |
| (*16*) | 122 | 46.7 | 36 | 55.6 |
| (*17*) | 68 | 1.5 | 74 | 9.5 |
| (*18*) | 38 | 0.0 | 38 | 7.5 |
| (*19*) | 99 | 82.8 | 50 | 92.0 |
| (*20*) | 54 | 1.9 | 54 | 0.0 |
| (*21*) | 82 | 8.5 | 69 | 10.1 |
| (*22*) | 203 | NA | 134 | 53.7 |
| (*23*) | 159 | 55.3 | 183 | 29.5 |

**References**

1. C. K. Cornwallis *et al.*, Cooperation facilitates the colonization of harsh environments. *Nat. Ecol. Evol.* **1**, 0057 (2017).

2. C. L. Abbott, M. C. Double, R. Gales, A. Cockburn, Copulation behaviour and paternity in shy albatrosses (Thalassarche cauta). *J. Zool.* **270**, 628–635 (2006).

3. M. M. Baumgarten, A. B. Kohlrausch, C. Yumimiyaki, T. R. Ochotorena de Freitas, A. Mellender de Araujo, DNA Fingerprinting and Parentage in Masked (Sula Dactylatra) and Brown (S. Leucogaster) Boobies. *Ornitologia Neotropical*, 319–326 (2001).

4. K. M. Bouwman, T. Burke, J. Komdeur, How reed buntings benefit from extra-pair mating behaviour: testing hypotheses through patterns of paternity in sequential broods. *Mol. Ecol.* **15,** 2589-2600 (2006).

5. J. P. Bruce, J. S. Quinn, S. A. Sloane, B. N. White, DNA Fingerprinting Reveals Monogamy in the Bushtit, a Cooperatively Breeding Species. *The Auk*. **113**, 511–516 (1996).

6. I. Chiver, B. J. M. Stutchbury, E. S. Morton, The function of seasonal song in a tropical resident species, the Red-throated Ant-tanager (Habia fuscicauda). *J. Ornithol.* **156**, 55–63 (2015).

7. R. Covas, A. Dalecky, A. Caizergues, C. Doutrelant, Kin associations and direct vs indirect fitness benefits in colonial cooperatively breeding sociable weavers Philetairus socius. *Behav. Ecol. Sociobiol.* **60**, 323–331 (2006).

8. M. Double, A. Cockburn, Pre-dawn infidelity: females control extra-pair mating in superb fairy-wrens. *Proc. R. Soc. Lond. Ser. B-Biol. Sci.* **267**, 465–470 (2000).

9. R. Edler, T. W. P. Friedl, Within-pair young are more immunocompetent than extrapair young in mixed-paternity broods of the red bishop. *Anim. Behav.* **75**, 391–401 (2008).

10. M. L. Evans, B. E. Woolfenden, L. Friesen, B. J. M. Stutchbury, Variation in the extra-pair mating systems of Acadian Flycatchers and Wood Thrushes in forest fragments in southern Ontario. *J. Field Ornithol.* **80**, 146–153 (2009).

11. S. M. Haig, J. R. Walters, J. H. Plissner, Genetic evidence for monogamy in the cooperatively breeding red-cockaded woodpecker. *Behav. Ecol. Sociobiol.* **34**, 295–303 (1994).

12. C. E. Hill, C. Gjerdrum, C. S. Elphick, Extreme Levels of Multiple Mating Characterize the Mating System of the Saltmarsh Sparrow ( *Ammodramus caudacutus* ). *The Auk*. **127**, 300–307 (2010).

13. J. M. C. Hutchinson, S. C. Griffith, Extra-pair paternity in the Skylark Alauda arvensis: Extra-pair paternity in the Skylark. *Ibis*. **150**, 90–97 (2007).

14. O. Kleven, J. T. Lifjeld, No evidence for increased offspring heterozygosity from extrapair mating in the reed bunting (Emberiza schoeniclus). *Behav. Ecol.* **16**, 561–565 (2005).

15. T. Lubjuhn, W. Winkel, J. T. Epplen, J. Brün, Reproductive success of monogamous and polygynous pied flycatchers ( Ficedula hypoleuca ). *Behav. Ecol. Sociobiol.* **48**, 12–17 (2000).

16. M. J. L. Magrath, O. Vedder, M. van der Velde, J. Komdeur, Maternal Effects Contribute to the Superior Performance of Extra-Pair Offspring. *Curr. Biol.* **19**, 792–797 (2009).

17. S. Rodriguez-Martínez, M. Carrete, S. Roques, N. Rebolo-Ifrán, J. L. Tella, High Urban Breeding Densities Do Not Disrupt Genetic Monogamy in a Bird Species. *PLoS ONE*. **9**, e91314 (2014).

18. M. Stanback, D. S. Richardson, C. Boix-Hinzen, J. Mendelsohn, Genetic monogamy in Monteiro’s hornbill, Tockus monteiri. *Anim. Behav.* **63**, 787–793 (2002).

19. M. K. Stapleton, O. Kleven, J. T. Lifjeld, R. J. Robertson, Female tree swallows (Tachycineta bicolor) increase offspring heterozygosity through extrapair mating. *Behav. Ecol. Sociobiol.* **61**, 1725–1733 (2007).

20. S. S. Taylor, S. Boessenkool, I. G. Jamieson, Genetic monogamy in two long-lived New Zealand passerines. *J. Avian Biol.* **39**, 579–583 (2008).

21. N. Verboven, C. Mateman, Low frequency of extra-pair fertilizations in the great tit Parus major revealed by DNA fingerprinting. *J. Avian Biol.* **28**, 231–239 (1997).

22. P. J. Weatherhead, P. T. Boag, Pair and extra-pair mating success relative to male quality in red-winged blackbirds. *Behav. Ecol. Sociobiol.* **37**, 81–91 (1995).

23. M. S. Webster, K. A. Tarvin, E. M. Tuttle, S. Pruett-Jones, Reproductive promiscuity in the splendid fairy-wren: effects of group size and auxiliary reproduction. *Behav. Ecol.* **15**, 907–915 (2004).
