# Supplementary material for: Extra‐pair paternity in birds
Source: Mol Ecol. 2019 Oct 31;28(22):4864–82. doi: 10.1111/mec.15259 (PMC6899757; doi:10.1111/mec.15259)
Supplement: Supplementary file 5 [file MEC-28-4864-s005.docx]

**Supplemental Information for:**

**Extra-pair paternity in birds**

Lyanne Brouwer^1,2,3*^ & Simon C. Griffith^4^

^1^Department of Animal Ecology & Physiology, Institute for Water and Wetland Research, Radboud University, Nijmegen, The Netherlands

^2^Department of Animal Ecology, Netherlands Institute of Ecology (NIOO-KNAW), Wageningen, The Netherlands

^3^Division of Ecology and Evolution, Research School of Biology, The Australian National University, Canberra ACT 2601, Australia

^4^Department of Biological Sciences, Macquarie University, North Ryde, NSW 2109, Australia

**Table S5**. Average EPP rates for 328 sampled bird species, based on the studies included in Tables S1 and S2. Breeding system categories are as follows: 1 monogamy or polygyny, 2 polyandry (multiple males, single female), 3 polygynandry (multiple males and females), 4 cooperative breeder, 5 no social bond, 6 lekking, 7 unknown. Nesting vegetation type is given for socially monogamous passerines either nesting in reed type vegetation or forest (see SI for more details).

| Scientific name | Common name | N | EPP | Breeding system | Nesting vegetation |
| --- | --- | --- | --- | --- | --- |
| *Acanthiza pusilla* | brown thornbill | 178 | 6.2 | 1 | forest |
| *Accipiter cooperii* | Cooper's hawk | 140 | 19.3 | 1 |  |
| *Acrocephalus arundinaceus* | great reed warbler | 872 | 4.8 | 1 | reed |
| *Acrocephalus bistrigiceps* | black-browed reed-warbler | 140 | 6.4 | 1 | reed |
| *Acrocephalus melanopogon* | moustached warbler | 44 | 27.3 | 2 |  |
| *Acrocephalus paludicola* | aquatic warbler | 410 | 38.5 | 5 |  |
| *Acrocephalus palustris* | marsh warbler | 131 | 3.1 | 1 |  |
| *Acrocephalus schoenobaenus* | sedge warbler | 604 | 8.1 | 1 | reed |
| *Acrocephalus scirpaceus* | Eurasian reed-warbler | 186 | 6.5 | 1 | reed |
| *Acrocephalus sechellensis* | Seychelles warbler | 55 | 38.2 | 4 |  |
| *Acrocephalus vaughani* | Henderson reed-warbler | 28 | 7.1 | 1,2 |  |
| *Actitis hypoleucos* | common sandpiper | 136 | 12.5 | 1 |  |
| *Actitis macularius* | spotted sandpiper | 34 | 2.9 | 2 |  |
| *Aegithalos caudatus* | long-tailed tit | 372 | 1.9 | 4 |  |
| *Aegithalos concinnus* | black-throated tit | 383 | 5.7 | 4 |  |
| *Aegithalos glaucogularis* | silver-throated bush tit | 412 | 6.8 | 4 |  |
| *Aegolius funereus* | boreal owl | 109 | 0 | 1 |  |
| *Agelaius phoeniceus* | red-winged blackbird | 1523 | 27.8 | 1 | reed |
| *Agelaius xanthomus* | yellow-shouldered blackbird | 87 | 23 | 1 | forest |
| *Alauda arvensis* | skylark | 171 | 20.5 | 1 |  |
| *Alectura lathami* | Australian brush-turkey | 65 | 27.7 | 5 |  |
| *Alle alle* | little auk | 88 | 2.3 | 1 |  |
| *Ammodramus maritimus* | seaside sparrow | 47 | 10.6 | 1 | reed |
| *Anas platyrhynchos* | mallard | 204 | 9.3 | 1 |  |
| *Anas strepera* | gadwalls | 261 | 4.2 | 1 |  |
| *Anthoscopus minutus* | Cape pendulin tit | 42 | 7.1 | 4 |  |
| *Anthus spinoletta* | water pipit | 1052 | 5.2 | 1 |  |
| *Aphelocoma coerulescens* | Florida scrub-jay | 139 | 0 | 4 |  |
| *Aphelocoma ultramarina* | Mexican jay | 139 | 39.6 | 4 |  |
| *Apus apus* | common swift | 88 | 4.5 | 1 |  |
| *Aquila heliaca* | Eastern imperial eagle | 166 | 0 | 1 |  |
| *Ardea alba* | great egret | 50 | 0 | 1 |  |
| *Asio otus* | long-eared owl | 59 | 0 | 1 |  |
| *Athene cunicularia* | burrowing owl | 121 | 1.7 | 1 |  |
| *Athene noctua* | little owl | 53 | 0 | 1 |  |
| *Baeolophus bicolor* | tufted titmouse | 34 | 8.8 | 1 | forest |
| *Bartramia longicauda* | upland sandpiper | 177 | 18.1 | 1 |  |
| *Branta bernicla* | black brant | 374 | 5.6 | 1 |  |
| *Branta leucopsis* | barnacle goose | 153 | 0 | 1 |  |
| *Bubalornis niger* | red-billed buffalo weaver | 16 | 12.5 | 3,4 |  |
| *Buteo galapagoensis* | Galapagos hawk | 22 | 0 | 2 |  |
| *Buteo ridgwayi* | Ridgway's hawk | 79 | 0 | 1 |  |
| *Buteo swainsoni* | Swainson’s hawk | 56 | 5.4 | 1 |  |
| *Calamospiza melanocorys* | lark bunting | 455 | 25.1 | 1 |  |
| *Calcarius pictus* | Smith´s longspur | 114 | 0.9 | 3 |  |
| *Calidris alba* | sanderling | 342 | 2 | 1 |  |
| *Calidris maritima* | purple sandpiper | 82 | 1.2 | 1 |  |
| *Calidris mauri* | western sandpiper | 61 | 6.6 | 1 |  |
| *Calocitta formosa* | white-throated magpie-jay | 105 | 33.3 | 4 |  |
| *Calonectris diomedea* | Cory's shearwater | 149 | 5.4 | 1 |  |
| *Campylorhynchus griseus* | bicolored wren | 222 | 2.3 | 4 |  |
| *Campylorhynchus nuchalis* | stripe-backed wren | 69 | 1.4 | 4 |  |
| *Cardinalis cardinalis* | Northern cardinal | 37 | 13.5 | 1 | forest |
| *Carduelis cannabina* | Eurasian linnet | 106 | 3.8 | 1 |  |
| *Carduelis tristis* | American goldfinch | 70 | 14.3 | 1 | forest |
| *Carpodacus erythrinus* | scarlet rosefinch | 496 | 16.1 | 1 | forest |
| *Carpodacus mexicanus* | house finch | 331 | 8.8 | 1 | forest |
| *Catharacta lonnbergi* | brown skua | 45 | 0 | 1 |  |
| *Catharacta maccormicki* | south polar skua | 14 | 7.1 | 1 |  |
| *Centropus grillii* | black coucal | 127 | 14.2 | 2 |  |
| *Centropus phasianinus* | pheasant coucal | 59 | 18.6 | 1 |  |
| *Cepphus grylle* | black guillemot | 46 | 0 | 1 |  |
| *Cercomacra tyrannina* | dusky antbird | 15 | 0 | 1 | forest |
| *Charadrius alexandrinus* | Kentish plover | 426 | 0.9 | 1 |  |
| *Charadrius falklandicus* | two-banded plover | 15 | 0 | 1 |  |
| *Charadrius hiaticula* | ringed plover | 50 | 0 | 1 |  |
| *Charadrius marginatus* | white-fronted plover | 17 | 0 | 1 |  |
| *Charadrius modestus* | rufous-chested dotterel | 14 | 0 | 1 |  |
| *Charadrius nivosus* | snowy plover | 201 | 0 | 1 |  |
| *Charadrius pecuarius* | Kittlitz's plover | 18 | 0 | 1 |  |
| *Charadrius ruficapillus* | red-capped plover | 7 | 0 | 1 |  |
| *Charadrius semipalmatus* | semipalmated plover | 85 | 4.7 | 1 |  |
| *Charadrius thoracicus* | Madagascar plover | 20 | 0 | 1 |  |
| *Chen caerulescens* | lesser snow goose | 80 | 5 | 1 |  |
| *Chen rossii* | Ross's goose | 83 | 2.4 | 1 |  |
| *Chlidonias hybrida* | whiskered tern | 37 | 8.1 | 1 |  |
| *Chlidonias niger* | North American black tern | 28 | 0 | 1 |  |
| *Chrysococcyx basalis* | Horsfield's bronze-cuckoo | 42 | 0 | 5 |  |
| *Chthonicola sagittatus* | speckled warbler | 103 | 10.7 | 2 |  |
| *Cinclus cinclus* | white-throated dipper | 185 | 1.6 | 1 | forest |
| *Circus pygargus* | Montagu's harrier | 32 | 3.1 | 1 |  |
| *Colaptes auratus* | northern flicker | 367 | 0.3 | 2 |  |
| *Colaptes campestris* | Campo Flicker | 90 | 1.1 | 4 |  |
| *Coracias garrulus* | European roller | 169 | 5.3 | 1 |  |
| *Coragyps atratus* | black vulture | 36 | 0 | 1 |  |
| *Corcorax melanorhamphos* | white-winged chough | 68 | 0 | 4 |  |
| *Corvus brachyrhynchos* | American crow | 202 | 10.4 | 4 |  |
| *Corvus monedula* | Eurasian jackdaw | 113 | 0.9 | 1 | forest |
| *Crotophaga major* | greater ani | 357 | 13.7 | 3 |  |
| *Cyanocitta stelleri* | Steller’s jay | 79 | 15.2 | 1 | forest |
| *Cyanocorax morio* | brown jay | 50 | 16.8 | 4 |  |
| *Cyanoliseus patagonus* | burrowing parakeet | 166 | 0 | 1 |  |
| *Cygnus atratus* | black swan | 332 | 15.1 | 1 |  |
| *Dacelo novaeguineae* | laughing kookaburra | 140 | 0 | 4 |  |
| *Delichon urbicum* | house martin | 208 | 18.8 | 1 |  |
| *Dendrocopos major* | great spotted woodpecker | 161 | 0 | 1 |  |
| *Dendrocopos medius* | middle spotted woodpecker | 61 | 0 | 1 |  |
| *Dendroica caerulescens* | black-throated blue warbler | 410 | 22.7 | 1 | forest |
| *Dendroica pensylvanica* | chestnut-sided warbler | 95 | 47.4 | 1 | forest |
| *Dendroica petechia* | yellow warbler | 687 | 31 | 1 | forest |
| *Diomedea exulans* | wandering albatross | 425 | 15.8 | 1 |  |
| *Dumetella carolinensis* | grey catbird | 455 | 13 | 1 |  |
| *Eclectus roratus* | Eclectus parrot | 198 | 8.1 | 4 |  |
| *Elaenia chiriquensis* | lesser elaenia | 38 | 36.8 | 1 | forest |
| *Elaenia flavogaster* | yellow-bellied elaenia | 24 | 4.2 | 1 | forest |
| *Emberiza citrinella* | yellowhammer | 123 | 37.4 | 1 | forest |
| *Emberiza schoeniclus* | reed bunting | 2525 | 40.4 | 1 | reed |
| *Empidonax minimus* | least flycatcher | 86 | 34.9 | 1 | forest |
| *Empidonax traillii* | willow flycatcher | 140 | 14.3 | 1 |  |
| *Empidonax virescens* | acadian flycatcher | 190 | 38.9 | 1 | forest |
| *Erythropygia coryphaeus* | Karoo scrub-robin | 33 | 18.2 | 4 |  |
| *Erythrura gouldiae* | gouldian finch | 232 | 8.6 | 1 | forest |
| *Eudromias morinellus* | Eurasian dotterel | 44 | 4.5 | 1 |  |
| *Eudyptes pachyrhynchus* | Fiordland penguin | 33 | 0 | 1 |  |
| *Eudyptes schlegeli* | royal penguin | 26 | 3.8 | 1 |  |
| *Euplectes orix* | red bishop | 547 | 22.1 | 1 |  |
| *Falco columbarius* | merlin | 47 | 0 | 1 |  |
| *Falco eleonorae* | Eleonoras falcon | 60 | 0 | 1 |  |
| *Falco naumanni* | lesser kestrel | 96 | 7.3 | 1 |  |
| *Falco peregrinus* | peregrine falcon | 64 | 0 | 1 |  |
| *Falco sparverius* | American kestrel | 89 | 11.2 | 1 |  |
| *Falco tinnunculus* | Eurasian kestrel | 319 | 1.9 | 1 |  |
| *Ficedula albicollis* | collared flycatcher | 1906 | 21.0 | 1 | forest |
| *Ficedula hypoleuca* | pied flycatcher | 5116 | 9.4 | 1 | forest |
| *Ficedula parva* | red-breasted flycatcher | 159 | 7.5 | 1 | forest |
| *Ficedula zanthopygia* | yellow rumped flycatcher | 325 | 22.2 | 1 | forest |
| *Fratercula arctica* | Atlantic puffin | 38 | 0 | 1 |  |
| *Fregata minor* | great frigatebird | 138 | 3.6 | 1 |  |
| *Fringilla coelebs* | chaffinch | 47 | 17 | 1 | forest |
| *Fulmarus glacialis* | Northern fulmar | 28 | 0 | 1 |  |
| *Gallinula chloropus* | moorhen | 68 | 0 | 1 |  |
| *Gallinula mortierii* | Tasmanian native hen | 28 | 0 | 2,3 |  |
| *Gavia immer* | common loon | 58 | 0 | 1 |  |
| *Geospiza fortis* | medium ground-finch | 1248 | 17.1 | 1 | forest |
| *Geospiza scandens* | cactus finch | 368 | 10.3 | 1 |  |
| *Geothlypis trichas* | yellowthroat | 486 | 19.1 | 1 | reed |
| *Grallina cyanoleuca* | Australian magpie-lark | 103 | 2.9 | 1 |  |
| *Grus canadensis* | sandhill crane | 45 | 11.1 | 1 |  |
| *Guira guira* | Guira cuckoo | 99 | 11.1 | 1,2,3 |  |
| *Gymnorhina tibicen* | Australian magpie | 122 | 57.4 | 4 |  |
| *Gyps fulvus* | griffon vulture | 40 | 0 | 1 |  |
| *Habia fuscicauda* | red-throated ant-tanager | 41 | 41.5 | 1 | forest |
| *Haematopus ostralegus* | Eurasian oystercatcher | 65 | 1.5 | 1 |  |
| *Hirundo ariel* | fairy martin | 203 | 13.8 | 1 | forest |
| *Hirundo rustica* | barn swallow | 4039 | 24.3 | 1 |  |
| *Hylocichla mustelina* | wood thrush | 263 | 20.5 | 1 | forest |
| *Hymenolaimus malacorhynchos* | blue duck | 14 | 0 | 1 |  |
| *Icteria virens* | yellow-breasted chat | 13 | 30.8 | 1 | forest |
| *Icterus galbula* | Bullock´s oriole | 202 | 32.2 | 1 | forest |
| *Irediparra gallinacea* | comb-crested jacana | 35 | 2.9 | 2 |  |
| *Jabiru mycteria* | jabiru | 34 | 2.9 | 1 |  |
| *Jacana jacana* | wattled jacana | 235 | 10.2 | 2 |  |
| *Junco hyemalis* | dark-eyed junco | 2501 | 27.6 | 1 | forest |
| *Jynx torquilla* | Eurasian wryneck | 292 | 0.7 | 1 |  |
| *Lagopus lagopus* | willow ptarmigan | 256 | 9.4 | 1 |  |
| *Lagopus leucura* | white-tailed ptarmigan | 58 | 5.2 | 1 |  |
| *Laniarius atrococcineus* | crimson-breasted shrike | 74 | 18.9 | 1 | forest |
| *Lanius bucephalus* | bull-headed shrike | 99 | 10.1 | 1 | forest |
| *Lanius collurio* | red-backed shrike | 19 | 5.3 | 4 |  |
| *Lanius ludovicianus* | loggerhead shrike | 179 | 4.5 | 1 |  |
| *Lanius minor* | lesser gray shrike | 136 | 0 | 1 | forest |
| *Larus canus* | common gull | 55 | 3.6 | 1 |  |
| *Larus occidentalis* | western gull | 33 | 0 | 1 |  |
| *Larus ridibundus* | Black-headed gull | 79 | 20.3 | 1 |  |
| *Lichenostomus chrysops* | yellow-faced honeyeater | 18 | 44.4 | 1 | forest |
| *Locustella luscinioides* | Savi's warbler | 392 | 4.1 | 1 | reed |
| *Lophura leucomelanos* | kalij pheasant | 79 | 30.4 | 4 |  |
| *Loxia curvirostra* | red crossbill | 96 | 0 | 1 | forest |
| *Loxioides bailleui* | palila | 20 | 0 | 1 | forest |
| *Luscinia megarhynchos* | common nightingale | 121 | 21.5 | 1 | forest |
| *Luscinia svecica* | bluethroat | 1913 | 27.5 | 1 |  |
| *Malurus coronatus* | purple-crowned fairy-wren | 509 | 5.7 | 4 |  |
| *Malurus cyaneus* | superb fairy-wren | 2993 | 70.9 | 4 |  |
| *Malurus elegans* | red-winged fairy-wren | 979 | 57.0 | 4 |  |
| *Malurus melanocephalus* | red-backed fairy-wren | 673 | 53.8 | 4 |  |
| *Malurus splendens* | splendid fairy-wren | 386 | 42.2 | 4 |  |
| *Manorina melanocephala* | noisy miner | 85 | 5.9 | 4 |  |
| *Manorina melanophrys* | bell miner | 24 | 4.2 | 4 |  |
| *Megascops asio* | eastern screech-owl | 80 | 0 | 1 |  |
| *Melanerpes formicivorus* | acorn woodpecker | 386 | 0 | 4 |  |
| *Meleagris gallopavo* | wild turkey | 250 | 11.6 | 2 |  |
| *Melospiza georgiana* | swamp sparrow | 350 | 20.9 | 1 | forest |
| *Melospiza melodia* | song sparrow | 2934 | 27.9 | 1 | forest |
| *Miliaria calandra* | corn bunting | 44 | 4.5 | 1 |  |
| *Molothrus ater* | Brown headed-cowbird | 43 | 4.7 | 5 |  |
| *Monias benschi* | subdesert mesite | 17 | 11.8 | 3,4 |  |
| *Mycteria americana* | wood stork | 58 | 0 | 1 |  |
| *Myiopsitta monachus* | monk parakeet | 119 | 0 | 1 |  |
| *Nectarinia osea* | orange-tufted sunbird | 88 | 22.7 | 1 | forest |
| *Notiomystis cincta* | New Zealand hihi | 1760 | 65.2 | 1 | forest |
| *Oceanites oceanicus* | Wilson's storm-petrel | 63 | 0 | 1 |  |
| *Oceanodroma leucorhoa* | Leach's storm-petrel | 42 | 0 | 1 |  |
| *Oenanthe oenanthe* | northern wheatear | 267 | 16.5 | 1 |  |
| *Otus elegans* | elegant scops-owl | 200 | 0.5 | 1 |  |
| *Otus flammeolus* | flammulated owl | 37 | 0 | 1 |  |
| *Pachycephala pectoralis* | golden whistlers | 130 | 19.2 | 1 | forest |
| *Pachyptila belcheri* | thin-billed prion | 34 | 20.6 | 1 |  |
| *Panurus biarmicus* | bearded tit | 187 | 14.4 | 1 | reed |
| *Paradoxornis webbianus* | vinous-throated parrotbills | 246 | 7.7 | 1 |  |
| *Parus ater* | coal tit | 3717 | 31.2 | 1 | forest |
| *Parus atricapillus* | black-capped chickadee | 710 | 11.8 | 1 | forest |
| *Parus caeruleus* | blue tit | 9479 | 13.8 | 1 | forest |
| *Parus cristatus* | crested tit | 136 | 11 | 1 | forest |
| *Parus major* | great tit | 13954 | 9.3 | 1 | forest |
| *Parus montanus* | willow tit | 899 | 6.0 | 1 | forest |
| *Parus teneriffae* | African blue tit | 137 | 15.3 | 1 | forest |
| *Parus varius* | varied tit | 251 | 14.7 | 1 | forest |
| *Passer domesticus* | house sparrow | 6757 | 15.8 | 1 |  |
| *Passer montanus* | tree sparrow | 341 | 9.1 | 1 | forest |
| *Passerculus sandwichensis* | savannah sparrow | 907 | 44.4 | 1 |  |
| *Passerina caerulea* | blue grosbeak | 55 | 52.7 | 1 | forest |
| *Passerina cyanea* | indigo bunting | 63 | 34.9 | 1 | forest |
| *Perisoreus infaustus* | Siberian jay | 40 | 0 | 4 |  |
| *Petroica australis* | New Zealand robin | 260 | 0.4 | 1 | forest |
| *Petroica goodenovii* | red-capped robin | 240 | 22.1 | 1 | forest |
| *Petronia petronia* | rock sparrow | 458 | 27.5 | 1 |  |
| *Phainopepla nitens* | phainopepla | 48 | 0 | 1 | forest |
| *Phalacrocorax aristotelis* | shag | 161 | 9.3 | 1 |  |
| *Phalacrocorax atriceps* | imperial shag | 110 | 0 | 1 |  |
| *Phalacrocorax carbo* | great cormorant | 124 | 10.5 | 1 |  |
| *Phalaropus fulicarius* | red phalarope | 70 | 8.6 | 2 |  |
| *Phalaropus lobatus* | red-necked phalarope | 226 | 1.8 | 1 |  |
| *Philesturnus carunculatus* | Saddleback | 202 | 0 | 1 | forest |
| *Philetairus socius* | sociable weaver | 56 | 0 | 4 |  |
| *Phoebastria irrorata* | waved albatross | 170 | 17.6 | 1 |  |
| *Phoenicurus ochruros* | black redstart | 222 | 28.8 | 1 |  |
| *Phoenicurus phoenicurus* | common redstart | 253 | 2 | 1 | forest |
| *Phylidonyris pyrrhopterus* | crescent honeyeater | 19 | 57.9 | 1 | forest |
| *Phylloscopus fuscatus* | dusky warbler | 195 | 45.1 | 1 | forest |
| *Phylloscopus sibilatrix* | wood warbler | 62 | 1.6 | 1 | forest |
| *Phylloscopus trochilus* | willow warbler | 497 | 20.5 | 1 | forest |
| *Picoides borealis* | red-cockaded woodpecker | 80 | 1.3 | 4 |  |
| *Picoides tridactylus* | Eurasian three-toed woodpecker | 135 | 4.4 | 1 |  |
| *Pipilo maculatus* | spotted towhee | 575 | 26.3 | 1 | forest |
| *Piranga olivacea* | scarlet tanager | 54 | 16.7 | 1 | forest |
| *Platalea ajaja* | roseate spoonbill | 74 | 2.7 | 1 |  |
| *Plectrophenax nivalis* | snow bunting | 380 | 10.8 | 1 |  |
| *Plocepasser mahali* | white-browed sparrow weaver | 292 | 11.6 | 4 |  |
| *Pluvialis dominica* | American golden plover | 131 | 7.6 | 1 |  |
| *Poephila acuticauda* | long-tailed finch | 391 | 12.8 | 1 |  |
| *Pomatostomus temporalis* | grey-crowned babbler | 112 | 18.8 | 4 |  |
| *Porphyrio hochstetteri* | takahe | 27 | 0 | 1 |  |
| *Porphyrio porphyrio* | pukeko | 73 | 0 | 4 |  |
| *Progne subis* | purple martin | 1373 | 21.8 | 1 | forest |
| *Promerops cafer* | Cape sugarbird | 185 | 64.9 | 1 |  |
| *Prosthemadera novaeseelandiae* | tui | 163 | 55.2 | 1 | forest |
| *Prunella collaris* | Alpine accentor | 110 | 0 | 2 |  |
| *Prunella modularis* | dunnock | 421 | 11.9 | 1,2,3 |  |
| *Psaltriparus minimus* | bushtit | 59 | 0 | 4 |  |
| *Pseudopodoces humilis* | ground tit | 1318 | 9.3 | 4 |  |
| *Puffinus tenuirostris* | short-tailed shearwater | 83 | 10.8 | 1 |  |
| *Pygoscelis adeliae* | Adélie penguin | 22 | 9.1 | 1 |  |
| *Pygoscelis antarcticus* | chinstrap penguin | 76 | 0 | 1 |  |
| *Pyrrhura orcesi* | el oro parakeet | 104 | 1.0 | 4 |  |
| *Quelea quelea* | red-billed quelea | 56 | 21.4 | 1 |  |
| *Ramphocelus costaricensis* | Cherrie's tanager | 55 | 49.1 | 1 | forest |
| *Ramphocinclus brachyurus* | white-breasted thrasher | 67 | 7.5 | 4 |  |
| *Remiz coronatus* | white-crowned penduline tit | 29 | 0 | 1 | forest |
| *Remiz pendulinus* | penduline tit | 367 | 14.4 | 3 |  |
| *Rhipidura fuliginosa* | grey fantail | 49 | 55.1 | 1 | forest |
| *Riparia riparia* | sand martin | 335 | 17 | 1 |  |
| *Rissa tridactyla* | black-legged kittiwake | 119 | 0 | 1 |  |
| *Sayornis phoebe* | Eastern phoebe | 845 | 5.7 | 1 | forest |
| *Sericornis frontalis* | white-browed scrubwren | 137 | 12.4 | 4 |  |
| *Serinus canaria* | canary | 45 | 0 | 1 | forest |
| *Serinus serinus* | serin | 200 | 6.5 | 1 | forest |
| *Setophaga ruticilla* | American redstart | 428 | 31.5 | 1 | forest |
| *Sialia currucoides* | mountain bluebird | 1365 | 33.5 | 1 |  |
| *Sialia mexicana* | western bluebird | 1577 | 24.4 | 4 |  |
| *Sialia sialis* | Eastern bluebird | 83 | 8.4 | 1 | forest |
| *Sitta europaea* | European nuthatch | 188 | 9.6 | 1 | forest |
| *Sitta pusilla* | brown-headed nuthatch | 237 | 27.8 | 4 |  |
| *Spheniscus humboldti* | Humboldt penguin | 49 | 0 | 1 |  |
| *Spiza americana* | dickcissel | 218 | 38.5 | 1 |  |
| *Spizella pusilla* | field sparrow | 308 | 9.7 | 1 | forest |
| *Steganopus tricolor* | Wilson's phalarope | 43 | 0 | 1 |  |
| *Sterna hirundo* | common tern | 131 | 0.8 | 1 |  |
| *Stipiturus malachurus* | southern emu-wren | 50 | 12 | 4 |  |
| *Strix aluco* | tawny owl | 137 | 0.7 | 1 |  |
| *Struthidea cinerea* | apostlebird | 41 | 0 | 2,4 |  |
| *Struthio camelus* | ostrich | 61 | 49.2 | 2 |  |
| *Sturnus unicolor* | spotless starling | 536 | 15.7 | 1 | forest |
| *Sturnus vulgaris* | common starling | 415 | 15.4 | 1 | forest |
| *Sula dactylatra* | masked booby | 6 | 0 | 1 |  |
| *Sula granti* | Nazca booby | 32 | 0 | 1 |  |
| *Sula nebouxii* | blue-footed booby | 799 | 6.9 | 1 |  |
| *Sula sula* | red-footed booby | 14 | 0 | 1 |  |
| *Tachycineta albilinea* | mangrove swallow | 97 | 15.5 | 1 | forest |
| *Tachycineta bicolor* | tree swallow | 6011 | 48 | 1 |  |
| *Tachycineta leucorrhoa* | white rumped swallow | 342 | 56.4 | 1 |  |
| *Tachycineta meyeni* | Chilean swallow | 161 | 6.8 | 1 |  |
| *Taeniopygia guttata* | zebra finch | 398 | 1.8 | 1 |  |
| *Tetrao tetrix* | black grouse | 66 | 0 | 5 |  |
| *Thalassarche cauta* | shy albatross | 29 | 6.9 | 1 |  |
| *Thalassarche chrysostoma* | grey-headed albatross | 83 | 7.2 | 1 |  |
| *Thalassarche melanophrys* | black-browed albatross | 87 | 5.7 | 1 |  |
| *Thalassoica antarctica* | Antarctic petrel | 41 | 7.3 | 1 |  |
| *Thamnophilus atrinucha* | black-crowned antshrike | 89 | 3.4 | 1 | forest |
| *Thryothorus leucotis* | buff-breasted wrens | 53 | 3.8 | 4 |  |
| *Thryothorus ludovicianus* | Carolina wren | 84 | 0 | 1 | forest |
| *Thryothorus pleurostictus* | banded wren | 156 | 4.5 | 1 | forest |
| *Thryothorus rufalbus* | rufous-and-white wren | 158 | 1.9 | 1 | forest |
| *Tinamus major* | great tinamou | 121 | 24.0 | 2 |  |
| *Tockus monteiri* | Monteiro’s hornbill | 135 | 0 | 1 |  |
| *Troglodytes aedon* | house wren | 4916 | 14.1 | 1 | forest |
| *Troglodytes troglodytes* | Eurasian wren | 153 | 16.3 | 1 | forest |
| *Turdoides bicolor* | pied babbler | 145 | 4.8 | 4 |  |
| *Turdoides squamiceps* | Arabian babbler | 186 | 0 | 4 |  |
| *Turdus albicollis* | white-necked thrush | 22 | 18.2 | 1 | forest |
| *Turdus grayi* | clay-colored robin | 37 | 37.8 | 1 | forest |
| *Turdus migratorius* | American robin | 187 | 48.1 | 1 | forest |
| *Tyrannus forficatus* | scissor-tailed flycatchers | 168 | 48.8 | 1 | forest |
| *Tyrannus tyrannus* | Eastern kingbird | 328 | 46 | 1 | forest |
| *Tyto alba* | barn owl | 455 | 1.3 | 1 |  |
| *Upupa epops* | Eurasian hoopoe | 380 | 5 | 1 |  |
| *Uria aalge* | common murres | 77 | 7.8 | 1 |  |
| *Uria lomvia* | thick-billed murres | 27 | 7.4 | 1 |  |
| *Vanellus chilensis* | Southern lapwing | 41 | 9.8 | 4 |  |
| *Vermivora chrysoptera* | golden-winged warbler | 62 | 38.7 | 1 | forest |
| *Vireo griseus* | white-eyed vireo | 102 | 2 | 1 |  |
| *Vireo olivaceus* | red-eyed vireo | 19 | 57.9 | 1 | forest |
| *Vireo solitarius* | blue-headed vireo | 37 | 2.7 | 1 | forest |
| *Volatinia jacarina* | blue-black grassquits | 228 | 23.7 | 1 |  |
| *Wilsonia citrina* | hooded warbler | 356 | 26.7 | 1 | forest |
| *Zonotrichia albicollis* | white-throated sparrow | 1272 | 15.6 | 1 | forest |
| *Zonotrichia capensis* | rufous-collared sparrow | 47 | 46.8 | 1 |  |
| *Zonotrichia leucophrys* | white-crowned sparrow | 599 | 38.1 | 1 | forest |
| *Zosterops lateralis* | capricorn silvereye | 122 | 0 | 1 | forest |
