# Supplementary material for: Extra‐pair paternity in birds
Source: Mol Ecol. 2019 Oct 31;28(22):4864–82. doi: 10.1111/mec.15259 (PMC6899757; doi:10.1111/mec.15259)
Supplement: Supplementary file 6 [file MEC-28-4864-s006.pdf]

Supplemental Information for:

Extra-pair paternity in birds

Lyanne Brouwer[1,2,3*] & Simon C. Griffith[4]

[1]Department of Animal Ecology & Physiology, Institute for Water and Wetland Research, Radboud University, Nijmegen, The Netherlands
[2]Department of Animal Ecology, Netherlands Institute of Ecology (NIOO-KNAW), Wageningen, The Netherlands
[3]Division of Ecology and Evolution, Research School of Biology, The Australian National University, Canberra ACT 2601, Australia
[4]Department of Biological Sciences, Macquarie University, North Ryde, NSW 2109, Australia

Table of contents:

Geographic bias p2

Climate, life history, ecology and latitude p2

Statistical analyses p2

Model details p3

References p4

Geographic bias

To determine the average EPP of socially monogamous terrestrial species per continent we calculated average EPP as the average of the no. EPO divided by the no. offspring per species of studies performed within each continent. We used the following cut-off values for latitude and longitude: North-America (lat: 20° –70°, long: -150° —60°), South-America (lat: -60° –0°, long: -80° – -40°), Europe (lat: 35 –70°, long: -10° – 30°), Africa (lat: -30° –30°, long: -20° –40°), Australasia (lat: -45 –0°, long: 100° –180°). To exclude data from non-terrestrial species the following orders were excluded: Suliformes, Sphenisciformes, Procellariiformes, Pelecaniformes, Charadriiformes and Anseriformes.

Climate, life history, ecology and latitude

To investigate the role of climate, life history ecology and latitude, data were extracted from various sources. Latitude of each study was extracted from the publications, and if no data were provided the name of the study location was used to look up coordinates on Google Maps. For three studies of socially monogamous and two studies of species with other mating systems, details of the study site were too limited to derive a location from Google Maps (Lat and Long is missing, Table S1 & Table S2).

Life history parameters were extracted from the IUCN database (https://www.iucnredlist.org): maximum dispersal distance; generation length; migratory (yes/no) and coloniality (yes/no). No data was available for two species: *Parus teneriffae* and *Charadrius nivosus.*

Seasonality in temperature (standard deviation of mean monthly temperature) and rain (coefficient of variation of mean monthly rainfall) were derived from climate databases. For the majority of studies on socially monogamous species (398/415) these data were extracted from WorldClim (*8*), using the bioclimatic variables 'bio4' and 'bio15' at a resolution of 5 minutes. WorldClim data was not available for many small islands. For these studies temperature and rain seasonality were calculated from data from the nearest (within 100km distance) weather station (https://www.noaa.gov: *9*–*23*) (https://en.climate-data.org/south-america/ecuador/provincia-de-imbabura/galapagos-178817: *24*, *25*, https://cliflo.niwa.co.nz: *26*). The following studies were excluded because no climate data was available within 100km of the study location (*27*, *28*), or because they were conducted over a very large geographic range (~500-4000 km apart) (three studies concerning 5 species, *29*–*31*).

Statistical analyses

Generalized Linear Mixed Model (GLMM) were fitted using the total number of extra-pair offspring (EPO) versus the total number of within pair offspring (WPO) per species/study (thereby accounting for variation in sample size) using package lme 4 (*1*) from statistical software R (*2*).

To account for phylogenetic variation, we used R package MCMCglmm (*3*) to fit Bayesian Phylogenetic Mixed Models (BPMM) with a binomial distribution and logit link function. To account for phylogenetic uncertainty we followed a similar approach as Ross *et al.* (*4*). We downloaded 2,000 different trees for the species in our dataset from BirdTree.org (*5*) and sampled a tree from the posterior distribution of trees at iteration *t*, running the MCMC model for 1,000 iterations and saving the last MCMC sample from each run. The values of the latent variables and variance components were passed as starting values to the analysis at iteration t + 1 for which a new tree from the posterior sample was taken. This process was repeated for 2,000 iterations where we disposed of the first 300 as a burn-in. Estimates of parameters was based on the mean of posterior samples and their significance was assessed from credible intervals (CI) that did not include zero, and MCMC Pvalues < 0.05 (*3*). We used an inverse Wishart prior and confirmed that results did not change when using a parameter expanded prior. To evaluate

convergence, we assessed the mixing of MCMC chains visually and computed levels of autocorrelation, which were <0.1 for all estimated parameters (*3*). All analyses were run with the Hackett (*6*) and Ericsson backbones (*7*), but results did not qualitatively differ from each other and thus only results based on Hackett backbone are reported.

Model details

Model 1
The proportion variance explained by family and order was calculated as the sum of the variance explained by family and order divided by the total variance of a GLMM with the number of extra-pair offspring (EPO) vs no. within-pair offspring (WPO) per species fitted as a binomial response and identity of family and order included as random intercepts, using data from socially monogamous species.

Model 2
Repeatability was calculated on the full dataset in a GLMM with the no. EPO vs no. WPO fitted as binomial response with logit link and identity of species and population included as random intercepts using package Rptr (*33*). The number of parametric bootstraps was set to 1000.

Model 3
The proportion of variance at the level of species that can be attributed to populations was calculated as: var(population)+var(study)/var(species)+var(population)+var(study) from a GLMM with the no. EPO vs no. WPO fitted as binomial response with logit link and identity of species, population and study included as random intercepts. Species sampled in at least two different populations were selected whereby studies with $N_{offspring}<50$ were excluded to minimize bias due to low sample size.

Model 4
Habitat type was extracted for all passerines in our dataset from the Handbook of the Birds of the World (*34*) (See Table S1 & S2). To investigate whether passerines nesting in reed type vegetation had higher EPP compared to those nesting in forests, two groups were created: socially monogamous passerines inhabiting reed-type vegetation and socially monogamous species inhabiting forest or woodland type vegetation (see Table S5). Species which predominantly inhabited reed-beds like red-winged blackbirds were included, whereas species which sometimes use reeds but do not really nest there (e.g. tree swallows) were excluded from the analyses. A BPMM with the total number of extra-pair offspring versus the total number of within pair offspring per species was fitted as binomial response with logit link and identity of species and phylogeny included as random intercepts. Nesting vegetation type (reedbed or forest) was included as a fixed effect. Analyses were run for 2,000 different phylogenetic trees to account for phylogenetic uncertainty (see above).

Model 5
To simultaneously test for the role of latitude, climatic, ecological and life history parameters on EPP of socially monogamous species, we used a BPMM where we fitted the total EPO vs WPO per study. Identity of population, species and a phylogeny were included as random effects. Records with missing data (see above) were excluded, leaving 403 estimates of EPP (out of 415, Table S1) for analyses. We used within-group centring (*32*) to disentangle the between-species effect of latitude from a within-species effects of

latitude. Mean absolute latitude per species was included as a predictor at the species level, whereas the deviation from the species mean was included as a predictor of the population (within-species) level.

Model 6

We tested the association between latitude and EPP on social monogamous species which were sampled in at least 10 populations (N=4 species, Figure 10). For each species a GLM was fitted with the number of extra-pair offspring versus the total number of within pair offspring per study and the absolute latitude as a fixed effect. This model was tested against a null model to derive statistical significance. For pied flycatchers multiple estimates were available for two populations, for each of these two populations average EPP was calculated by total EPO/total offspring.

Results of GLM Model 6

Species	Blue tit	Great tit	Barn swallow	Pied flycatcher
Intercept	1.06	-0.41	-2.76	0.35
Latitude	-0.06 ± 0.008	-0.04 ± 0.007	0.04 ± 0.006	-0.05 ± 0.006
ΔDeviance	-59.6	-30.6	-37.1	-82.0
P	<0.001	<0.001	<0.001	<0.001

References

1. D. Bates, M. Mächler, B. Bolker, S. Walker, Fitting linear mixed-effects models using lme4. *J. Stat. Softw.* **67** (2015), doi:10.18637/jss.v067.i01.

2. R Development Core Team, R: A language and environment for statistical computing (2017), (available at URL http://www.R-project.org/).

3. J. D. Hadfield, MCMC Methods for Multi-Response Generalized Linear Mixed Models: The **MCMCglmm** *R* Package. *J. Stat. Softw.* **33** (2010), doi:10.18637/jss.v033.i02.

4. L. Ross, A. Gardner, N. Hardy, S. West, Ecology, Not the Genetics of Sex Determination, Determines Who Helps in Eusocial Populations. *Curr. Biol.* **23**, 2383–2387 (2013).

5. W. Jetz, G. H. Thomas, J. B. Joy, K. Hartmann, A. O. Mooers, The global diversity of birds in space and time. *Nature*. **491**, 444–448 (2012).

6. S. J. Hackett, R. T. Kimball, S. Reddy, R. C. K. Bowie, E. L. Braun, M. J. Braun, J. L. Chojnowski, W. A. Cox, K.-L. Han, J. Harshman, C. J. Huddleston, B. D. Marks, K. J. Miglia, W. S. Moore, F. H. Sheldon, D. W. Steadman, C. C. Witt, T. Yuri, A Phylogenomic Study of Birds Reveals Their Evolutionary History. *Science*. **320**, 1763–1768 (2008).

7. P. G. P. Ericson, C. L. Anderson, T. Britton, A. Elzanowski, U. S. Johansson, M. Källersjö, J. I. Ohlson, T. J. Parsons, D. Zuccon, G. Mayr, Diversification of Neoaves: integration of molecular sequence data and fossils. *Biol. Lett.* **2**, 543–547 (2006).

8. S. E. Fick, R. J. Hijmans, WorldClim 2: new 1-km spatial resolution climate surfaces for global land areas: NEW CLIMATE SURFACES FOR GLOBAL LAND AREAS. *Int. J. Climatol.* **37**, 4302–4315 (2017).

9. C. L. Abbott, M. C. Double, R. Gales, A. Cockburn, Copulation behaviour and paternity in shy albatrosses (Thalassarche cauta). *J. Zool.* **270**, 628–635 (2006).

10. J. J. Austin, D. T. Parkin, Low frequency of extra-pair paternity in two colonies of the socially monogamous short-tailed shearwater Puffinus tenuirostris. *Mol. Ecol.* **5**, 145–150 (1996).

11. C. Bichet, D. J. Penn, Y. Moodley, L. Dunoyer, E. Cellier-Holzem, M. Belvalette, A. Grégoire, S. Garnier, G. Sorci, Females tend to prefer genetically similar mates in an island population of house sparrows. *BMC Evol. Biol.* **14**, 47 (2014).

12. D. Blomqvist, M. Andersson, C. Küpper, I. C. Cuthill, J. Kis, R. B. Lanctot, B. K. Sandercock, T. Székely, J. Wallander, B. Kempenaers, Genetic similarity between mates and extra-pair parentage in three species of shorebirds. *Nature*. **419**, 613–615 (2002).

13. D. C. Dearborn, Sexual dimorphism, extrapair fertilizations, and operational sex ratio in great frigatebirds (Fregata minor). *Behav. Ecol.* **12**, 746–752 (2001).

14. L. Gilbert, T. Burke, A. Krupa, No evidence for extra-pair paternity in the western gull. *Mol. Ecol.* **7**, 1549–1552 (1998).

15. Y.-C. Hsu, S.-H. Li, Y.-S. Lin, M. T. Philippart, L. L. Severinghaus, High Frequency of Extra-Pair Copulation with Low Level of Extra-Pair Fertilization in the Lanyu Scops Owl Otus elegans botelensis. *J. Avian Biol.* **37**, 36–40 (2006).

16. F. A. Juola, D. C. Dearborn, Sequence-based evidence for major histocompatibility complex-disassortative mating in a colonial seabird. *Proc. R. Soc. B Biol. Sci.* **279**, 153–162 (2012).

17. J. T. Lifjeld, Ann M. A. Harding, F. Mehlum, T. Øigarden, No Evidence of Extra-Pair Paternity in the Little Auk Alle alle. *J. Avian Biol.* **36**, 484–487 (2005).

18. K. H. Maher, L. J. Eberhart-Phillips, A. Kosztolányi, N. dos Remedios, M. C. Carmona-Isunza, M. Cruz-López, S. Zefania, J. J. H. St Clair, M. Alrashidi, M. A. Weston, M. A. Serrano-Meneses, O. Krüger, J. I. Hoffman, T. Székely, T. Burke, C. Küpper, High fidelity: extra-pair fertilisations in eight *Charadrius* plover species are not associated with parental relatedness or social mating system. *J. Avian Biol.* **48**, 910–920 (2017).

19. B. C. Robertson, Genetic monogamy in the absence of paternity guards: the Capricorn silvereye, Zosterops lateralis chlorocephalus, on Heron Island. *Behav. Ecol.* **12**, 666–673 (2001).

20. D. Schamel, D. M. Tracy, D. B. Lank, D. F. Westneat, Mate Guarding, Copulation Strategies and Paternity in the Sex-Role Reversed, Socially Polyandrous Red-Necked Phalarope Phalaropus lobatus. *Behav. Ecol. Sociobiol.* **57**, 110–118 (2004).

21. I. Swatschek, D. Ristow, M. Wink, Mate fidelity and parentage in Cory's shearwater Calonectris diomedea - field studies and DNA fingerprinting. *Mol. Ecol.* **3**, 259–262 (1994).

22. K. Wojczulanis-Jakubas, D. Jakubas, T. Øigarden, J. T. Lifjeld, Extrapair copulations are frequent but unsuccessful in a highly colonial seabird, the little auk, Alle alle. *Anim. Behav.* **77**, 433–438 (2009).

23. A. G. Ramos, S. O. Nunziata, S. L. Lance, C. Rodríguez, B. C. Faircloth, P. A. Gowaty, H. Drummond, Habitat structure and colony structure constrain extrapair paternity in a colonial bird. *Anim. Behav.* **95**, 121–127 (2014).

24. D. J. Anderson, P. T. Boag, No Extra-pair Fertilization Observed in Nazca Booby (Sula granti) Broods. *Wilson J. Ornithol.* **118**, 244–247 (2006).

25. P. C. Baião, P. G. Parker, No Evidence of Extra-Pair Fertilization in Red-footed Boobies *(Sula sula)*. *Waterbirds*. **32**, 179–182 (2009).

26. I. G. McLean, S. D. Kayes, J. O. Murie, L. S. Davis, D. M. Lambert, Genetic monogamy mirrors social monogamy in the Fiordland crested penguin. *N. Z. J. Zool.* **27**, 311–316 (2000).

27. G. Ibarguchi, Male-biased Mutation Rates and the Overestimation of Extrapair Paternity: Problem, Solution, and Illustration Using Thick-Billed Murres (Uria lomvia, Alcidae). *J. Hered.* **95**, 209–210 (2004).

28. M. M. Baumgarten, A. B. Kohlrausch, C. Yumimiyaki, T. R. Ochotorena de Freitas, A. Mellender de Araujo, DNA Fingerprinting and Parentage in Masked (Sula Dactylatra) and Brown (S. Leucogaster) Boobies. *Ornitologia Neotropical*, 319–326 (2001).

29. M. E. Koopman, D. B. McDonald, G. D. Hayward, Microsatellite Analysis Reveals Genetic Monogamy Among Female Boreal Owls. *J. Raptor Res.* **41**, 314–318 (2007).

30. C. I. Miño, M. A. Russello, P. F. Mussi Gonçalves, S. N. Del Lama, Reconstructing genetic mating systems in the absence of parental information in colonially breeding waterbirds. *BMC Evol. Biol.* **11** (2011), doi:10.1186/1471-2148-11-196.

31. M. Lettink, I. G. Jamieson, C. D. Millar, D. M. Lambert, Mating system and genetic variation in the endangered New Zealand takahe. *Conserv. Genet.* **3**, 427–434 (2002).

32. M. van de Pol, J. Wright, A simple method for distinguishing within- versus between-subject effects using mixed models. *Anim. Behav.* **77**, 753–758 (2009).

33. M. A. Stoffel, S. Nakagawa, H. Schielzeth, rptR: repeatability estimation and variance decomposition by generalized linear mixed-effects models. *Methods Ecol. Evol.* **8**, 1639–1644 (2017).

34. J. del Hoyo, A. Elliott, J. Sargatal, *Handbook of the birds of the world.* (Lynx Edit., Barcelona, 1992).
